# Multifunctional, Fluorene‐Based Modulator of Cholinergic and GABAergic Neurotransmission as a Novel Drug Candidate for Palliative Treatment of Alzheimer's Disease

**DOI:** 10.1002/anie.202420510

**Published:** 2024-11-22

**Authors:** Dawid Panek, Anna Pasieka, Jakub Jończyk, Milena Gawlińska, Paula Zaręba, Agata Siwek, Małgorzata Wolak, Barbara Mordyl, Monika Głuch‐Lutwin, Gniewomir Latacz, Xavier Brazzolotto, Fabien Chantegreil, Florian Nachon, Jana Zdarova Karasova, Jaroslav Pejchal, Martin Mzik, Vit Sestak, Lukas Prchal, Jitka Odvarkova, Ondrej Soukup, Jan Korabecny, Ales Sorf, Marie Hamsikova, Lucie Zemanova, Lubica Muckova, Nela Vánova, Pola Dryja, Kinga Sałat, Georg Höfner, Klaus Wanner, Anna Więckowska, Barbara Malawska

**Affiliations:** ^1^ Department of Physicochemical Drug Analysis Faculty of Pharmacy Jagiellonian University Medical College Medyczna St. 9 30-688 Cracow Poland; ^2^ Department of Pharmacobiology Faculty of Pharmacy Jagiellonian University Medical College Medyczna St. 9 30-688 Cracow Poland; ^3^ Department of Technology and Biotechnology of Drugs Faculty of Pharmacy Jagiellonian University Medical College Medyczna St. 9 30-688 Cracow Poland; ^4^ Département de Toxicologie et Risques Chimiques Institut de Recherche Biomédicale des Armées 91223 Brétigny sur Orge France; ^5^ Department of Toxicology and Military Pharmacy Military Faculty of Medicine University of Defence in Brno Trebesska 1575 500 02 Hradec Kralove Czech Republic; ^6^ Biomedical Research Center University Hospital Hradec Kralove Sokolska 581 500 05 Hradec Kralove Czech Republic; ^7^ Department of Clinical Biochemistry University Hospital Hradec Kralove Sokolska 581 500 05 Hradec Kralove Czech Republic; ^8^ Department of Social and Clinical Pharmacy Faculty of Pharmacy in Hradec Kralove Charles University Akademika Heyrovskeho 1203 Hradec Kralove Czech Republic; ^9^ Department of Chemistry Faculty of Science University of Hradec Kralove Rokitanskeho 62 500 03 Hradec Kralove Czech Republic; ^10^ Department of Pharmaceutical Chemistry and Pharmaceutical Analysis Faculty of Pharmacy in Hradec Kralove Charles University Akademika Heyrovskeho 1203 Hradec Kralove 500 05 Czech Republic; ^11^ Department of Pharmacodynamics Chair of Pharmacodynamics Faculty of Pharmacy Jagiellonian University Medical College Medyczna St. 9 30-688 Cracow Poland; ^12^ Department of Pharmacy – Center for Drug Research Ludwig-Maximilians-Universität München Butenandtstr. 5–13 81377 Munich Germany; ^13^ Sano Centre for Computational Medicine Czarnowiejska 36 30-054 Cracow Poland

**Keywords:** Alzheimer's disease, butyrylcholinesterase, GABA transporters, inhibitors, multitarget drugs

## Abstract

Alzheimer's disease (AD) is a neurodegenerative disorder characterized by memory loss and behavioral and psychological symptoms of dementia (BPSD). Given that cholinergic neurons are predominantly affected in AD, current treatments primarily aim to enhance cholinergic neurotransmission. However, imbalances in other neurotransmitters, such as γ‐aminobutyric acid (GABA), also contribute to AD symptomatology. In the presented research, using a combination of crystallography and computational methods we developed compound **6** as a dual modulator of GABAergic and cholinergic neurotransmission systems. Compound **6** demonstrated inhibition of BuChE (IC_50_=0.21 μM) and GABA transporter 1 (IC_50_=10.96 μM) and 3 (IC_50_=7.76 μM), along with a favorable drug‐likeness profile. Subsequent in vivo studies revealed the effectiveness of **6** in enhancing memory retention and alleviating anxiety and depression symptoms in animal models, while also proving safe and bioavailable for oral administration. The innovative multi‐target‐directed ligand **6** offers a new approach to treating cognitive deficits and BPSD in AD.

## Graphical abstract

Patients with Alzheimer's disease need immediate help. In this work, we present a new approach to treating cognitive deficits alongside the psychological symptoms of dementia. Through a combination of biological screening, crystallography, and computational methods, we have developed an innovative drug candidate – a dual modulator of cholinergic and GABAergic neurotransmission. Comprehensive in vivo evaluation demonstrates its strong potential for a breakthrough.

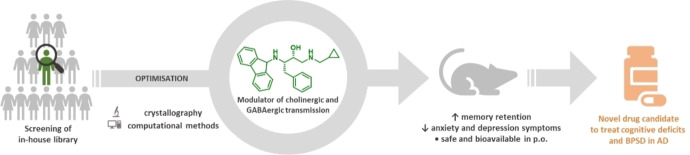

Alzheimer's disease (AD) is an incurable chronic neurodegenerative disorder of the central nervous system (CNS), which is characterized by memory loss, disorientation and deficits in cognitive functions.^[1]^ Apart from memory‐related symptoms, AD is accompanied by behavioral and psychological symptoms of dementia (BPSD), which include agitation, aggression, depression, anxiety and sleep disturbances.^[2]^ The progression of these symptoms is attributed to the deterioration of nerve connections and compromised neurotransmission. This is particularly relevant to the cholinergic system, which plays a pivotal role in memory processes.[^3^, ^4^] Hence, current pharmacotherapy primarily focuses on enhancing cholinergic neurotransmission by inhibiting enzymes responsible for acetylcholine degradation, namely acetylcholinesterase (AChE) and butyrylcholinesterase (BuChE).^[5]^ Nevertheless, additional neurotransmitter systems, such as γ‐aminobutyric acid (GABA), are also disrupted, leading to an imbalance between excitation and inhibition in neuronal circuits.[^6^, ^7^] Considering the significant impact of both the cholinergic and GABAergic systems in the etiopathogenesis of AD, we hypothesize that simultaneous modulation of both systems could potentially offer a more effective palliative treatment for AD compared to current pharmacotherapy.^[8]^ GABAergic neurotransmission is primarily regulated by sodium symporters within the solute carrier 6 (SLC6) family, including GAT1, BGT1, GAT2, and GAT3 with GAT1 and GAT3 located in the CNS. According to current research, GABA stands out as a significant contributor to the emergence of specific BPSD symptoms and memory impairment. This observation is substantiated by the anxiolytic‐like, sedative, and antidepressant‐like activities of GAT1 inhibitors: tiagabine^[9]^ and DDPM‐2571.^[10]^ On the other hand, administration of a non‐selective GAT2/GAT3 inhibitor, (*S*)‐SNAP‐5114, effectively mitigates memory deficits in the 5xFAD mouse model of AD.^[11]^

Herein, we present a first‐in‐class multi‐target‐directed ligand (MTDL) modulating cholinergic and GABAergic neurotransmission systems with proven enhancement of memory retention and alleviation of BPSD in animal models. In recent studies, we conducted a comprehensive biological screening of an in‐house library of BuChE inhibitors, targeting all GAT subtypes.^[12]^ This effort resulted in the identification of compound I which demonstrated selective inhibition of BuChE (*eq*BuChE IC_50_=3 μM) over AChE as well as selective inhibition of GAT3 (mGAT4 IC_50_=5 μM, *h*GAT‐3 IC_50_=3 μM; both subtypes correspond to each other, differences in names between species). Its activity towards GAT3 was higher than the most potent inhibitors reported up to date, such as (*S*)‐SNAP‐5114,^[13]^ with IC_50_ of 45 μM against *h*GAT‐3^[14]^ (mGAT4 IC_50_=2 μM),^[15]^ and lack of selectivity. Most importantly, low micromolar in vitro potencies of GAT3 inhibitors are sufficient to translate into the in vivo effects, e.g. (*S*)‐SNAP‐5114 showed memory enhancement, along with antinociceptive and anticonvulsant activity in animal models.[^11^, ^16^, ^17^]

Despite encouraging evidence from in vitro studies we did not observe pro‐cognitive effects of compound I in a passive avoidance (PA) task. At this point, we focused our efforts on the improvement of in vitro potency towards BuChE and optimization of physicochemical properties. In terms of the latter, we modified the structure to reduce: i) molecular weight from 478.6 Da to values below 400, ii) logP from 5.7 to values below 5, iii) number of aromatic rings (n_ar_) from 4 to 3, iv) flexibility represented by the number of rotatable bonds (n_rot.bonds_) to below 10 (Figure 1).

**Figure 1:**
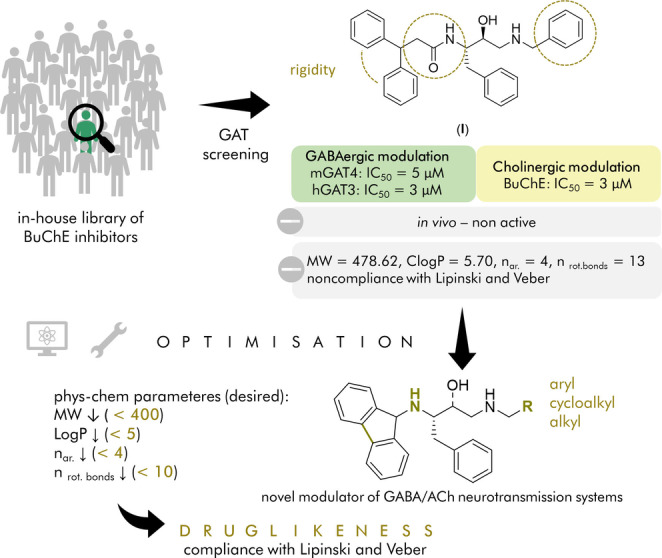
Identification and optimization of the “hit compound (**I**)” leading to **6**.

Our initial optimization efforts for compound I focused on enhancing structural rigidity by reducing the number of rotatable bonds. To achieve this goal, we designed a tricyclic ring system through cyclization of a benzhydryl fragment. We systematically screened structural fragments found in known GAT inhibitors (e.g. carbazole,^[18]^ fluorene,^[19]^ dibenzazepine and phenothiazine)^[20]^ and BuChE inhibitors (e.g. phenothiazine,^[21]^ carbazole).^[22]^ Based on molecular modeling studies, we identified fluorene as the most promising substituent (Supporting Information). In the binding mode of compound I analog co‐crystallized with BuChE (PDB: 7AMZ) we observed that the benzyl fragment does not exhibit proper functionality due to its imperfect alignment and overlap with the benzhydryl moiety in the enzyme's peripheral active site. Furthermore, based on molecular dynamic studies, we identified that shortening the molecule by removing the carbonyl group while preserving the amine effectively orients the benzyl fragment within the choline‐binding pocket, thereby reinforcing interactions in the active site (Supporting Information; Figures 19 and 20). Finally, to decrease the number of aromatic rings and thus lipophilicity, we intended to replace the benzyl group with a cycloalkylmethanamine moiety. The designed compounds (**1**–**6**) were prepared as pure diastereoisomers according to the synthetic pathway presented in Scheme 1.

**Scheme 1:**
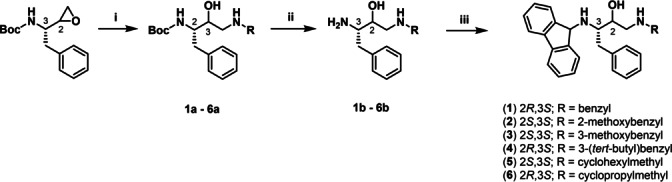
Reagents and conditions: (i) appropriate amine, pyridine, *i*‐propanol, reflux, 16 h; (ii) TFA, DCM, RT, 2 h; (iii) 9‐bromofluorene, K_2_CO_3_, MeCN, RT, 16 h.

The prepared compounds exhibited selective inhibition of BuChE over AChE and non‐selectively inhibited GAT3 compared to other subtypes (Table 1) which, particularly with regard to GAT1 inhibition, may be beneficial in terms of BPSD, as in the case of tiagabine. From the tested compounds, we identified **6** with an IC_50_ against *h*BuChE: 0.21 μM, mGAT1: 10.96 μM and mGAT4: 7.76 μM, demonstrating activity comparable to compound I. Notably, compound **6** showed improved drug‐likeness, characterized by favorable physicochemical properties (MW<400, logP<5, n_ar_<4, and n_rot.bonds_<10), high metabolic stability (90 % remains after 120 min incubation with human liver microsomes) and no significant increase in the intracellular level of reactive oxygen/nitrogen species (RONS) or malondialdehyde (MDA). While compound **6** is a CYP2D6 inhibitor (Supporting Information), this is not a major concern, as many marketed drugs share this trait.^[24]^ Nonetheless, it will require attention during further development due to the potential for drug‐drug interactions.

**Table 1:** Inhibition of BuChE and GATs.

Cmp	*eq*BuChE^[a]^	*h*BuChE^[a,b]^	*m*GAT1^[c]^	*m*GAT2^[c]^	*m*GAT3^[c]^	*m*GAT4^[c]^
	IC_50_ [μM]/ % inh.		IC_50_ [μM]/ % inh.^[c]^			
**1**	0.63±0.02	0.20±0.00	10.96	9.77±1.82	7.08±1.15	7.76±1.99
**2**	0.28±0.01	0.27±0.01	6.31±1.02	13.18	65 %	6.17±0.57
**3**	2.20±0.10	67.7 % ±2.7	7.41±0.34	12.02	66 %	7.08±0.49
**4**	0.69±0.04	68.0 % ±1.7	14.79	26.30	8.91±1.03	56 %
**5**	0.96±0.03	76.5 % ±1.7	54 %	10.72	11.48	59 %
**6**	0.31±0.01	0.21±0.01	10.96	10.72	7.41±0.69	7.76±0.36
**Compound I**	2.94±0.11	67.7 %±0.9	61 %	74 %	76 %	5.01±1.1
**Tacrine**	0.02±0.00	0.03±0.00				
**Rivastigmine**	2.20±0.05					
**(*S*)‐SNAP‐5114** ^[23]^			85±0.09	56 %	5.13±0.04	1.95±0.07

[a] means±SEM of at least three experiments, each in triplicate, [b] %inh at an inhibitor concentration of 1 μM, [c] %inh represents the [^3^H]GABA uptake or NO‐711 binding in the presence of 100 μM inhibitor; data without SEM: one experiment in triplicate. IC_50_ are from the GABA uptake assays (means±SEM, *n*=3 for values above 10 μM, *n*=1 for values between 10 and 100 μM).

The structure of **6** bound to *h*BuChE was determined by X‐ray crystallography after protein‐ligand complex formation by crystal soaking (Supporting Information). The proximal ring of the fluorene engages in a dense π–π interaction network with Trp231 (5.5 Å), Phe329 (5.4 Å) and Phe398 (6.5 Å), which corresponds to benzhydryl fragment of the analog of compound I. As we expected, the shortened molecule redirected the benzyl group's orientation into a choline‐binding pocket, producing π–π interactions (4.0 Å) with Trp82. The amine nitrogen close to the fluorene forms an H‐bond with Pro285 carbonyl oxygen atom, while the hydroxyl engages both Asp70 and Tyr332, forming the BuChE peripheral site, with respective distances of 4.0 and 4.4 Å. These observations confirm that we have effectively combined crystallography with a molecular dynamics study to formulate the correct hypothesis.

Due to its compelling in vitro activity profile, **6** was selected for in‐depth in vivo studies starting with acute toxicity assessment. During evaluation at several doses administered intraperitoneally, the maximal tolerated dose (MTD) was estimated at 10 mg/kg. At this dose, we observed mild signs of poisoning except for one female subgroup having moderate tremors. Autopsy at this dose revealed no macroscopic pathology of internal organs of the chest and abdominal cavity, except for mild hyperemia in the peritoneal cavity which may be due to the i. p. route of administration. Histopathological examination of the liver and kidney showed no tissue damage, only occasional neutrophils in subcapsular liver tissue and perirenal fat neighboring the peritoneal cavity in one male and one female mouse (Supporting Information; Table 17).

Following i. p. administration **6** showed a beneficial pharmacokinetic (PK) profile in mice with moderately slow elimination (t_1/2_~161 min) and distribution throughout the total body water (V_D_~41 L/kg). A maximal concentration of 430 ng/mL was reached within 23 minutes. The PK study evidenced high brain penetration of **6**, with a maximal brain concentration of 833 ng/g reached 55 minutes after administration, and a brain‐to‐plasma ratio of 11 (Figure 2A, Supporting Information; Table 13).

**Figure 2:**
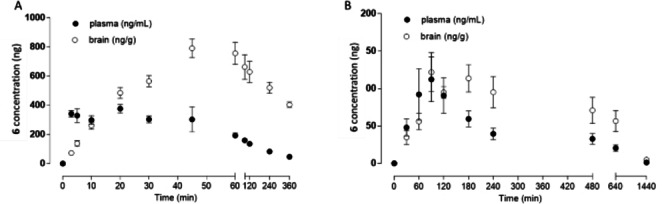
Levels of **6** measured in mice plasma (•) and brain (○) collected at various times up to 6 hours after a single i. p. dose (10 mg/kg) (A) and i.g. dose (10 mg/kg) (B), (n=6).

Finally, we verified the initial hypothesis and evaluated the activity of **6** as a dual cholinergic‐GABAergic modulator for the improvement of memory and BPSD‐related symptoms, including anxiety and depression. Memory enhancement after administration of **6** was tested in vivo using the PA task and Barnes maze (BM) test in mice treated with scopolamine. At a dose of 10 mg/kg in the PA task we observed prolonged step‐thorough latency in the retention trial compared to the scopolamine‐treated group (Supporting Information; Figure 53). In the acquisition trial of the BM test, mice treated with **6** and scopolamine showed a statistically significantly reduced latency to target (Figure 3A), when compared to the control group receiving scopolamine. Most importantly, in the retention trial of the BM task carried out on day 12, **6** significantly shortened the latency to former target location (Figure 3C) and reduced the number of errors (Figure 3E).

**Figure 3:**
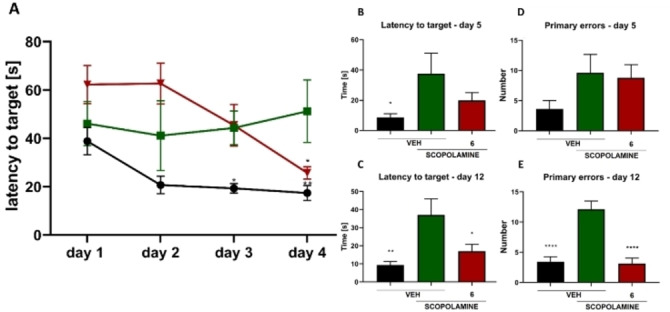
Effect of **6** on spatial learning assessed in a BM test during the acquisition phase (days 1–4) (A) on memory retrieval on days 5 (B, D) and 12 (C, E). Data expressed as the mean latency to find the escape box [s]±SEM or the mean number of errors made±SEM for n=7–12. Statistical analysis: repeated measures ANOVA and Dunnett's post hoc analysis. Significance vs. scopolamine‐treated control group: *p<0.05, **p<0.01, ****p<0.0001.

We chose anxiety and depression as the primary symptoms due to their prevalence and significant effect on normal functioning of patients with AD, particularly those associated with GAT‐related pathomechanisms. In the four‐plate test (FPT), a statistically significant increase in the number of punished crossings was observed at doses of 0.1, 1, and 10 mg/kg compared to the control group (Figure 4C). Likewise, in the elevated plus maze, **6** demonstrated significant prolongation of time spent in the open arms at doses of 0.1 and 1 mg/kg, along with an increased number of entries into the open arms at doses of 1 and 10 mg/kg, compared to the control group (Figure 4A and 4B). Both in vivo tests revealed potent anxiolytic‐like activity of **6**. Notably, this effect was observed even at a very low dose of 0.1 mg/kg, underscoring its efficacy. We tried to find another mechanism that may harmonize with GAT inhibition generating an anxiolytic‐like effect (Supporting Information). We ruled out interactions of **6** with molecular targets most strongly related to anxiety, i.e. receptors: GABA‐A, 5‐HT_1A_, 5‐HT_6_, 5‐HT_7_, and serotonin transporter (SERT); however, the anxiolytic effect may be strengthened by modulation of norepinephrine transporter (NET) (Supporting Information). Compound **6** also exhibited mild yet statistically significant antidepressant activity at a dose of 1 mg/kg in the forced swim test (Figure 4D).

**Figure 4:**
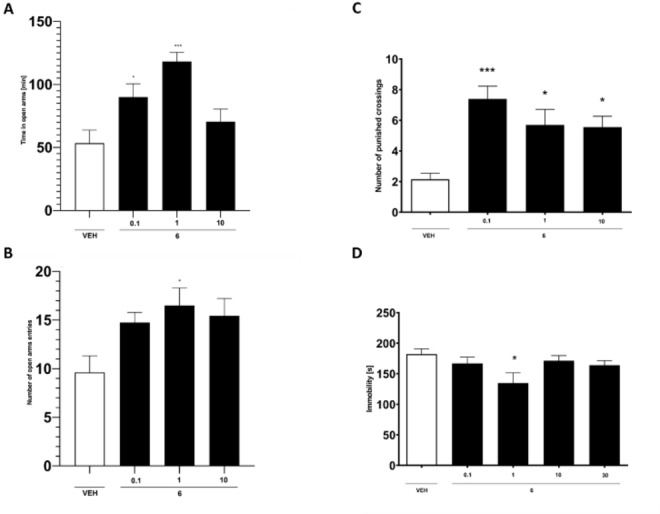
Effect of **6** (0.1, 1 and 10 mg/kg, i. p.) on time [s] (±SEM) spent in open arms of the elevated plus maze (A), number of open arms entries (±SEM) (B), number of punished crossings measured using FPT in mice (±SEM) (C) and duration of immobility measured using FST in mice (D). n=7–10, statistical analysis: one‐way ANOVA followed by Dunnett's post hoc comparison. Significance vs. control: *p<0.05, ***p<0.001.

Since the oral route of administration is preferred for anti‐AD drug candidates, we confirmed **6**’s safety and PK at dose of 10 mg/kg after i.g. administration. At this dose, no signs of toxicity occurred, necropsy revealed no pathology of internal organs of the chest and abdominal cavity, as well as a lack of significant changes in blood biochemistry. Similar to i. p. administration, the time‐dependent plasma profile comprised a short invasive phase with increasing concentrations up to 120 ng/mL achieved within 80 min, followed by subsequent gradual exponential bio‐elimination (half‐life of 212 min). The early T_max_ indicates relatively fast absorption from initial parts of the small intestine. The pharmacokinetic study revealed permeation of **6** into the CNS with a maximal concentration of 159 ng/mL reached within 200 min and a brain‐to‐plasma ratio of approximately 2 (Supporting Information). Due to its favorable PK after i.g. administration, **6** may be considered a promising candidate for oral administration.

Our study presents a significant advancement in the development of therapies for AD through synthesis and evaluation of **6**. It demonstrated selective inhibition of *h*BuChE (IC_50_=0.21 μM) alongside inhibition of mGAT1 (IC_50_=10.96 μM) and mGAT4 (IC_50_=7.76 μM). It furthermore showed a promising PK profile with good bioavailability and effective CNS penetration with a brain concentration (C_max_) of 833 ng/g and a brain‐to‐plasma ratio of 11, following i. p. administration. The safety of **6** was also confirmed in mice at a dosage of 10 mg/kg, showing no significant toxicity or pathological changes. Notably, in vivo studies revealed its potential in treating AD, with substantial enhancement of memory retention in the PA and BM tasks, as well as a reduction in anxiety‐like and depression‐like behavior in mice. Compound **6**, as described herein, is a cholinergic‐GABAergic modulator with unique pharmacodynamic properties aimed at managing cognitive deficits and BPSD symptoms in Alzheimer′s disease.

## Conflict of Interests

The authors declare no conflict of interest.

## Supporting information

As a service to our authors and readers, this journal provides supporting information supplied by the authors. Such materials are peer reviewed and may be re‐organized for online delivery, but are not copy‐edited or typeset. Technical support issues arising from supporting information (other than missing files) should be addressed to the authors.

Supporting Information

## Data Availability

The data that support the findings of this study are available in the supplementary material of this article.
