# Supplementary material for: Multifunctional, Fluorene‐Based Modulator of Cholinergic and GABAergic Neurotransmission as a Novel Drug Candidate for Palliative Treatment of Alzheimer's Disease
Source: Angew Chem Int Ed Engl. 2024 Nov 22;64(6):e202420510. doi: 10.1002/anie.202420510 (PMC11796312; doi:10.1002/anie.202420510)
Supplement: Supplementary file 1 — Supporting Information [file ANIE-64-e202420510-s001.pdf]

Angewandte
Chemie
Eine Zeitschrift der Gesellschaft Deutscher Chemiker

Supporting Information

Multifunctional, Fluorene-Based Modulator of Cholinergic and GABAergic Neurotransmission as a Novel Drug Candidate for Palliative Treatment of Alzheimer's Disease

D. Panek, A. Pasieka, J. Jończyk, M. Gawlińska, P. Zaręba, A. Siwek, M. Wolak, B. Mordyl, M. Głuch-Lutwin, G. Latacz, X. Brazzolotto, F. Chantegreil, F. Nachon, J. Zdarova Karasova, J. Pejchal, M. Mzik, V. Sestak, L. Prchal, J. Odvarkova, O. Soukup, J. Korabecny, A. Sorf, M. Hamsikova, L. Zemanova, L. Muckova, N. Vánova, P. Dryja, K. Sałat, G. Höfner, K. Wanner, A. Więckowska, B. Malawska*

Supporting information

Multifunctional, fluorene-based modulator of cholinergic and GABA-ergic neurotransmission as a novel drug candidate for palliative treatment of Alzheimer's disease.

Dawid Panek, Anna Pasieka, Jakub Jończyk, Milena Gawlińska, Paula Zaręba, Barbara Mordyl, Monika Głuch-Lutwin, Gniewomir Latacz, Xavier Brazzolotto, Fabien Chantegreil, Florian Nachon, Jana Zdarova Karasova, Jaroslav Pejchal, Martin Mzik, Vit Sestak, Lukas Prchal, Jitka Odvarkova, Ondrej Soukup, Jan Korabecny, Ales Sorf, Marie Hamsikova, Lucie Zemanova, Ľubica Múčková, Nela Váňová, Pola Dryja, Kinga Sałat, Georg Höfner, Klaus Wanner, Anna Więckowska, Barbara Malawska

Synthesis and spectroscopic data

The initial step was the nucleophilic substitution of 2*S*,3*S* or 2*R*,3*S* epoxide with the appropriate amine in the presence of catalytic amounts of pyridine. The reaction follows an SN_2 mechanism, yielding pure chiral amino alcohols **1a**–**6a**. Subsequently, they underwent BOC deprotection and were alkylated with 9-bromofluorene to produce the final compounds **1**–**6**. The final compounds **1**–**6** were obtained as pure diastereoisomers.

General Synthesis

All reagents were purchased from commercial suppliers and were used without further purification. Dichloromethane (DCM) was distilled under nitrogen immediately before use. The drying agent used for DCM was calcium hydride. Reactions were monitored by thin-layer chromatography carried out on aluminium sheets precoated with silica gel 60 F_{254} (Merck). For the TLC and flash chromatography, the following solvents were used: dichloromethane (DCM), methanol (MeOH), chloroform ($CHCl_3$), ethyl acetate (EtOAc), petroleum ether (PET), 25% ammonia−water solution. Flash chromatography was performed on Isolera™ Spectra (Biotage) using Merck silica gel 60 (63−200 μm) as a stationary phase. The reverse-phase HPLC purification was performed on LC-4000 Jasco using a Phenomenex Luna C8 (5 μm, 15 x 21.2 mm) column and CH_3CN/H_2O gradient with 0.1% HCOOH as a mobile phase. The purity of the final compounds was determined using an analytical RPLC-MS on Waters Acquity TQD using an Aquity UPLC BEH C18 column (1.7 μm, 2.1 × 100 mm) at 214 and 254 nm. The CH_3CN/H_2O gradient with 0.1% HCOOH was used as the mobile phase at a flow rate of 0.3 mL/min. All the compounds showed purity above 95%. ^1H NMR and ^{13}C NMR spectra were recorded on Jeol 500 MHz. The chemical shifts are reported in ppm and were referenced to residual solvent signals (^1H, $CDCl_3$ at 7.26 ppm; ^{13}C, $CDCl_3$ at 77.2 ppm). Signal multiplicities are represented by the following abbreviations: s (singlet), br. s (broad singlet), d (doublet), dd (doublet of doublets), ddd (doublet of doublets), dt (doublet of triplets), t (triplet), br. t (broad triplet), td (triplet of doublets), tdd (triplet of doublet of doublets), tt (triplet of triplets), q (quartet), dq (doublet of quartets), qd (quartet of doublets), and m (multiplet). Mass spectra (MS) were recorded on UPLC-MS/MS system consisting of a Waters ACQUITY UPLC (Waters Corporation, Milford, MA, USA) coupled to a Waters

TQD mass spectrometer (electrospray ionization mode ESI-tandem quadrupole). All the compounds showed purity above 95%.

The following compounds: *tert*-butyl ((2*S*,3*R*)-4-(benzylamino)-3-hydroxy-1-phenylbutan-2-yl)carbamate (**1a**),[25] *tert*-butyl ((2*S*,3*S*)-3-hydroxy-4-((2-methoxybenzyl)amino)-1-phenylbutan-2-yl)carbamate (**2a**), [25] *tert*-butyl ((2*S*,3*R*)-4-((3-(*tert*-butyl)benzyl)amino)-3-hydroxy-1-phenylbutan-2-yl)carbamate (**4a**),[25] *tert*-butyl ((2*S*,3*R*)-4-((cyclopropylmethyl)amino)-3-hydroxy-1-phenylbutan-2-yl)carbamate (**6a**),[25] (2*R*,3*S*)-3-amino-1-(benzylamino)-4-phenylbutan-2-ol (**1b**),[25] (2*S*,3*S*)-3-amino-1-((2-methoxybenzyl)amino)-4-phenylbutan-2-ol (**2b**),[25] (2*R*,3*S*)-3-amino-1-((3-(*tert*-butyl)benzyl)amino)-4-phenylbutan-2-ol (**4b**),[25] (2*R*,3*S*)-3-amino-1-((cyclopropylmethyl)amino)-4-phenylbutan-2-ol (**6b**),[25]have been previously reported.

Procedure for the synthesis of compounds 3a and 5a (procedure A):

Tert-butyl ((*S*)-1-((*S*)-oxiran-2-yl)-2-phenylethyl)carbamate or *tert*-butyl ((*S*)-1-((*R*)-oxiran-2-yl)-2-phenylethyl)carbamate (1.0 equiv.), corresponding amine (1.1 equiv.), and a catalytic amount of pyridine in isopropanol were refluxed for 16 h. Then, the solvent was evaporated and the resulting residue was purified by flash column chromatography using a mixture of DCM and MeOH (gradient or isocratic purification).

tert-butyl ((2*S*,3*S*)-3-hydroxy-4-((3-methoxybenzyl)amino)-1-phenylbutan-2-yl)carbamate (3a)

The reaction of *tert*-butyl ((*S*)-1-((*R*)-oxiran-2-yl)-2-phenylethyl)carbamate (2.000 g, 7.60 mmol) with 3-methoxybenzylamine (1.042 g, 7.60 mmol) in the presence of catalytic amount of pyridine in isopropanol (20 mL) was performed according to the **procedure A**. Purification: flash column chromatography (5% MeOH in DCM). Yield: 0.436 g (57.3%), TLC (DCM/MeOH, 9/1 *v/v*) $R_f = 0.37$, MW 400.52, formula: $C_{23}H_{32}N_2O_4$, MS *m/z* 401.4 ($M+H^+$), ^{1}H NMR (500 MHz, CHLOROFORM-*d*) δ ppm 7.17 - 7.37 (m, 6H), 6.86 - 6.92 (m, 2H), 6.81 (dd, *J* = 8.02, 2.29 Hz, 1H), 4.68 (br. s, 1H), 3.73 - 3.84 (m, 5H), 3.48 – 3.55 (m, 1H), 2.97 (dd, *J* = 14.03, 4.87 Hz, 1H), 2.66 - 2.90 (m, 3H), 2.07 (br. s, 3H), 1.35 (s, 9H).

tert-butyl ((2*S*,3*S*)-4-((cyclohexylmethyl)amino)-3-hydroxy-1-phenylbutan-2-yl)carbamate (5a)

The reaction of *tert*-butyl ((*S*)-1-((*R*)-oxiran-2-yl)-2-phenylethyl)carbamate (1.500 g, 5.70 mmol) with 3-cyclohexylamine (0.710 g, 6.27 mmol) in the presence of catalytic amount of pyridine in isopropanol (20 mL) was performed according to the **procedure A**. Purification: flash column chromatography (5% MeOH in DCM). Yield: 1.330 g (62.0%), TLC (DCM/MeOH, 9/1 *v/v*) R_f = 0.38, MW 376.54, formula: $C_{22}H_{36}N_2O_3$, MS *m/z* 377.4 ($M+H^+$), ^1H NMR (500 MHz, CHLOROFORM-*d*) δ ppm 7.17 - 7.33 (m, 5H), 4.99 (d, *J* = 9.17 Hz, 1H), 3.74 (q, *J* = 8.02 Hz, 1H), 3.64 (dd, *J* = 9.16, 3.44 Hz, 1H), 3.07 (br. s, 2H), 2.85 - 2.98 (m, 2H), 2.56 - 2.69 (m, 2H), 2.38 - 2.51 (m, 2H), 1.63 - 1.76 (m, 5H), 1.36 - 1.45 (m, 9H), 1.08 - 1.35 (m, 4H), 0.82 - 0.95 (m, 2H).

Procedure for the synthesis of compounds 4b, 11b and 16b (procedure B):

To the solution of the appropriate BOC-protected compound (1.0 equiv.) in DCM, TFA (5 mL/1 mmol substrate) was added dropwise. The reaction mixture was stirred at room temperature for 2 h. When the reaction was finished, the solvent and TFA were evaporated under reduced pressure, producing a residue that was then dissolved in an ammonia-water solution and extracted with DCM. The organic extracts were combined, dried over anhydrous Na_2SO_4, filtered and concentrated under a vacuum.

(2S,3S)-3-amino-1-((3-methoxybenzyl)amino)-4-phenylbutan-2-ol (3b)

The reaction of *tert*-butyl ((2S,3S)-3-hydroxy-4-((3-methoxybenzyl)amino)-1-phenylbutan-2-yl)carbamate (**3a**) (1.218 g, 3.04 mmol) with TFA (6 mL) in DCM (4 mL) was performed according to the **procedure B**. Purification: extraction with ammonia water solution (10 mL) and with DCM (3 x 10 mL). Yield: 0.780 g (85.4%), TLC (DCM/MeOH, 9/1 *v/v*) R_f = 0.34, MW 300.40, formula: $C_{18}H_{24}N_2O$, MS *m/z* 301.3 ($M+H^+$), ^1H NMR (500 MHz, CHLOROFORM-*d*) δ ppm 7.15 - 7.34 (m, 6H), 6.89 - 6.94 (m, 2H), 6.81 - 6.84 (m, 1H), 3.83 - 3.87 (m, 1H), 3.82 (s, 3H), 3.76 - 3.81 (m, 1H), 3.62 (ddd, *J* = 8.31, 4.87, 3.44 Hz, 1H), 3.12 - 3.17 (m, 1H), 2.84 - 2.93 (m, 2H), 2.78 (dd, *J* = 12.03, 8.59 Hz, 1H), 2.46 (dd, *J* = 13.46, 10.02 Hz, 1H), 2.09 (br. s, 4H).

(2S,3S)-3-amino-1-((cyclohexylmethyl)amino)-4-phenylbutan-2-ol (5b)

The reaction of *tert*-butyl ((2S,3S)-3-hydroxy-4-((cyclohexyl)amino)-1-phenylbutan-2-yl)carbamate (**5a**) (1.330 g, 3.53 mmol) with TFA (3 mL) in DCM (2 mL) was performed according to the **procedure B**. Purification: extraction with ammonia water solution (10 mL) and with DCM (3 x 10 mL). Yield: 0.965 g (85.4%), TLC (DCM/MeOH, 9/1 *v/v*) R_f = 0.25, MW 276.42, formula: $C_{17}H_{28}N_2O$, MS *m/z* 277.3 ($M+H^+$), ^1H NMR (500 MHz, CHLOROFORM-*d*) δ ppm 7.28 - 7.34 (m, 2H), 7.19 - 7.26 (m, 3H), 3.59 (dt, *J* = 7.30, 3.51 Hz, 1H), 2.90 - 2.97 (m, 3H), 2.65 - 2.89 (m, 4H), 2.52 - 2.64 (m, 3H), 2.45 (dd, *J* = 12.03, 6.87 Hz, 1H), 1.64 - 1.79 (m, 5H), 1.50 (dtt, *J* = 14.43, 7.32, 7.32, 3.51, 3.51 Hz, 1H), 1.10 - 1.30 (m, 3H), 0.86 - 0.97 (m, 2H).

Procedure for the synthesis of compounds 1–6 (procedure C):

To the solution of the appropriate primary amine (1.0 equiv.) in acetonitrile, 9-bromofluorene (1.2 equiv.) was added in the presence of K_2CO_3 (2 equiv.). The reaction mixture was stirred at room temperature for 2 h. When the total substrate consumption was observed by TLC, the reaction was finished, and the solvent was evaporated under reduced pressure. The residue was then dissolved in DCM and washed with three portions of water. The organic phase was dried over anhydrous Na_2SO_4, filtered, and concentrated under a vacuum. The resulting residue was purified by flash column chromatography using a mixture of DCM and MeOH.

(2*R*,3*S*)-3-((9*H*-fluoren-9-yl)amino)-1-(benzylamino)-4-phenylbutan-2-ol (1)

The reaction of (2*R*,3*S*)-3-amino-1-(benzylamino)-4-phenylbutan-2-ol (**1b**) (0.200 g, 0.74 mmol) with 9-bromofluorene (0.181 g, 0.74 mmol) in the presence of K_2CO_3 (0.205 g, 1.48) in acetonitrile (6 mL) was performed according to the **procedure C.** Purification: extraction with water (10 mL) and ethyl acetate (3 x 10 mL), than “flash” column chromatography (5% MeOH in DCM). Yield: 0.107 g (33.3%), TLC (10% MeOH in DCM) R_f = 0.38, MW 434.58, formula: $C_{30}H_{30}N_2O$, MS *m/z* 435.5 ($M+H^+$), ^1H NMR (500 MHz, CHLOROFORM-*d*) δ ppm 72.66 - 7.74 (m, 2H), 7.62 (d, *J* = 7.45 Hz, 1H), 7.27 - 7.41 (m, 8H), 7.13 - 7.25 (m, 5H), 6.98 - 7.13 (m, 2H), 4.96 (s, 1H), 3.62 - 3.90 (m, 2H), 3.59 (dt, *J* = 9.59, 4.65 Hz, 1H), 3.00 (dt, *J* = 9.17, 4.58 Hz, 1H), 2.80 - 2.95 (m, 2H), 2.73 (dd, *J* = 13.75, 4.58 Hz, 1H), 2.59 (br. s,

3H), 2.43 (dd, J = 13.46, 9.45 Hz, 1H); ^{13}C NMR (126 MHz, CHLOROFORM-d) δ ppm 144.38, 144.00, 141.29, 140.98, 139.23, 138.85, 129.37, 129.16, 128.69, 128.59, 128.48, 128.41, 127.65, 127.57, 127.22, 126.40, 125.70, 125.33, 120.33, 120.00, 69.977, 66.08, 56.17, 55.51, 53.39, 38.92.

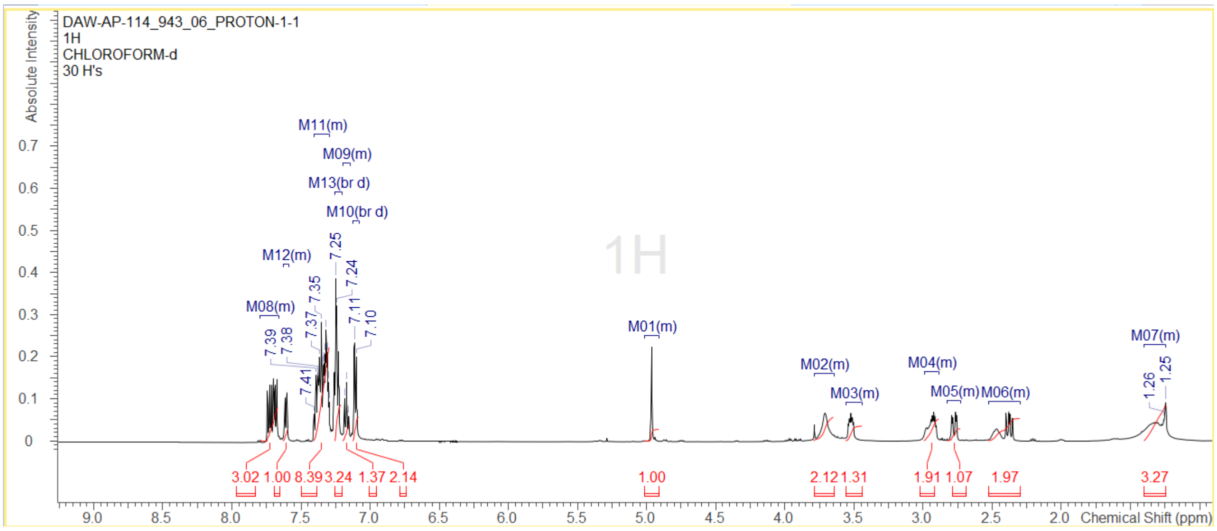

Figure S1. ^{1}H NMR (300 MHz, CHLOROFORM-d) spectrum of compound **1**.

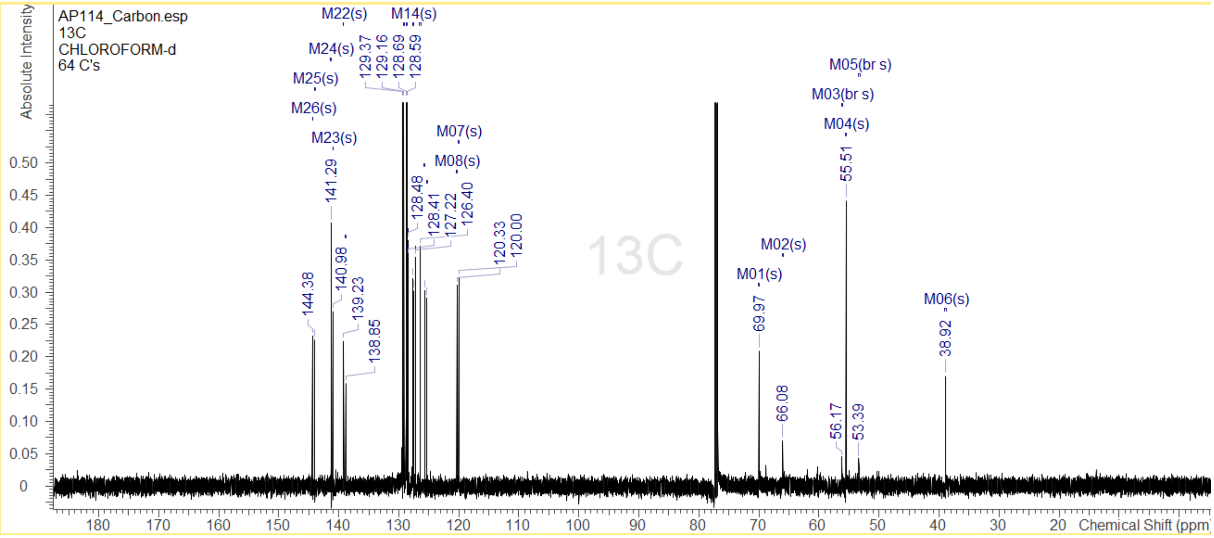

Figure S2. ^{13}C NMR (75 MHz, CHLOROFORM-d) spectrum of compound **1**.

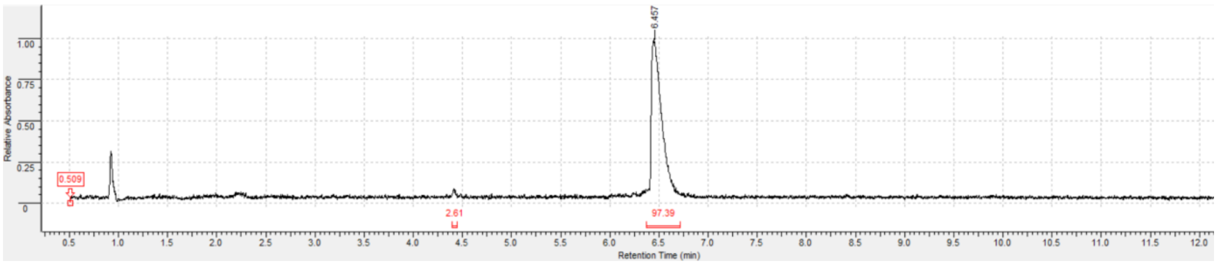

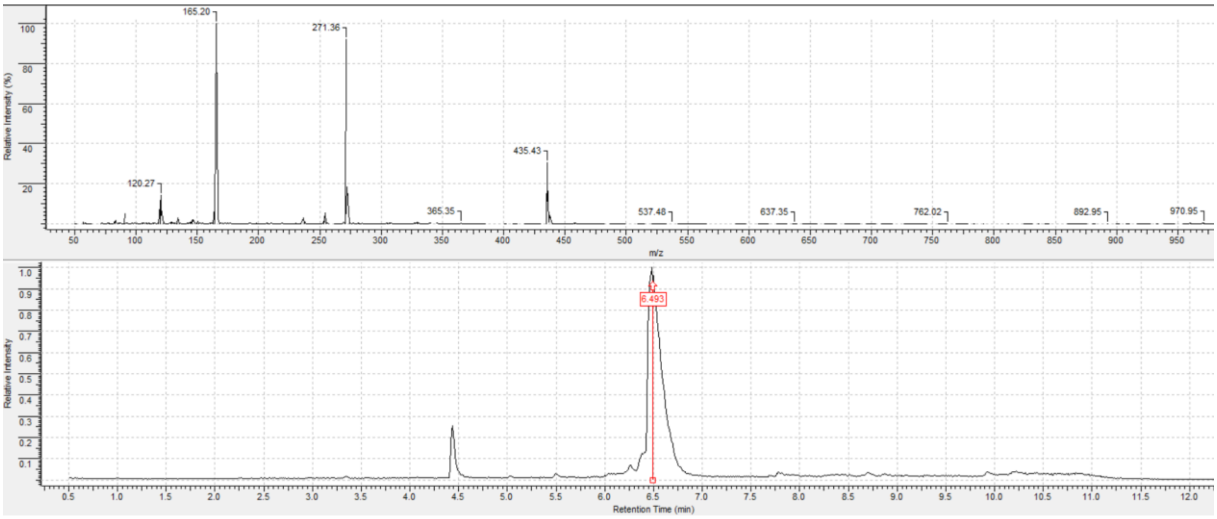

Figure S3. MS spectra of compound **1** ([M+H]$^{+}$ 435.43, purity 97.4%)**.**

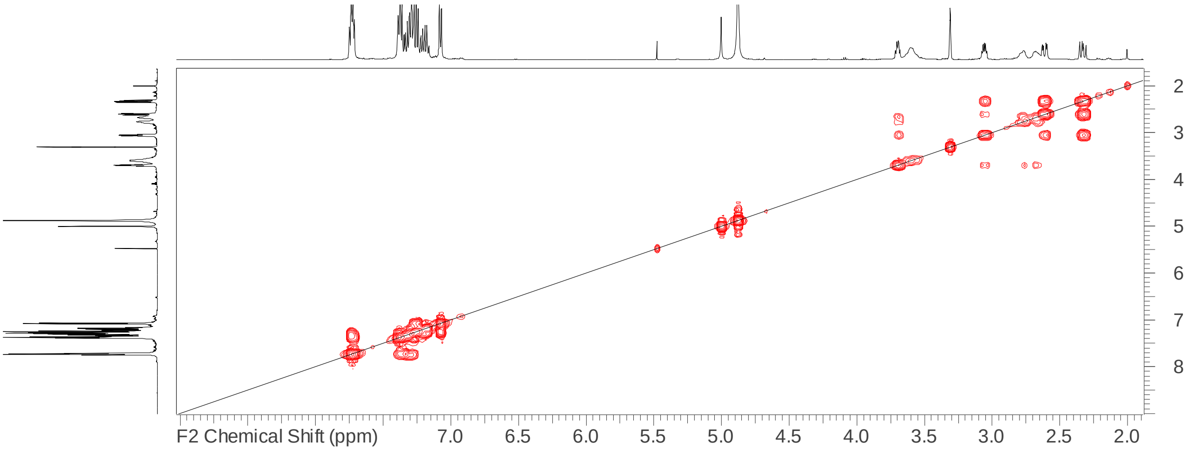

Figure S4. 2D COSY NMR (methanol-d4) spectrum of compound **1**.

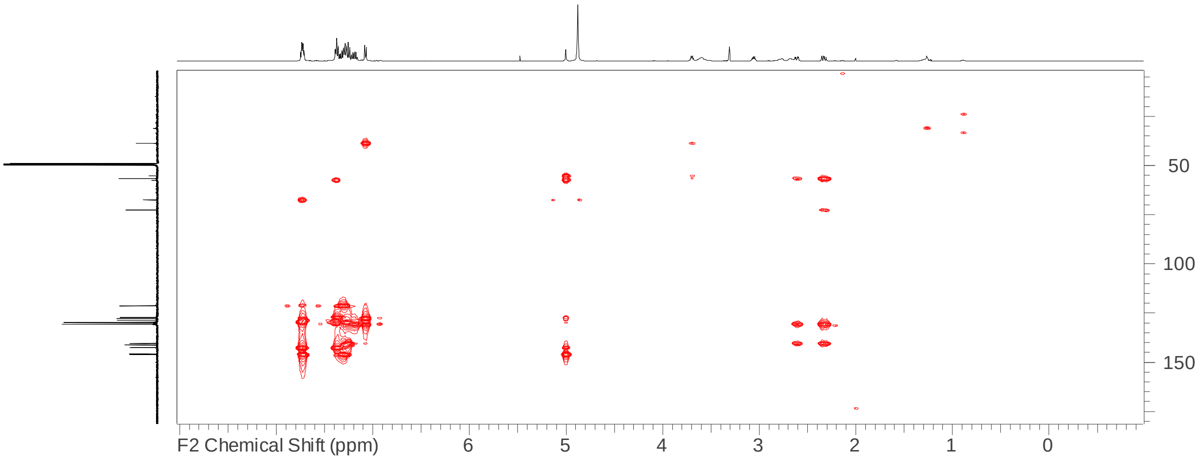

Figure S5. 2D HMBC NMR (methanol-d4) spectrum of compound **1**.

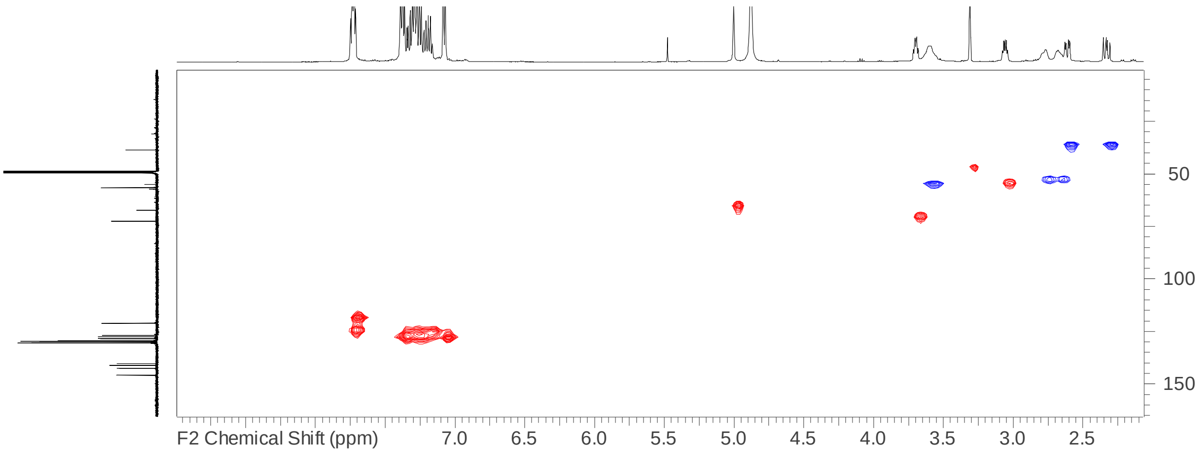

Figure S6. 2D HSQC NMR (methanol-d4) spectrum of compound **1**.

(2*S*,3*S*)-3-((9*H*-fluoren-9-yl)amino)-1-((2-methoxybenzyl)amino)-4-phenylbutan-2-ol (2)

The reaction of (2*S*,3*S*)-3-amino-1-((2-methoxybenzyl)amino)-4-phenylbutan-2-ol (**2b**) (0.150 g, 0.50 mmol) with 9-bromofluorene (0.122 g, 0.50 mmol) in the presence of K_2CO_3 (0.138 g, 1.00 mmol) in acetonitrile (4 mL) was performed according to the **procedure C.** Purification: extraction with water (10 mL) and ethyl acetate (3 x 10 mL), then "flash" column chromatography (5% MeOH in DCM). Yield: 0.086 g (37.0%), TLC (10% MeOH in DCM) R_f = 0.31, MW 464.61, formula: $C_{31}H_{32}N_2O_2$, MS *m/z* 465.4 ($M+H^+$), ^{1}H NMR (300 MHz, CHLOROFORM-*d*) δ ppm 7.64 - 7.74 (m, 3H), 7.57 (d, *J* = 7.03 Hz, 1H), 7.08 - 7.42 (m, 11H), 6.88 - 6.98 (m, 2H), 4.91 (s, 1H), 4.00 (br. s, 2H), 3.91 (s, 3H), 3.47 - 3.56 (m, 1H), 2.64 - 2.82 (m, 2H), 2.28 - 2.61 (m, 3H), 1.46 (br. s, 3H); ^{13}C NMR (75 MHz, CHLOROFORM-*d*) δ ppm 158.16, 144.73, 144.47, 141.04, 140.78, 139.24, 131.36, 129.24, 128.92, 128.39, 128.03, 127.23, 126.91, 126.13, 125.58, 125.46, 120.54, 120.04, 119.70, 110.64, 69.48, 65.65, 55.28, 54.49, 53.62, 51.86, 40.86.

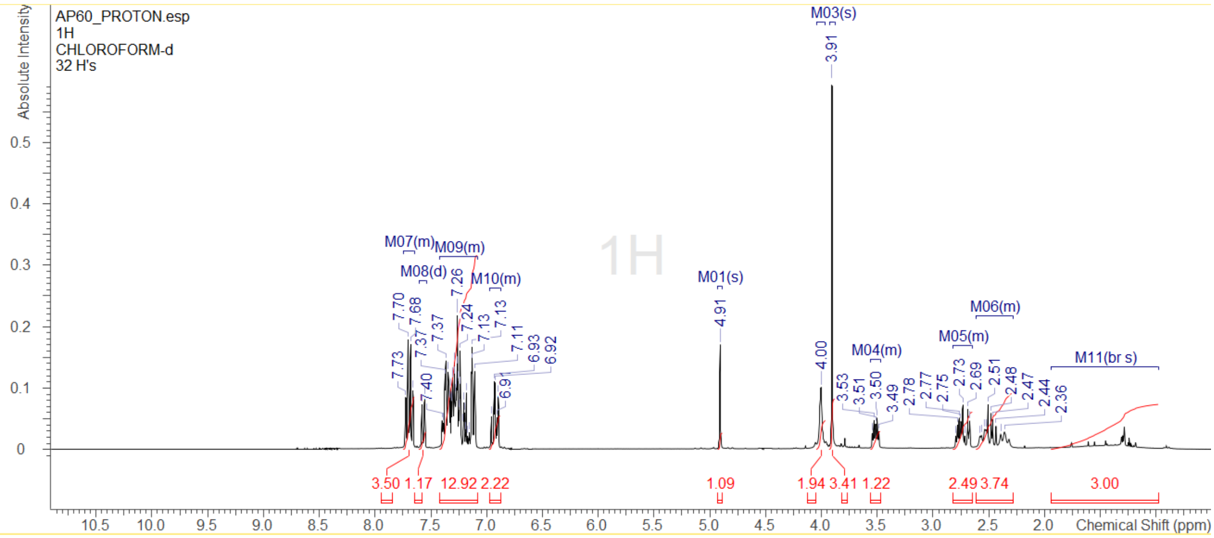

Figure S7. ^{1}H NMR (300 MHz, CHLOROFORM-*d*) spectrum of compound **2**.

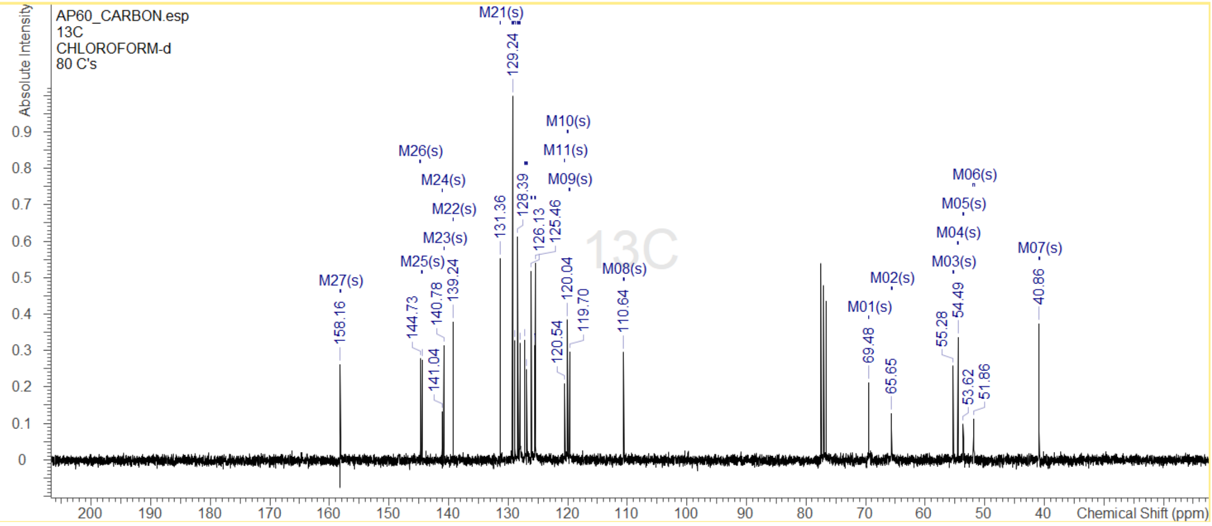

Figure S8. ^{13}C NMR (75 MHz, CHLOROFORM-*d*) spectrum of compound **2**.

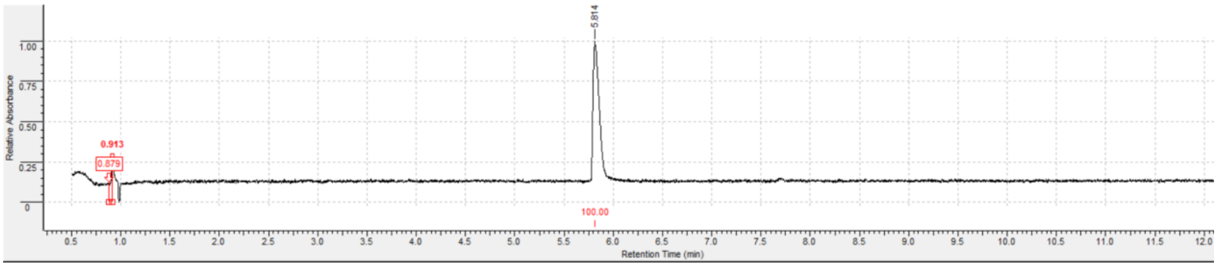

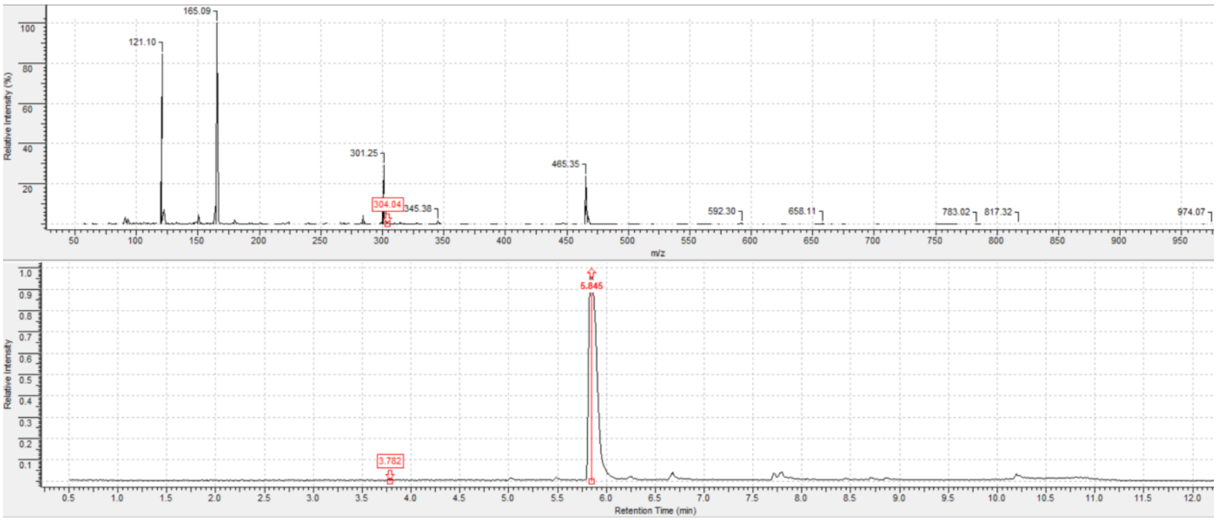

Figure S9. MS spectra of compound **2** ([M+H]$^+$ 465.35, purity 100%).

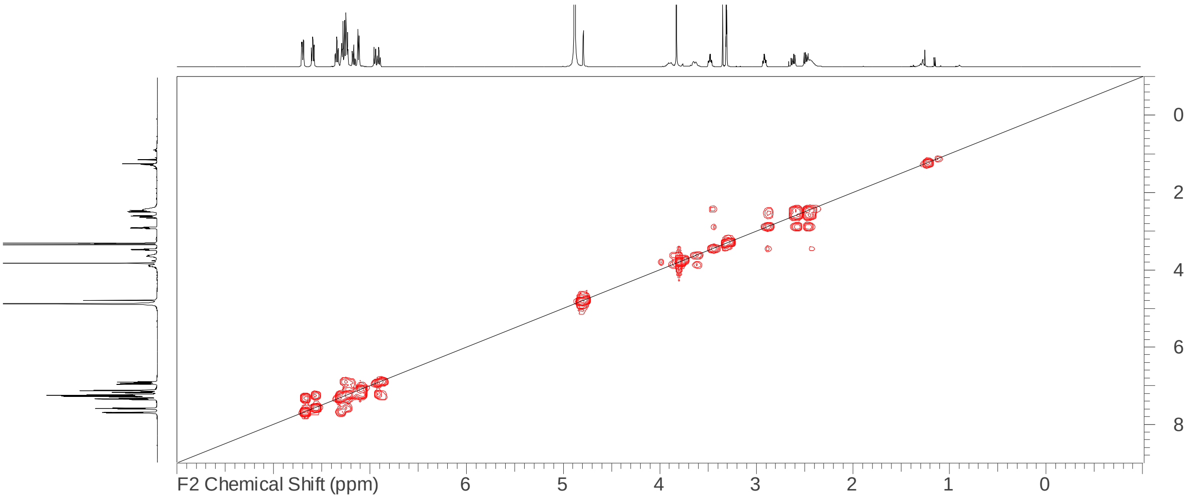

Figure S10. 2D COSY NMR (methanol-d4) spectrum of compound **2**.

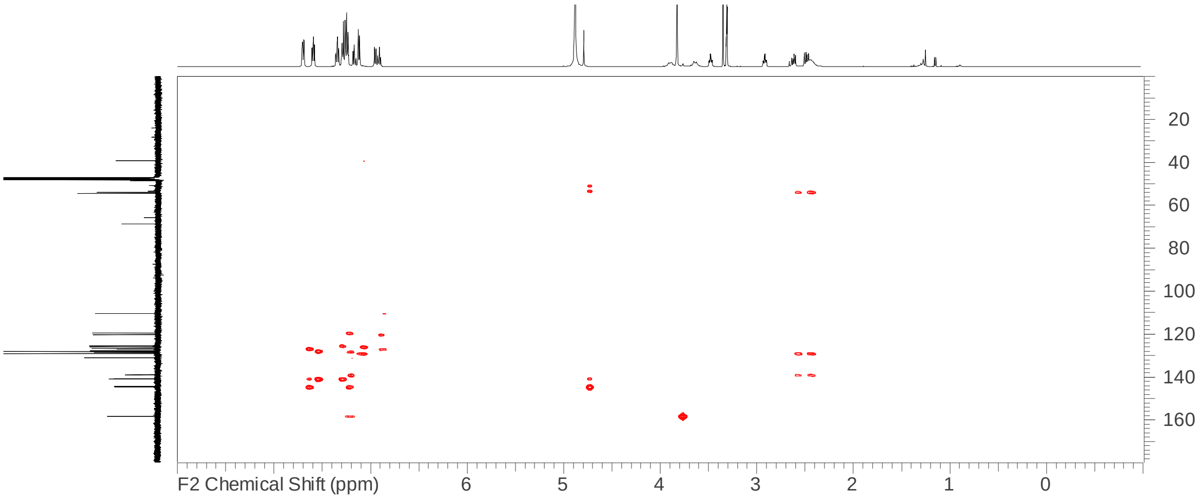

Figure S11. 2D HMBC NMR (methanol-d4) spectrum of compound **2**.

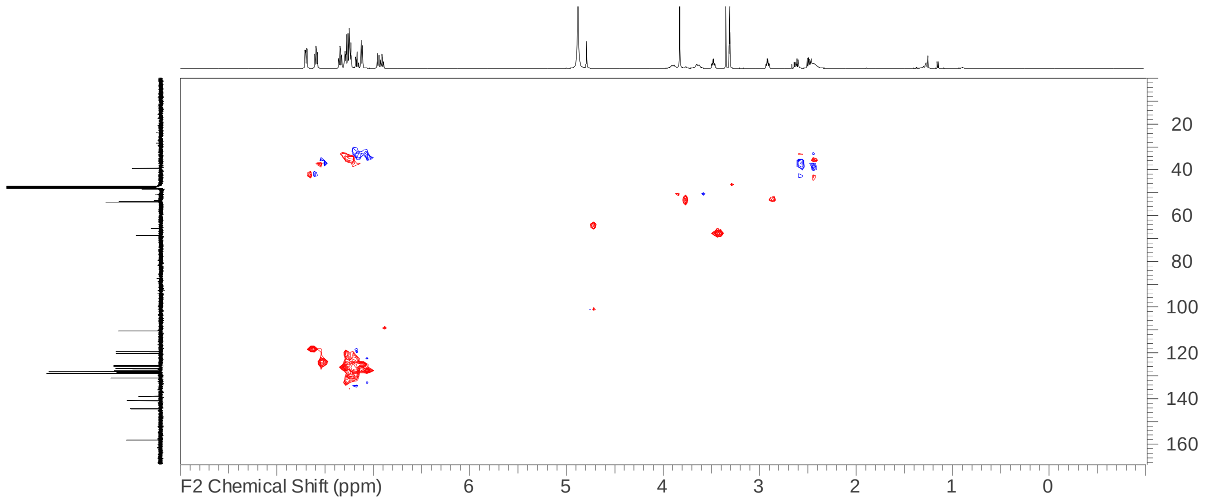

Figure S12. 2D HSQC NMR (methanol-d4) spectrum of compound **2**.

(2*S*,3*S*)-3-((9*H*-fluoren-9-yl)amino)-1-((3-methoxybenzyl)amino)-4-phenylbutan-2-ol (3)

The reaction of (2*S*,3*S*)-3-amino-1-((3-methoxybenzyl)amino)-4-phenylbutan-2-ol (**3b**) (0.120 g, 0.40 mmol) with 9-bromofluorene (0.098 g, 0.40 mmol) in the presence of K_2CO_3 (0.120 g, 1.00 mmol) in acetonitrile (3 mL) was performed according to the **procedure C.** Purification: extraction with water (10 mL) and ethyl acetate (3 x 10 mL), then "flash" column chromatography (5% MeOH in DCM). Yield: 0.086 g (37.0%), TLC (10% MeOH in DCM) R_f = 0.33, MW 464.61, formula: $C_{31}H_{32}N_2O_2$, MS *m/z* 465.4 (M+H$^+$), ^{1}H NMR (300 MHz, CHLOROFORM-*d*) δ ppm 7.61 - 7.73 (m, 4H), 7.11 - 7.43 (m, 10H), 6.90 - 7.00 (m, 2H), 6.79 (dd, *J* = 8.21, 2.34 Hz, 1H), 4.96 (s, 1H), 3.80 (s, 3H), 3.66 (s, 2H), 3.45 - 3.57 (m, 1H), 2.90 - 3.00 (m, 1H), 2.57 - 2.76 (m, 3H), 2.44 - 2.55 (m, 1H), 1.82 (br. s, 3H); ^{13}C NMR (75 MHz, CHLOROFORM-*d*) δ ppm 159.76, 144.33, 144.26, 141.11, 139.10, 129.51, 129.21, 128.48, 128.31, 127.35, 127.12, 126.28, 125.46, 121.27, 120.10, 114.60, 112.70, 69.51, 66.21, 56.44, 55.21, 54.54, 53.94, 40.91.

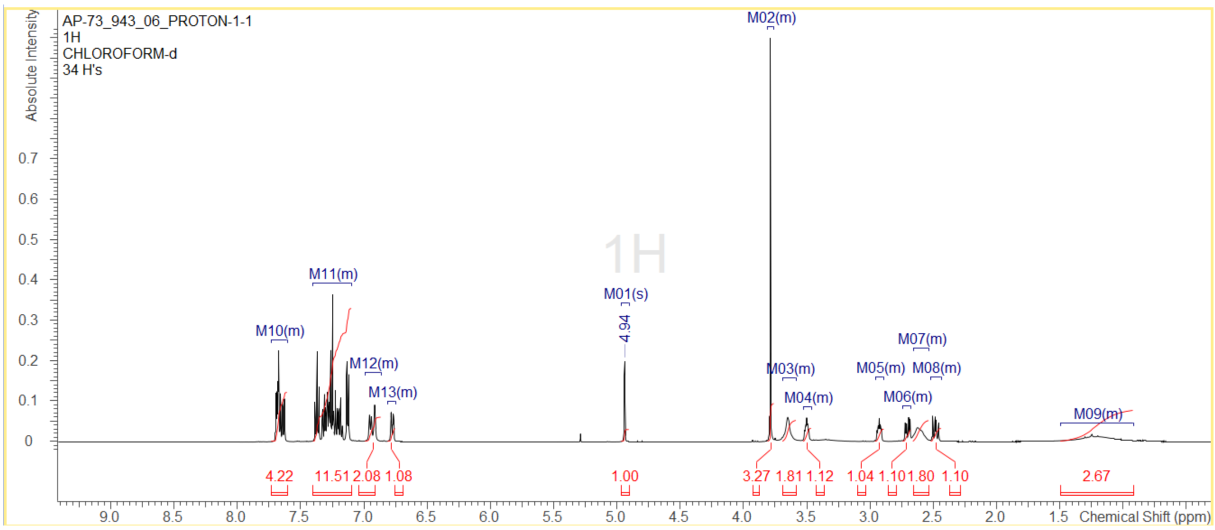

Figure S13. ^{1}H NMR (300 MHz, CHLOROFORM-*d*) spectrum of compound **3**.

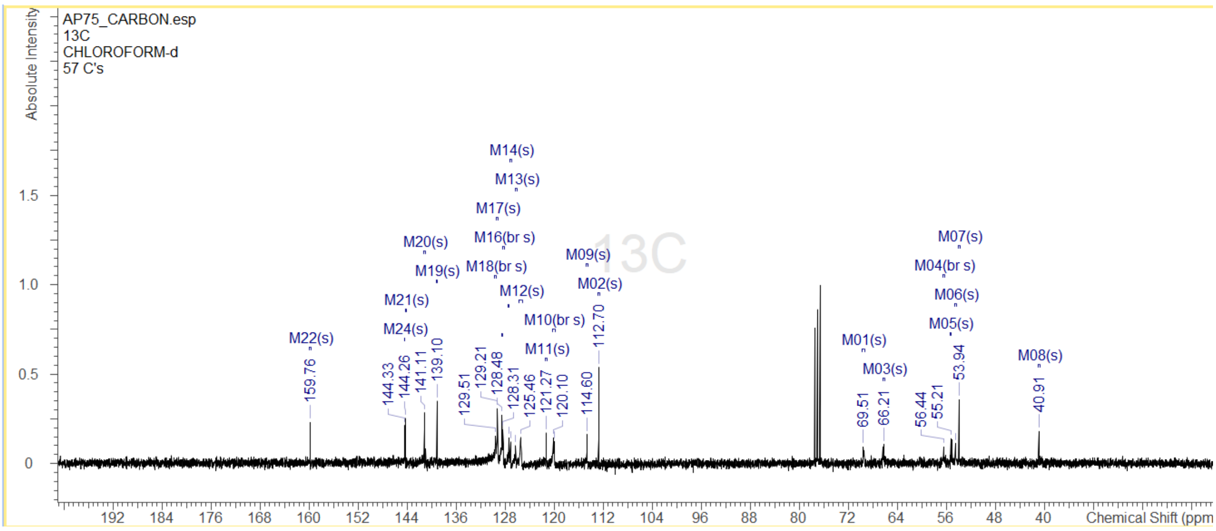

Figure S14. ^{13}C NMR (75 MHz, CHLOROFORM-*d*) spectrum of compound **3**.

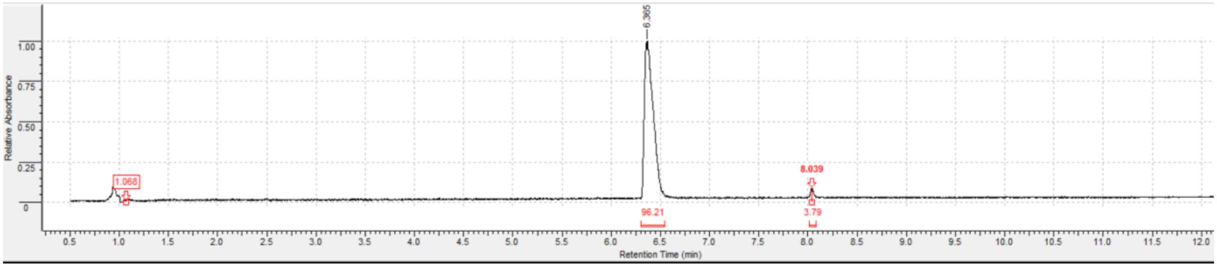

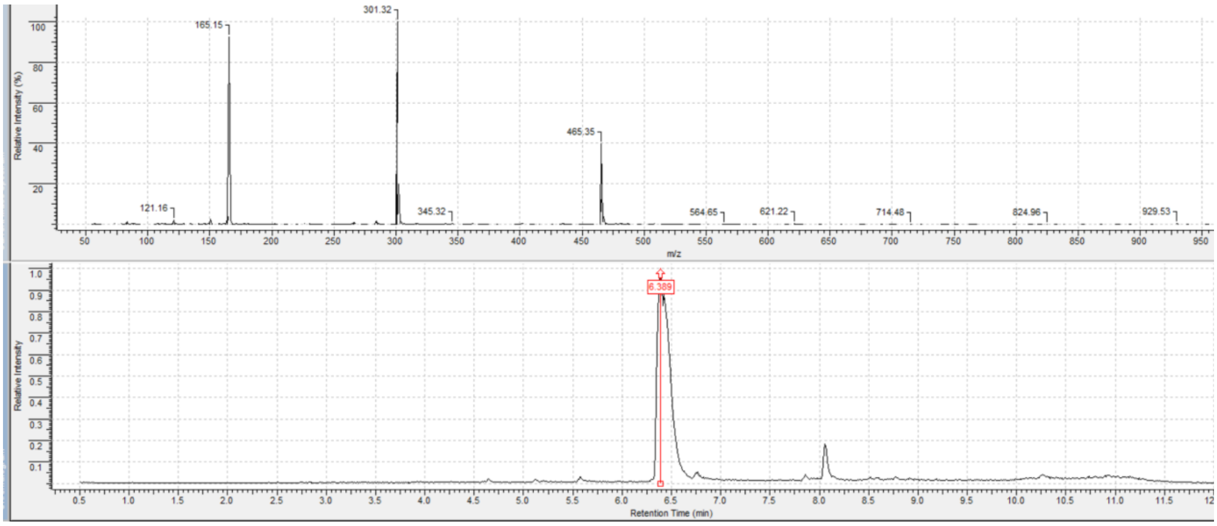

Figure S15. MS spectra of compound **3** ([M+H]$^{+}$ 465.35, purity 96.2%).

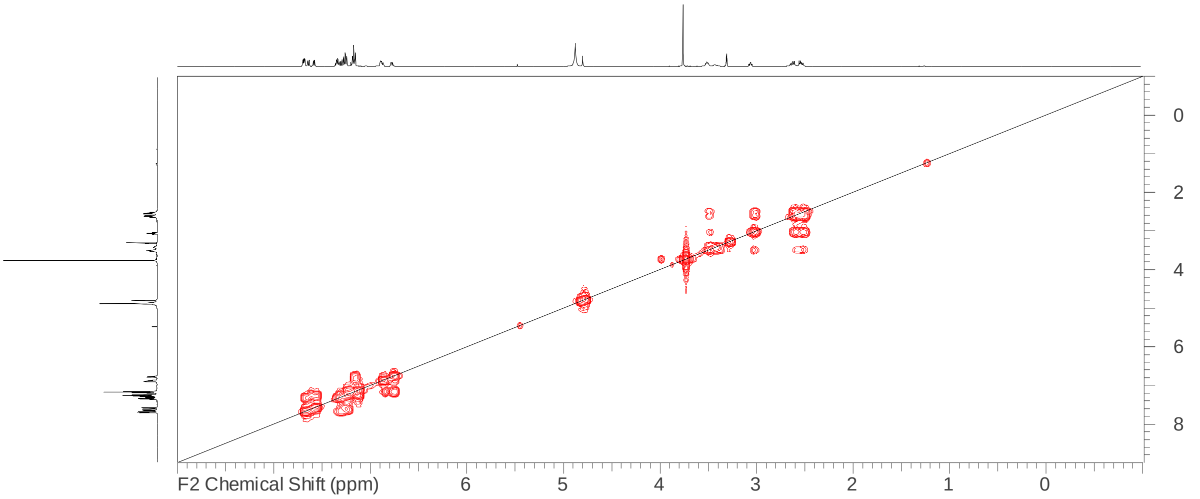

Figure S16. 2D COSY NMR (methanol-d4) spectrum of compound 3.

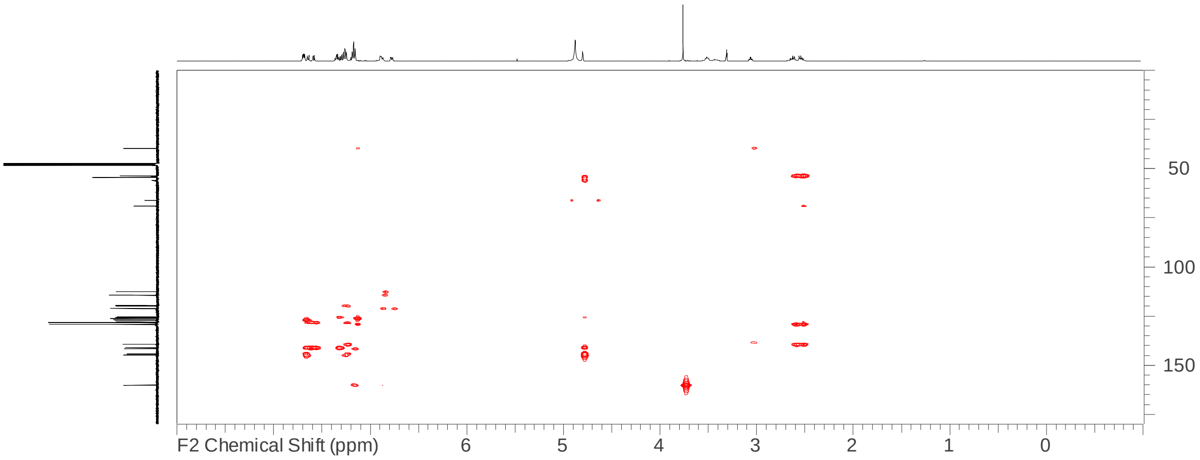

Figure S17. 2D HMBC NMR (methanol-d4) spectrum of compound 3.

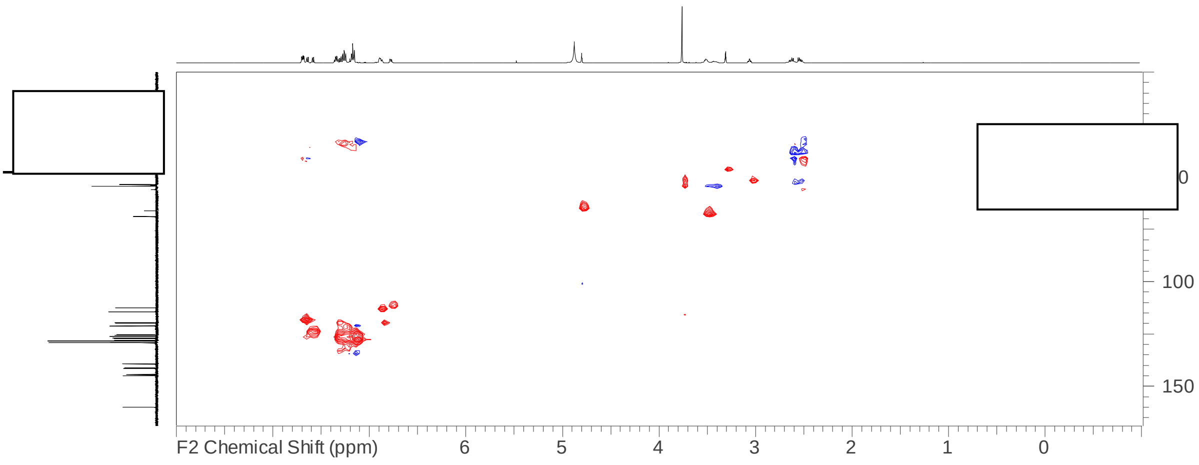

Figure S18. 2D HSQC NMR (methanol-d4) spectrum of compound 3.

(2*R*,3*S*)-3-((9*H*-fluoren-9-yl)amino)-1-((3-(*tert*-butyl)benzyl)amino)-4-phenylbutan-2-ol (4)

The reaction of (2*R*,3*S*)-3-amino-1-((3-(*tert*-butyl)benzyl)amino)-4-phenylbutan-2-ol (**4b**) (0.100 g, 0.31 mmol) with 9-bromofluorene (0.075 g, 0.31 mmol) in the presence of K_2CO_3 (0.085 g, 0.61 mmol) in acetonitrile (4 mL) was performed according to the **procedure C.** Purification: extraction with water (10 mL) and ethyl acetate (3 x 10 mL), then “flash” column chromatography (5% MeOH in DCM). Yield: 0.044 g (29.3%), TLC (10% MeOH in DCM) R_f = 0.40, MW 490.69, formula: $C_{34}H_{38}N_2O$, MS *m/z* 490.69 ($M+H^+$), ^{1}H NMR (500 MHz, CHLOROFORM-*d*) δ ppm 7.68 - 7.78 (m, 3H), 7.63 (d, *J* = 7.45 Hz, 1H), 7.23 - 7.44 (m, 9H), 7.16 - 7.21 (m, 2H), 7.11 - 7.15 (m, 2H), 4.98 (s, 1H), 3.75 (br. s, 2H), 3.55 (m, 1H), 2.92 - 3.02 (m, 2H), 2.76 - 2.83 (m, 1H), 2.50 (br. s, 1H), 2.40 (dd, *J* = 13.46, 9.45 Hz, 1H), 1.33 (s, 9H), 1.31 (br. s, 3H); ^{13}C NMR (126 MHz, CHLOROFORM-*d*) δ ppm 151.55, 144.54, 144.09, 141.31, 140.98, 139.12, 138.89, 129.38, 128.56, 128.46, 128.39, 127.64, 127.18, 126.34, 126.18, 126.02, 125.71, 125.29, 124.47, 120.35, 120.00, 70.22, 66.10, 56.51, 55.54, 53.54, 39.46, 34.76, 31.50.

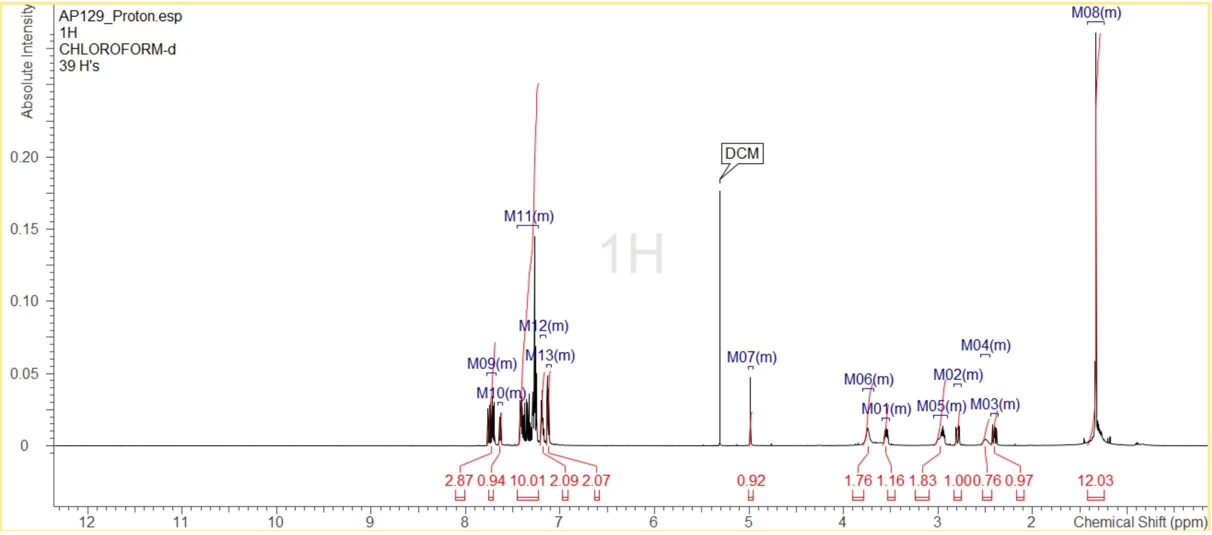

Figure S19. ^{1}H NMR (300 MHz, CHLOROFORM-*d*) spectrum of compound **4**.

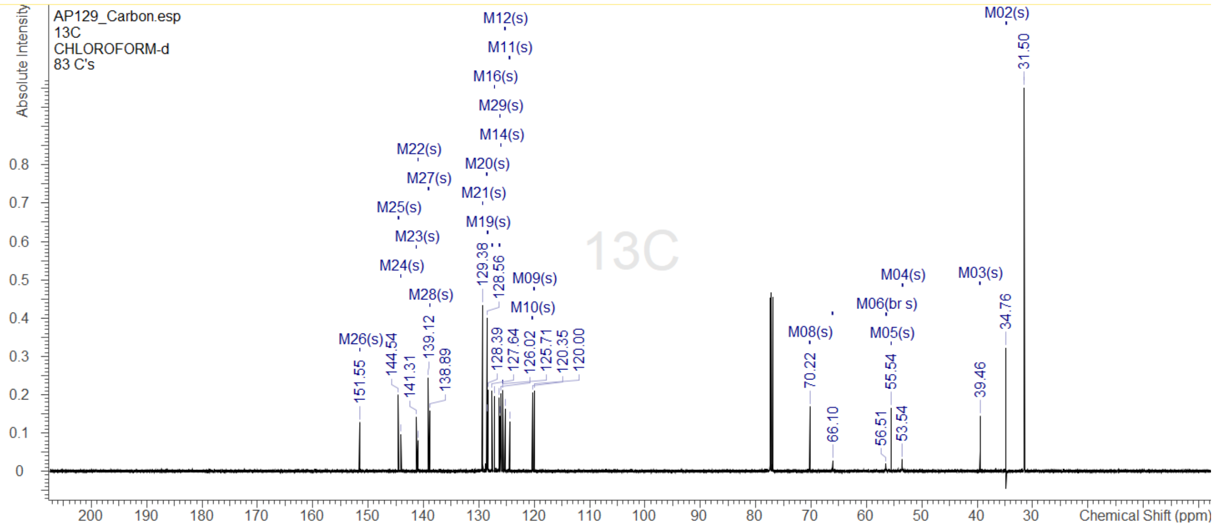

Figure S20. ^{13}C NMR (75 MHz, CHLOROFORM-*d*) spectrum of compound **4**.

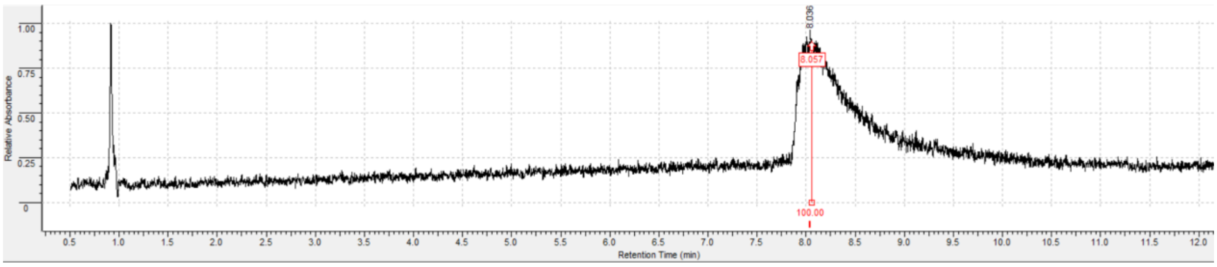

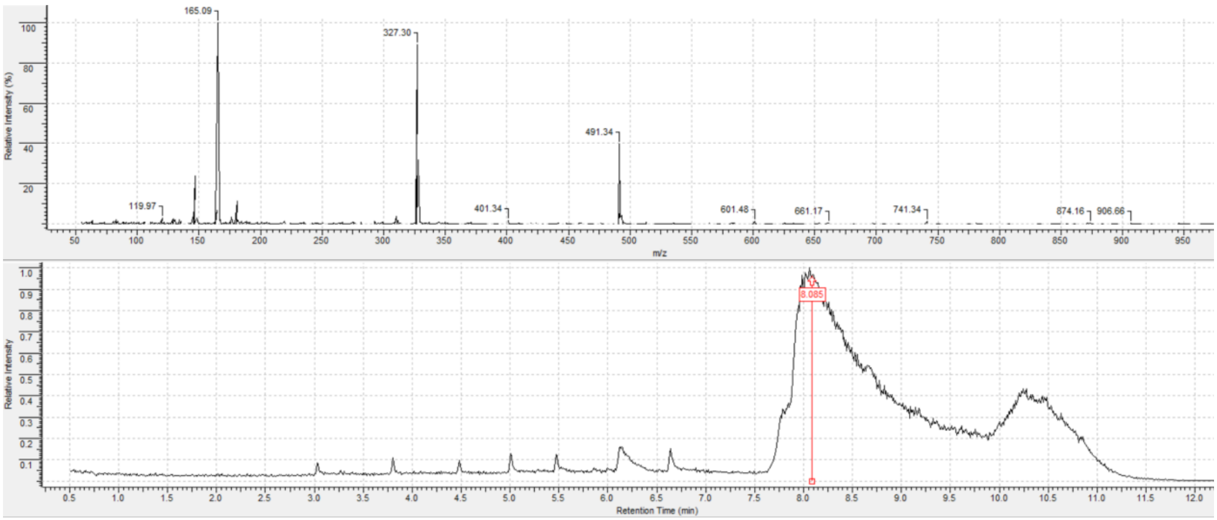

Figure S21. MS spectra of compound **4** ([M+H]$^+$ 491.34, purity 100%).

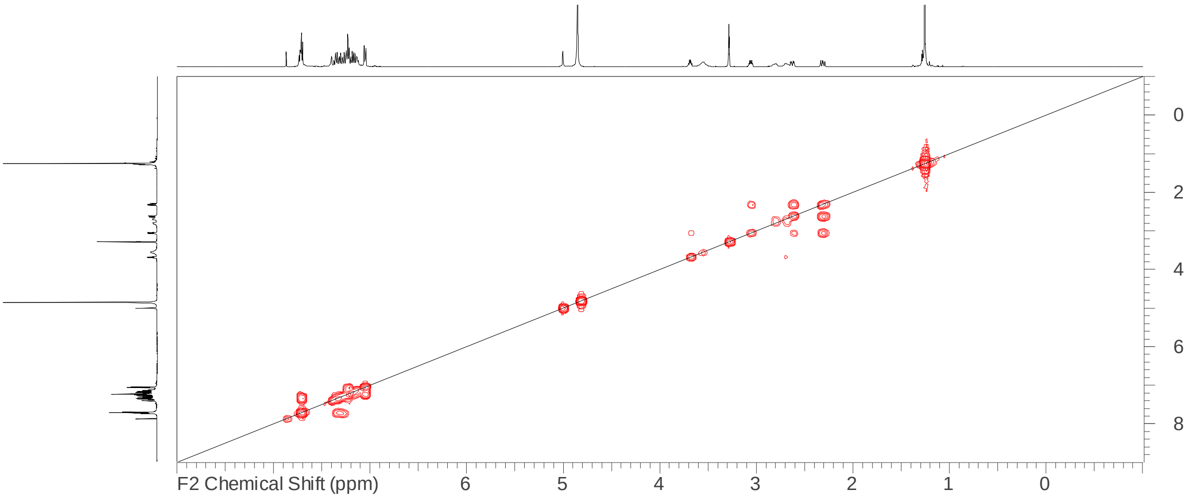

Figure S22. 2D COSY NMR (methanol-d4) spectrum of compound **4**.

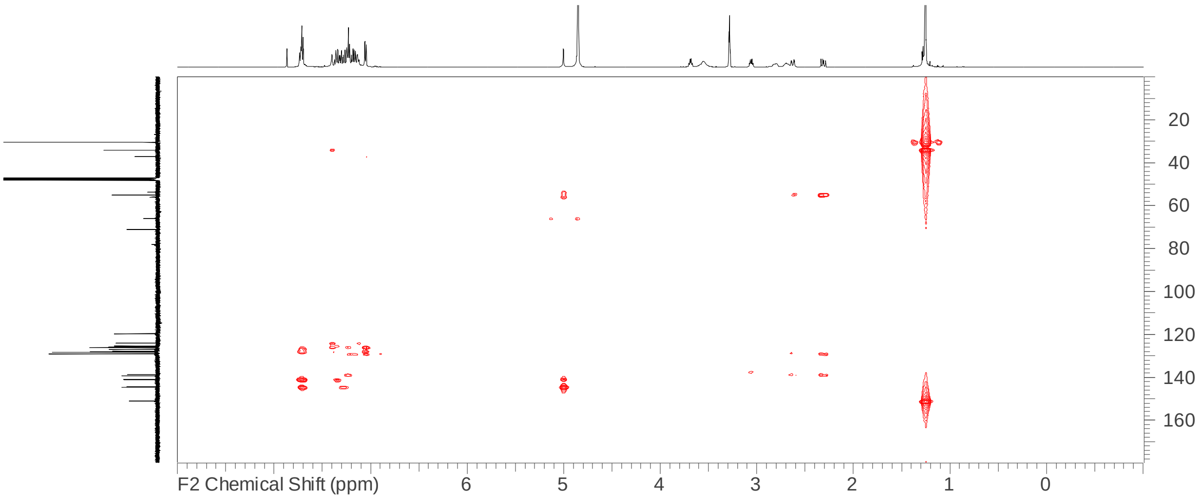

Figure S23. 2D HMBC NMR (methanol-d4) spectrum of compound **4**.

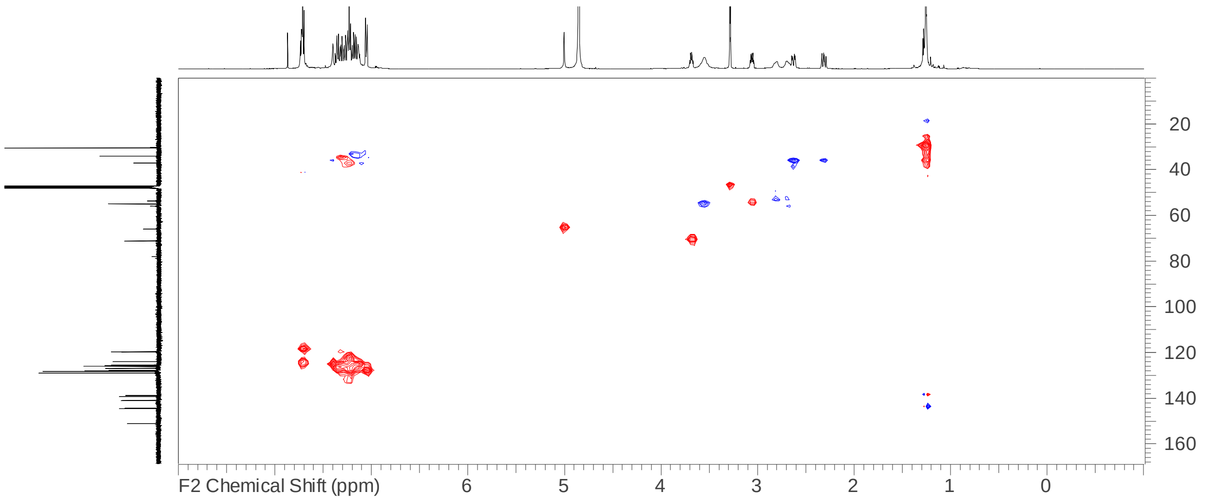

Figure S24. 2D HSQC NMR (methanol-d4) spectrum of compound **4**.

(2*S*,3*S*)-3-((9*H*-fluoren-9-yl)amino)-1-((cyclohexylmethyl)amino)-4-phenylbutan-2-ol (5)

The reaction of (2*S*,3*S*)-3-amino-1-((cyclohexylmethyl)amino)-4-phenylbutan-2-ol (**5b**) (0.150 g, 0.54 mmol) with 9-bromofluorene (0.133 g, 0.54 mmol) in the presence of K_2CO_3 (0.150 g, 1.09 mmol) in acetonitrile (5 mL) was performed according to the **procedure C.** Purification: extraction with water (10 mL) and ethyl acetate (3 x 10 mL), then “flash” column chromatography (5% MeOH in DCM). Yield: 0.119 g (49.8%), TLC (10% MeOH in DCM) R_f = 0.34, MW 440.63, formula: $C_{30}H_{36}N_2O$, MS *m/z* 441.3 ($M+H^+$), ^{1}H NMR (500 MHz, CHLOROFORM-*d*) δ ppm 7.70 (t, *J* = 7.16 Hz, 2H), 7.63 (dd, *J* = 7.45, 1.15 Hz, 1H), 7.59 (d, *J* = 7.45 Hz, 1H), 7.38 (td, *J* = 7.30, 2.58 Hz, 2H), 7.25 - 7.34 (m, 4H), 7.13 - 7.23 (m, 3H), 4.96 (s, 1H), 3.52 (br. s, 1H), 2.81 – 2.87 (m, 1H), 2.77 (dd, *J* = 13.75, 4.58 Hz, 1H), 2.64 (br. s, 1H), 2.53 (dd, *J* = 13.17, 9.17 Hz, 2H), 2.37 (br. s, 2H), 1.95 (d, *J* = 11.46 Hz, 1H), 1.55 - 1.86 (m, 5H), 1.02 - 1.48 (m, 6H), 0.71 – 0.92 (m, 2H); ^{13}C NMR (126 MHz, CHLOROFORM-*d*) δ ppm 144.52, 144.28, 141.15, 140.88, 139.22, 129.36, 128.54, 128.32, 128.28, 127.42, 127.01, 126.33, 125.57, 125.42, 120.22, 119.93, 69.57, 66.71, 54.51, 53.53, 41.10, 36.44, 31.84, 31.61, 31.03, 26.80, 26.18, 26.06.

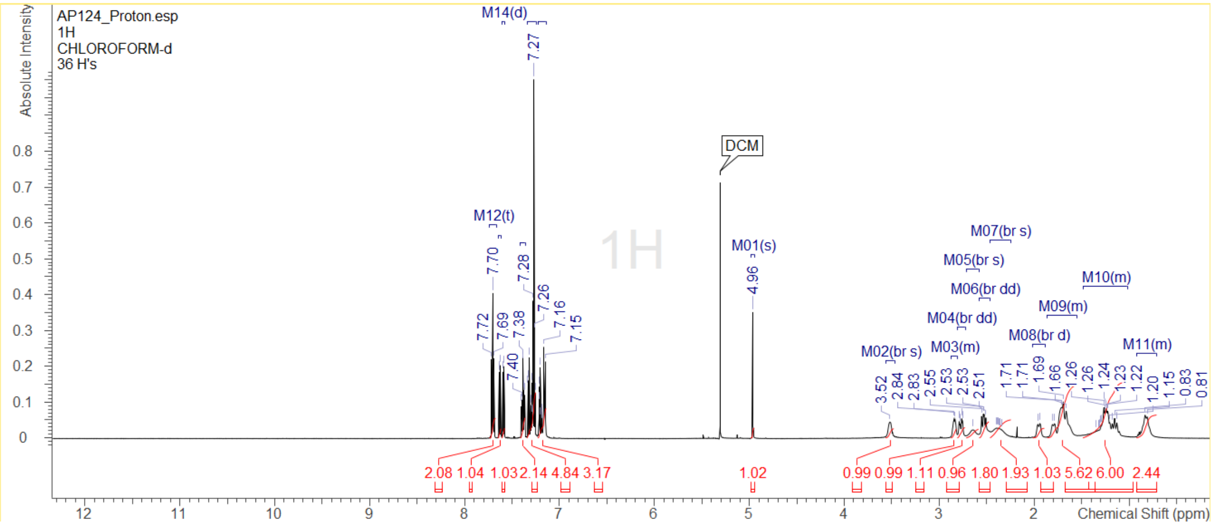

Figure S25. ^{1}H NMR (300 MHz, CHLOROFORM-*d*) spectrum of compound **5**.

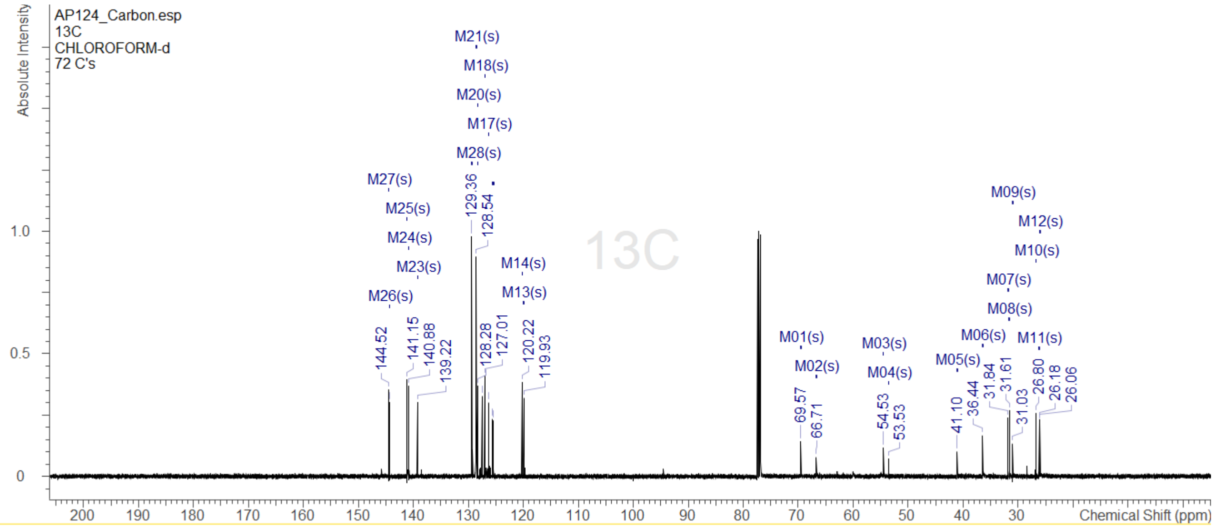

Figure S26. ^{13}C NMR (75 MHz, CHLOROFORM-*d*) spectrum of compound **5**.

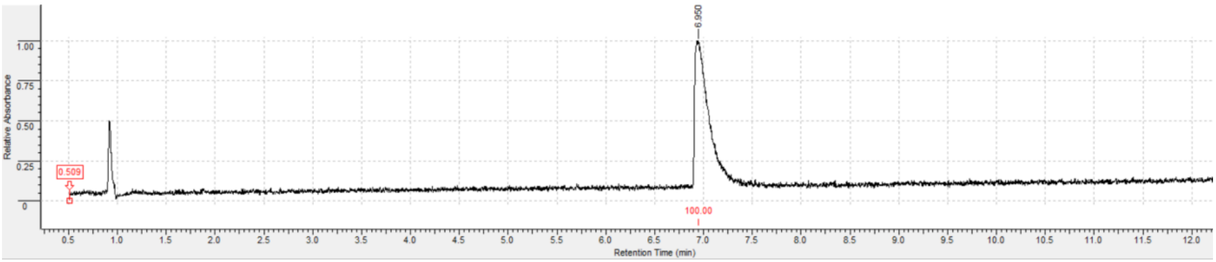

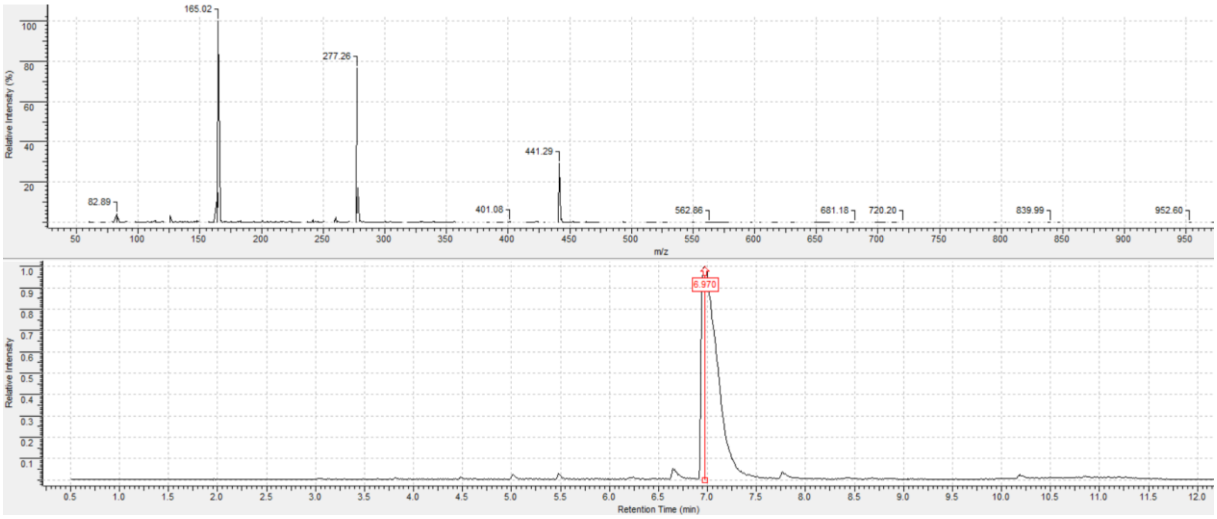

Figure S27. MS spectra of compound **5** ([M+H]$^+$ 441.29, purity 100%)**.**

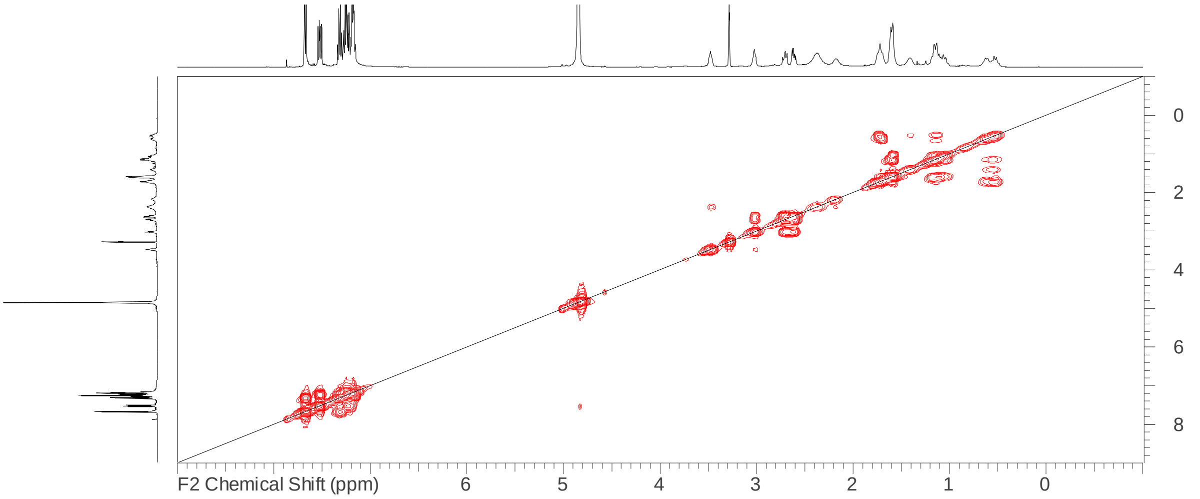

Figure S28. 2D COSY NMR (methanol-d4) spectrum of compound **5**.

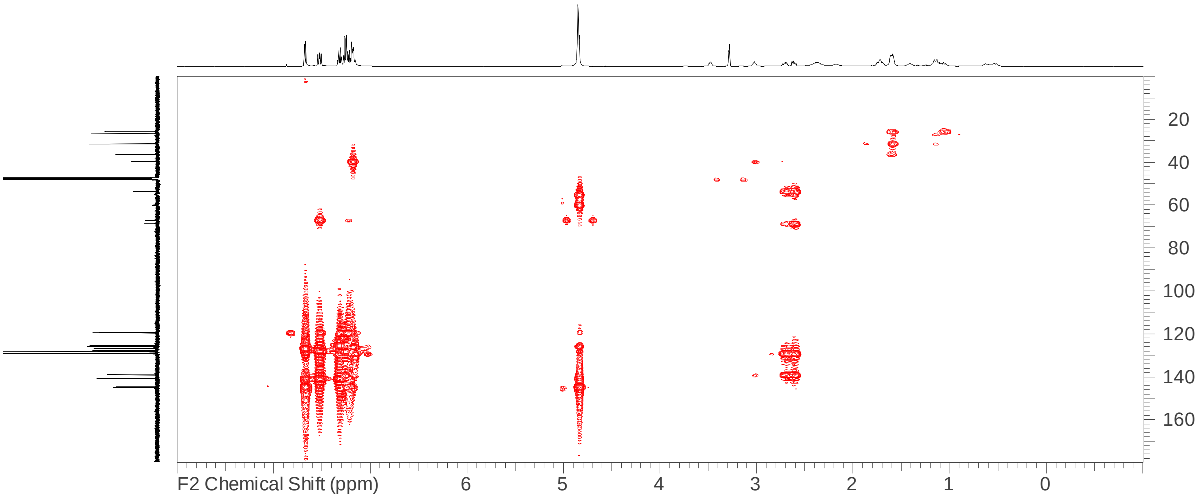

Figure S29. 2D HMBC NMR (methanol-d4) spectrum of compound **5**.

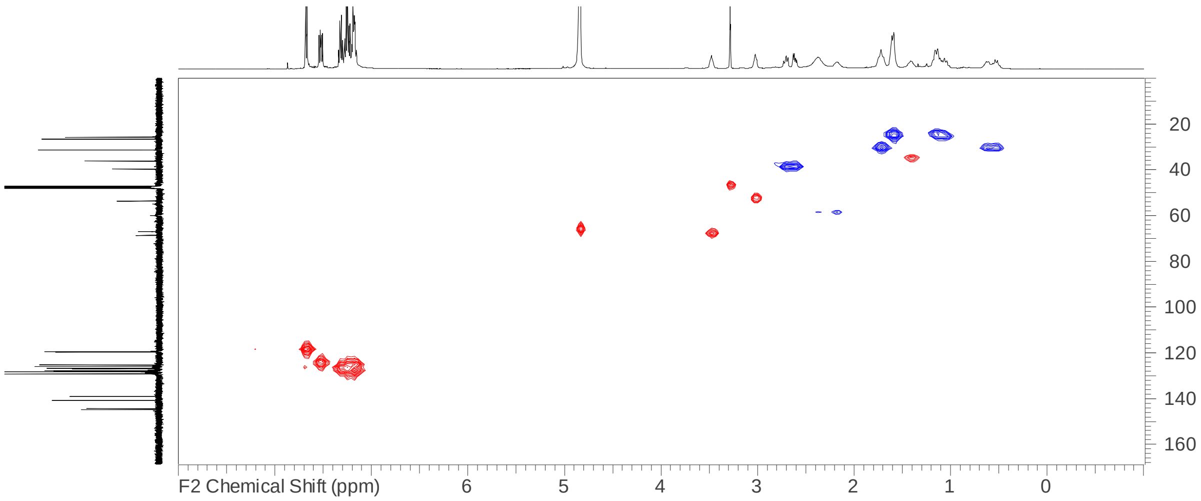

Figure S30. 2D HSQC NMR (methanol-d4) spectrum of compound **5**.

(2*R*,3*S*)-3-((9*H*-fluoren-9-yl)amino)-1-((cyclopropylmethyl)amino)-4-phenylbutan-2-ol (31) (6)

The reaction of (2*R*,3*S*)-3-amino-1-((cyclopropylmethyl)amino)-4-phenylbutan-2-ol (**6b**) (0.150 g, 0.64 mmol) with 9-bromofluorene (0.157 g, 0.64 mmol) in the presence of K_2CO_3 (0.177 g, 1.28 mmol) in acetonitrile (5 mL) was performed according to the **procedure C.** Purification: extraction with water (10 mL) and ethyl acetate (3 x 10 mL), then "flash" column chromatography (5% MeOH in DCM). Yield: 0.060 g (23.6%), TLC (10% MeOH in DCM) R_f = 0.40, MW 398.55, formula: $C_{27}H_{30}N_2O$, MS *m/z* 399.2 ($M+H^+$), ^{1}H NMR (500 MHz, CHLOROFORM-*d*) δ ppm 7.71 (t, *J* = 7.45 Hz, 2H), 7.65 (dd, *J* = 16.90, 7.16 Hz, 2H), 7.40 (t, *J* = 7.40 Hz, 2H), 7.34 (td, *J* = 7.40, 1.15 Hz, 1H), 7.25 - 7.32 (m, 3H), 7.16 - 7.23 (m, 3H), 5.14 (s, 1H), 3.63 (m, 1H), 2.97 - 3.11 (m, 2H), 2.91 (dd, *J* = 13.46, 3.72 Hz, 1H), 2.60 - 2.71 (m, 1H), 2.56 (dd, *J* = 12.89, 6.59 Hz, 1H), 2.48 (dd, *J* = 13.75, 9.74 Hz, 1H), 2.28 (dd, *J* = 12.03, 6.30 Hz, 1H), 1.40 (br. s, 3H), 0.83 - 0.97 (m, 1H), 0.48 - 0.56 (m, 1H), 0.44 (tt, *J* = 8.38, 4.51 Hz, 1H), -0.04 - 0.12 (m, 2H); ^{13}C NMR (126 MHz, CHLOROFORM-*d*) δ ppm 144.61, 144.24, 141.07, 140.86, 139.06, 129.40, 128.61, 128.36, 128.31, 127.52, 127.13, 126.40, 125.54, 125.37, 120.23, 119.93, 69.99, 67.27, 56.59, 55.74, 54.12, 39.44, 10.92, 5.06, 3.72.

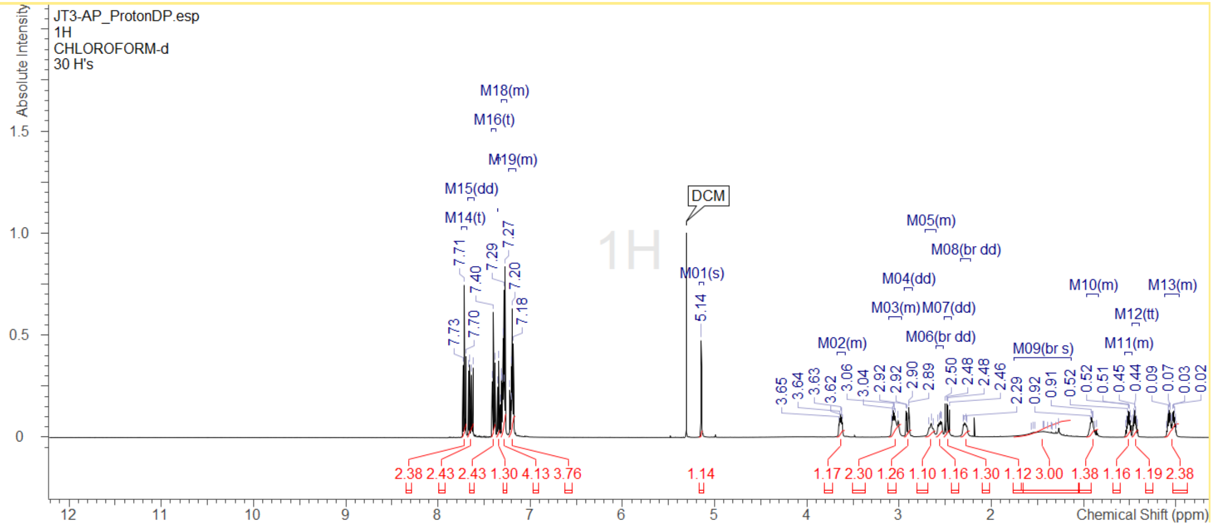

Figure S31. ^{1}H NMR (300 MHz, CHLOROFORM-*d*) spectrum of compound **6**.

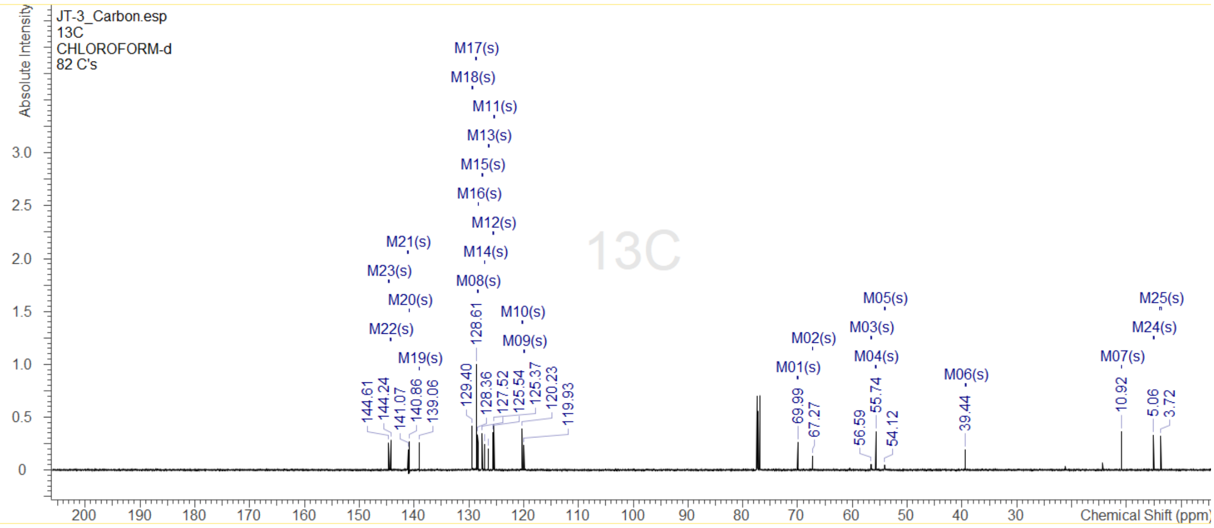

Figure S32. ^{13}C NMR (75 MHz, CHLOROFORM-*d*) spectrum of compound **6**.

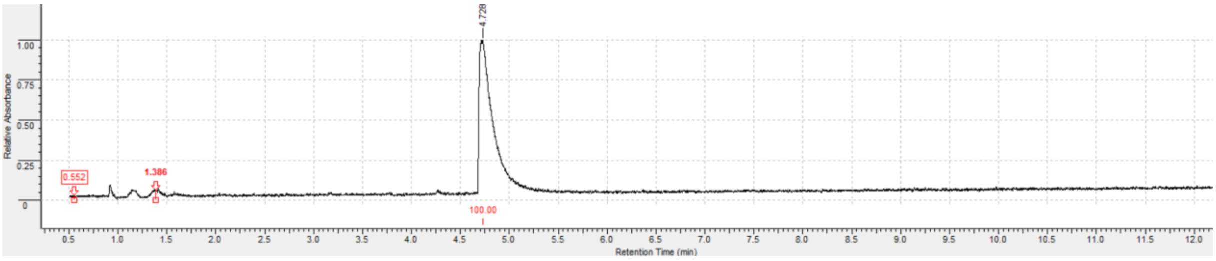

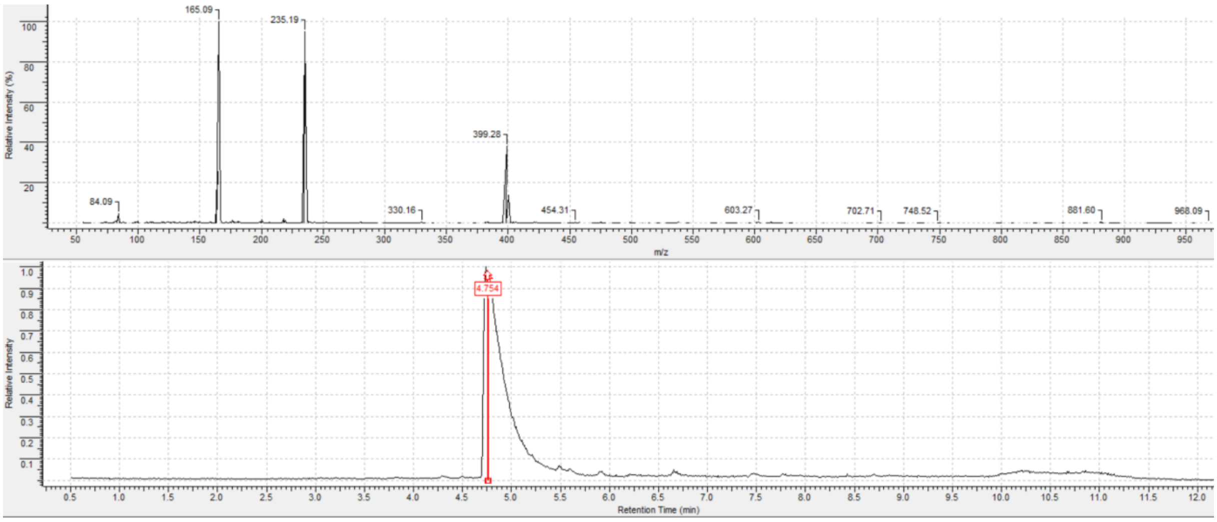

Figure S33. MS spectra of compound **6** ([M+H]$^+$ 399.28, purity 100%).

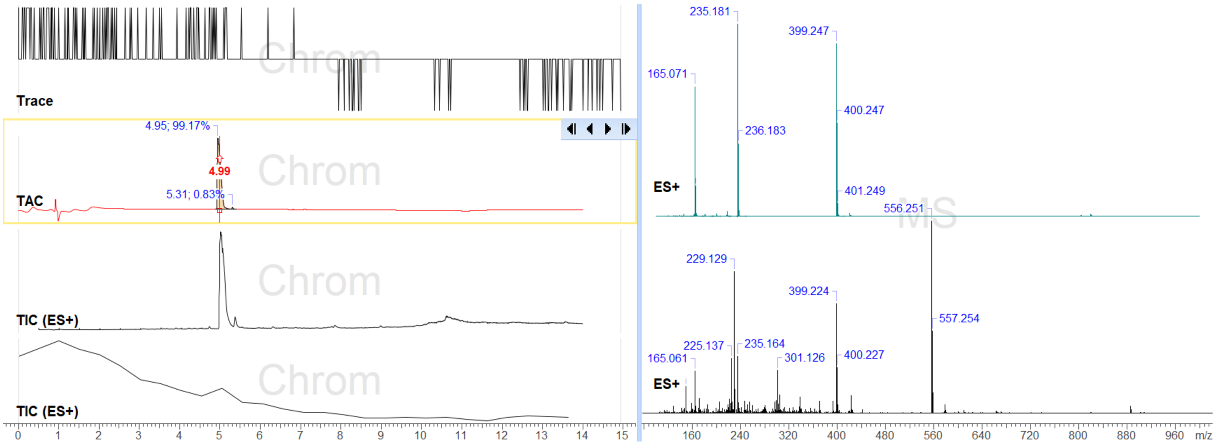

Figure S34. HRMS analysis of compound 6 ([M+H]$^+$ 399.247, purity 99.17%)

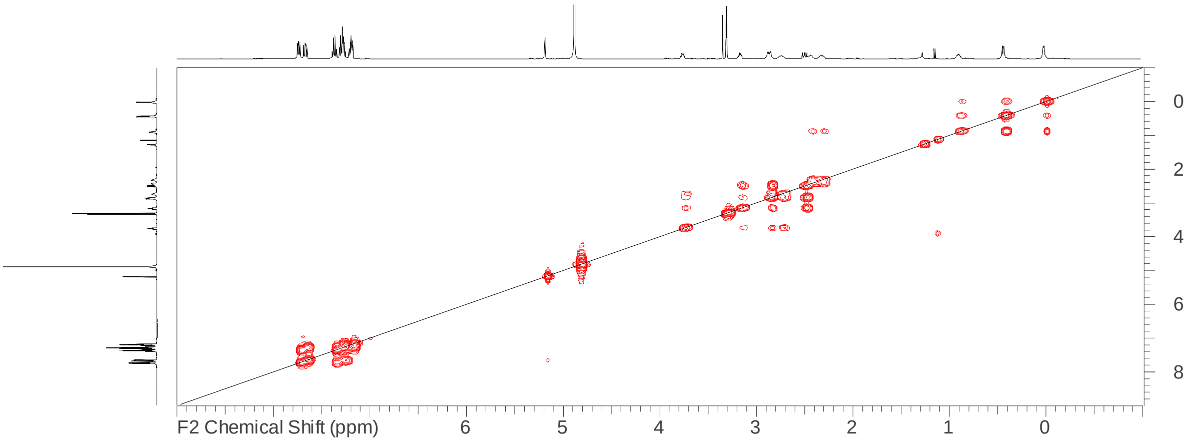

Figure S35. 2D COSY NMR spectrum of compound **6**.

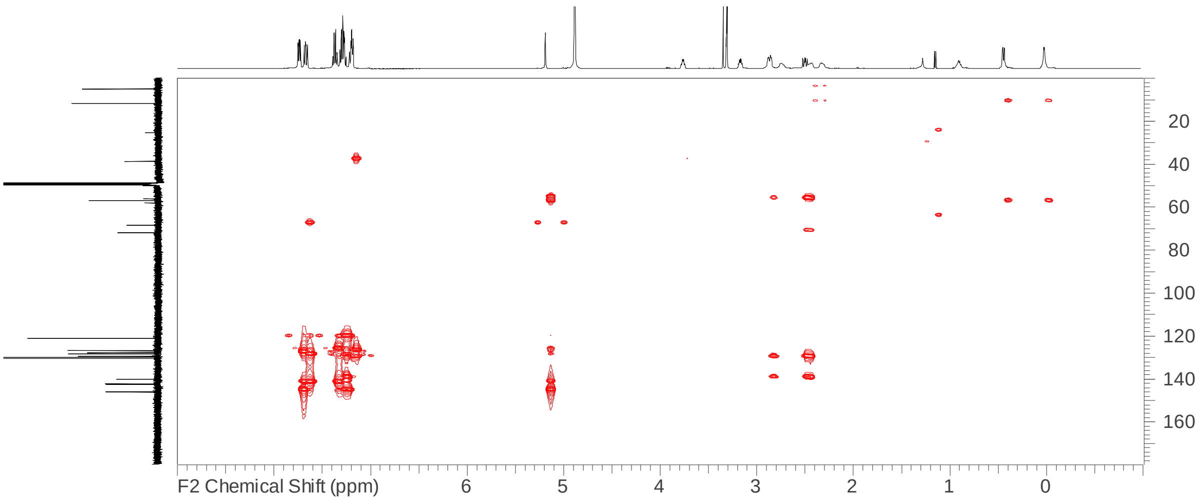

Figure S36. 2D HMBC NMR spectrum of compound **6**.

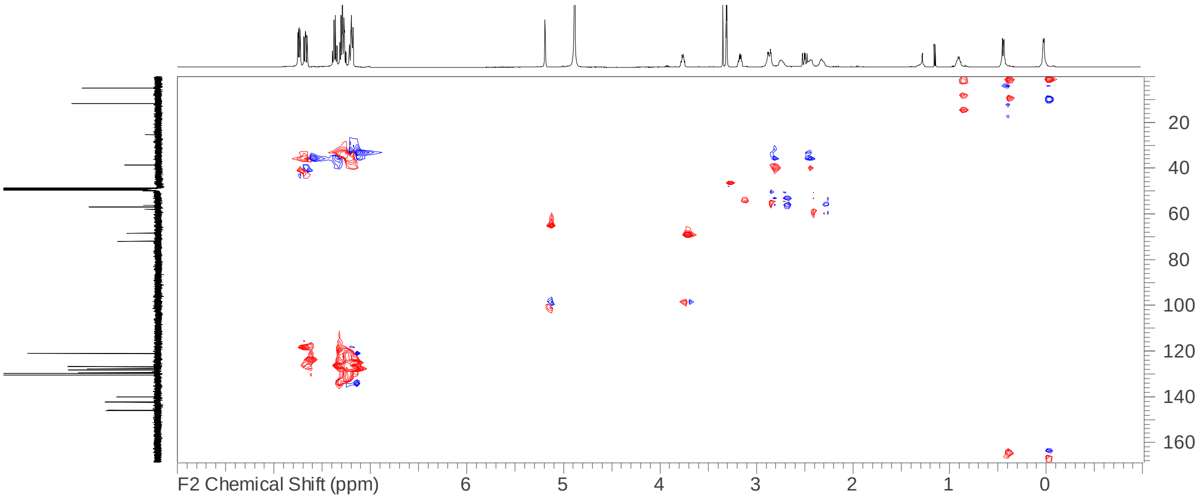

Figure S37. 2D HSQC NMR spectrum of compound **6**.

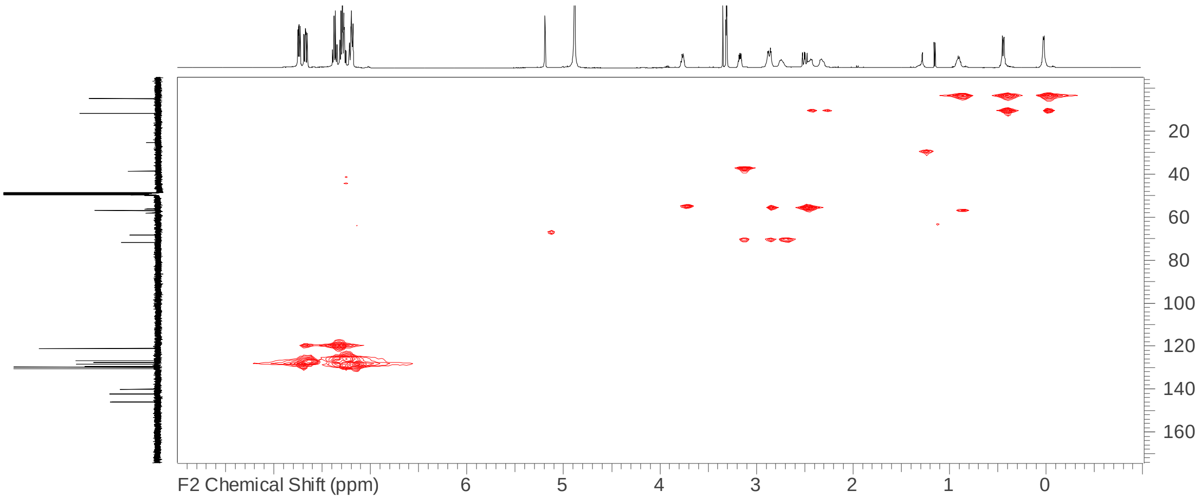

Figure S38. 2D H2BC NMR spectrum of compound **6**.

Molecular modelling

Methodology

The protein and ligand preparation steps were carried out using modules of Maestro 2023-1 software (Maestro, Schrödinger, LLC, New York, NY, 2023). The model of the BuChE was imported from Protein Data Bank (7AMZ). Protein structure was prepared using the Protein Preparation Wizard module. During preparation, all water molecules with less than 2 H-bonds to non-waters beyond 5 Å from ligand were deleted, het states were set with Epik at pH 7.4, H-bond assignment with PROPKA at pH 7.4, and restrained minimization of the complex with OPLS4 force field was performed. Receptor Grid for molecular docking centered on RNZ ligand position and size scaled for docking ligands of size similar to reference was prepared with all recognised hydroxyl and thiol groups set as rotatable.

All ligands were prepared with the LigPrep module. Charges were calculated using Epik at pH 7.4. No additional tautomers or stereoisomers were generated.

Molecular docking was performed with Glide (Glide, Schrödinger, LLC, New York, NY, 2024) module applying extra precision (XP) settings collecting docking score for top pose per ligand. Final binding poses were additionally refined and re-scored with Prime MM-GBSA using the VSGB solvation model and OPLS4 force field.

The selected BuChE complexes with investigated ligands underwent 100 ns MD simulations. All MD simulations were performed using the Desmond GPU package (Desmond Molecular Dynamics System, D. E. Shaw Research, New York, NY, 2023. Maestro-Desmond Interoperability Tools, Schrödinger, New York, NY, 2023). Systems for the simulations were prepared using the System Builder module. Complexes were placed in an orthorhombic cell, with size adjusted to a necessary minimum and the TIP4P solvent model set. The 0.15 M of NaCl and an appropriate number of counterions to maintain charge neutrality were added to the system. After initial model relaxation, 20 ns simulations were run in the NPT ensemble at 310.15 K and 1.01325 bar. Trajectories were saved at 20 ps intervals.

The final binding modes were visualized using PyMOL (PyMOL Molecular Graphics System, Schrödinger, LLC)

Description

We performed the computational evaluation of **compound I** tricyclic derivatives – fluorene, carbazole, phenothiazine, and dibenzazepine – each linked to the molecule's core through amine or amide groups (**Table S1**).

Table S1. Assessment of binding of **Compound I** analogues in the active site of BuChE using molecular modeling methods.

R	Glide Score (XP)	MM-GBSA dG Bind	R	Glide Score (XP)	MM-GBSA dG Bind
		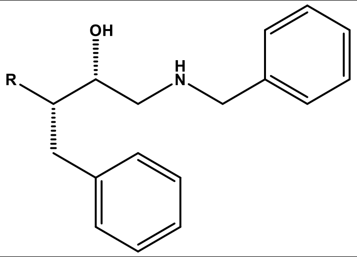			
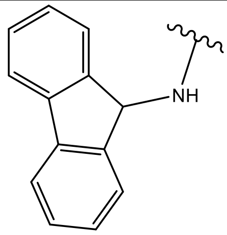	*-12.516*	*-80.44*	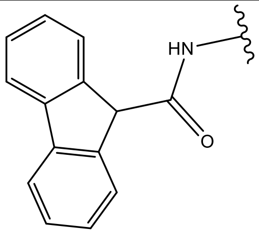	*-11.853*	*-67.07*
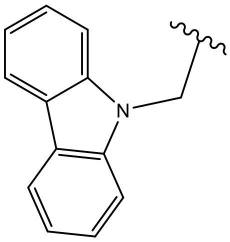	*-12.281*	*-62.41*	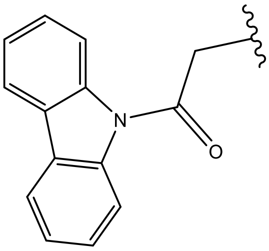	*-11.829*	*-66.46*
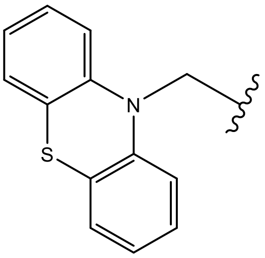	*-10.196*	*-49.63*	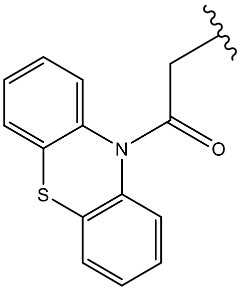	*-11.259*	*-77.52*
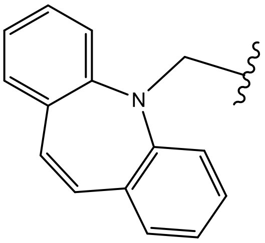	*-10.769*	*-79.97*	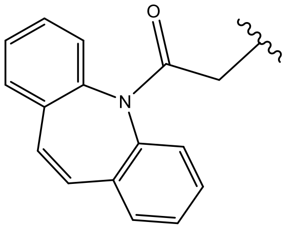	*-11.677*	*-75.63*
			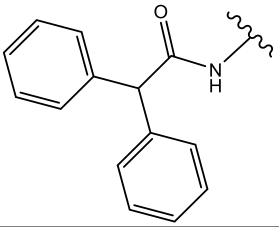	*-8.545**	*-78.64**

*Glide XP and MMGBSA dG Bind Scores of **Compound I** binding pose from 7AMZ complex.

The highest-scoring derivative (docking score -12.516 and MM-GBSA dG score -80.44), fluorene, was selected as a leading structure for further development (**Table S1**). The binding mode of compound **1** (**Figure S38**) revealed the fluorene system deeply entrenched in the hydrophobic pocket formed by Trp231, Phe329, and Phe398. Contrary to **compound I**, the benzyl fragment in compound **1** is oriented towards the interior of the binding site, forming aromatic interactions within the catalytic anionic site with Trp82. Docking simulations predominantly showed the benzylamine fragment forming a salt bridge with Asp70, further stabilized by a hydrogen bond with Tyr332 hydroxyl group, and positioning its aromatic ring inside the binding site. The results of a 100 nanosecond molecular dynamics experiment supported the stability of this binding conformation (**Figure S38B** and **Figure S39**) and the durability of important interactions (**Figure S40**).

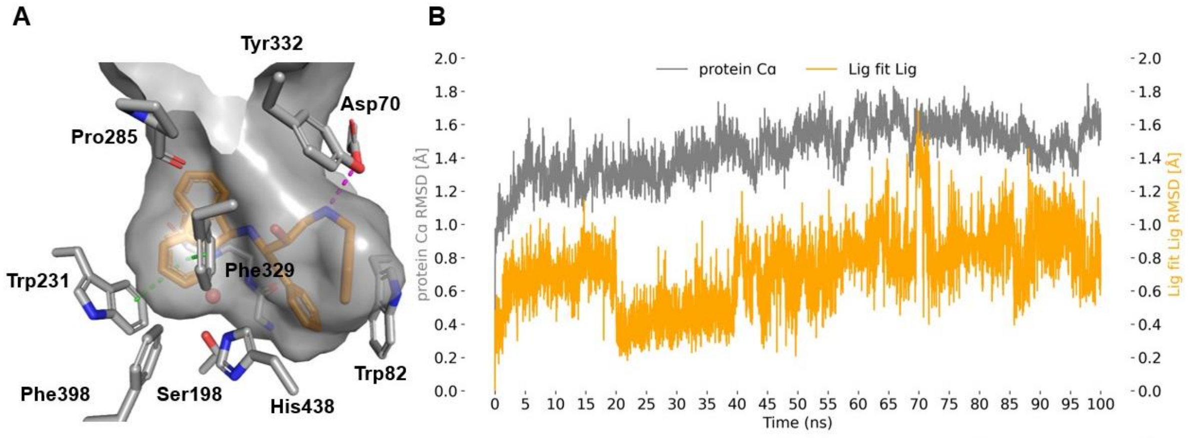

	Average RMSD [Å]	Maximum RMSD [Å]	Final RMSD [Å]
**Protein Cα**	1.445	1.846	1.568
**Lig fit Lig**	0.723	1.681	0.640

Figure S38A. A) Binding mode of compound **1** in the active site of BuChE obtained by molecular docking. Dashed lines indicate: Aromatic interaction (green), H-bonds (yellow), and salt bridges (magenta). B) RMSD evolution plot during a 100 ns MD simulation shows protein Cα (gray) and ligand (orange) aligned and measured on respective reference positions from the initial complex.

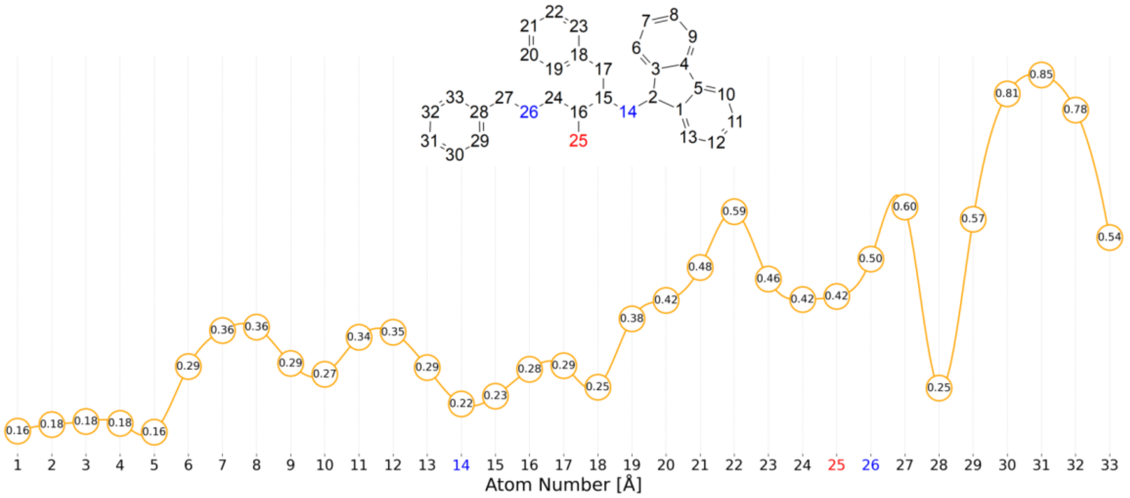

Figure S39. Root Mean Square Fluctuation (RMSF) values for each atom of molecule **1** after 100 ns of molecular dynamics simulation.

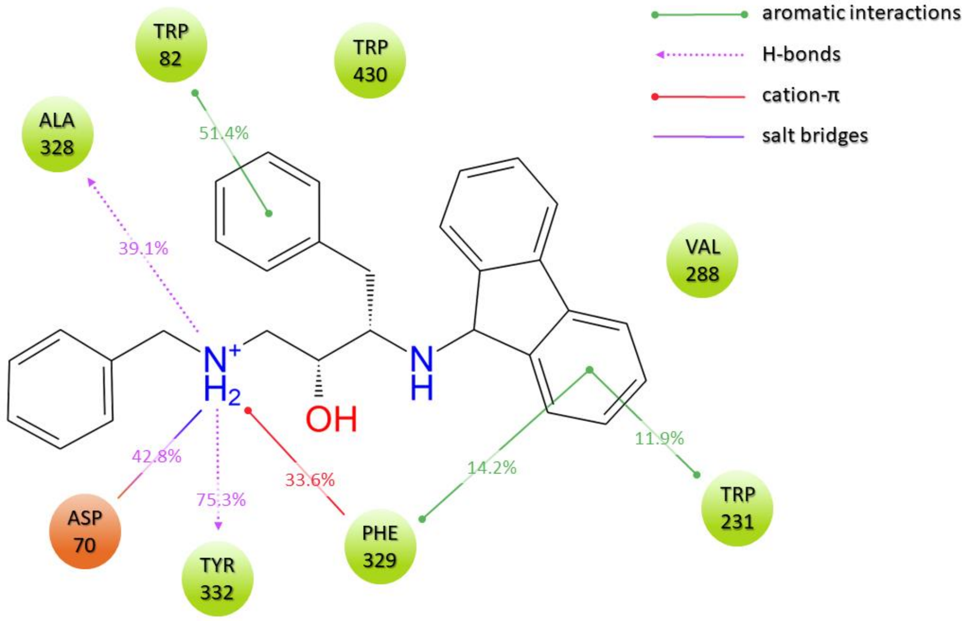

Figure S40. A schematic diagram of the interactions between compound **1** and the specific amino acids in the BuChE active site that are most crucial for ligand binding, including the respective percentage of occurrence observed during a 100 ns MD simulation.

A set of derivatives was synthesized to improve both the interaction with biological targets and the physicochemical properties, replacing the benzylamine group with aryl, alkyl, or cycloalkyl substituents. Thorough molecular modeling assessments of these compounds revealed a consistent binding mode and docking scores ranging from -11.503 to -13.938.

Further analysis using Prime MM-GBSA revealed that compound 3 exhibited a notably higher MM-GBSA dG Bind value of -66.02 compared to the rest (ranging from -77.35 to -81.98), which aligns with subsequent experimental findings. The molecular dynamics experiment for compound **6**, as shown in **Figure S41** and **Figure S42**, demonstrates a favorable outcome resulting from reducing the hydrophobic benzylamine group on cyclopropylmethylamine. This reduction leads to a stronger interaction between the benzyl group and Trp82 and an increased involvement of the hydroxyl group in polar interactions with Asp70 (**Figure S43**).

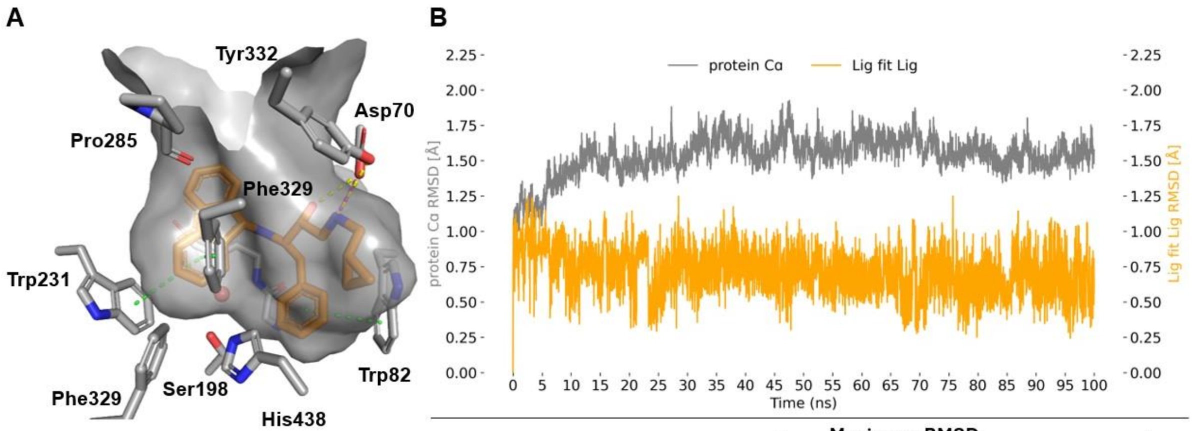

	Average RMSD [Å]	Maximum RMSD [Å]	Final RMSD [Å]
**Protein Cα**	1.537	1.926	1.574
**Lig fit Lig**	0.716	1.249	0.671

Figure S41. A) Binding mode of compound **6** in the active site of BuChE obtained by molecular docking. Dashed lines indicate: Aromatic interaction (green), H-bonds (yellow), and salt bridges (magenta). B) RMSD evolution plot during a 100 ns MD simulation shows protein Cα (gray) and ligand (orange) aligned and measured on respective reference positions from the initial complex.

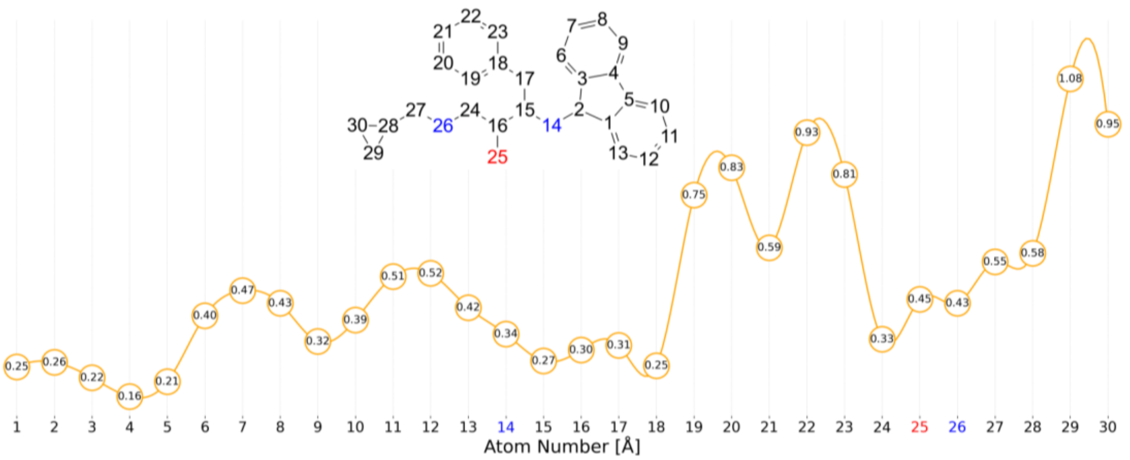

Figure S42. Root Mean Square Fluctuation (RMSF) values for each atom of molecule **6** after 100 ns of molecular dynamics simulation.

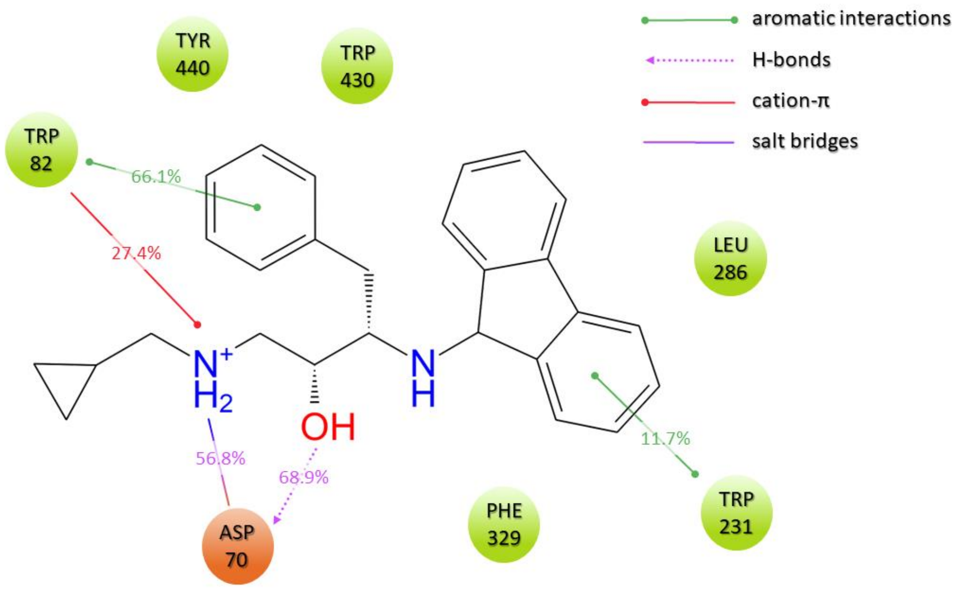

Figure S43. A schematic diagram of the interactions between compound **6** and the specific amino acids in the BuChE active site that are most crucial for ligand binding, including the respective percentage of occurrence observed during a 100 ns MD simulation.

Crystallography study

Binding mode of 6 to *h*BuChE (extended description)

The structure of **6** bound to human BuChE was determined by X-ray crystallography after protein-ligand complex formation by crystal soaking (**Table S2**). Extra electron density in the active site gorge allowed the fluorene and the benzyl motifs to fit unambiguously. At the same time, the fragment bearing the cyclopropane was slightly looser, pointing towards the solvent at the top of the active site gorge. The proximal ring of the fluorene engages a dense π-π interaction network with Trp231, Phe329, and, to a lower extent, Phe398, characterized by respective ring-ring distances of 5.5, 5.4, and 6.5 Å. Part of the choline-binding pocket is occupied by the benzyl motif interacting with Trp82 through π-π interactions with a ring-ring distance to the pyrrole ring of 4.0 Å. The fragment bearing the cyclopropane ring orients towards the gorge exit, engaging specific interactions through H-bonds. The nitrogen amine close to the cyclopropane forms an H-bond with the oxygen atom of the carbonyl of residue Pro285, while the hydroxyl oxygen atom engages both Asp70 and Tyr332, forming the BuChE peripheral site, with respective distances of 4.0 and 4.4 Å. It is important to note that this fragment should present some flexibility, which translates by the modelization of a minor alternate position of the ligand that varies mainly in the orientation of the cyclopropane-bearing fragment, with the hydroxyl pointing towards Pro285 and the amine pointing towards the peripheral site (**Figure S44**). The binding of this alternate position is probably less efficient when compared to the length of the H-bonds. This structure depicts the binding mode of **6** into human BuChE, how the ligand restricts substrate access to the catalytic site, and gives potential clues for further ligand optimization.

Structural analysis of the hBChE-6 complex by X-ray crystallography

For this study, the recombinant human BuChE was produced in Chinese hamster ovary cells[47] and purified with BuChE-specific chromatography (Hupresin®) followed by size exclusion chromatography.[27] Hanging drops were set up to grow crystals by vapor diffusion at 293 K using 0.1 M MES pH 6.5, 2.15 M $(NH_4)_2SO_4$ as crystallization buffer. Protein-ligand complex was obtained by soaking the crystals in a solution crystallization buffer containing 2

mM **6** (initially solubilized at 0.1 M in 100% methanol). Crystals were cryo-protected in a solution of 0.1 M MES pH 6.5, 2.15 M $(NH_4)_2SO_4$, and 20% glycerol, before flash cooling into liquid nitrogen. X-ray diffraction data were collected at the Proxima-1 beamline of the SOLEIL Synchrotron (Saint-Aubin, France) at 100 K. Recorded images were processed with XDSME.[28] The initial model was determined by molecular replacement using the human BuChE X-ray structure (PDB entry 1P0I) and the Phaser program of the Phenix software suite.[29] Iterative cycles of building and refinements, using respectively *Coot*[30] and Phenix.refine, allowed to fit ligand **6** which geometry restraints were generated with Phenix eLBOW[31] using the semi-empirical quantum mechanical method (AM1). Coordinates and structure factors of human BuChE in complex with **6** are available in the Protein Data Bank under accession number 9EWU.

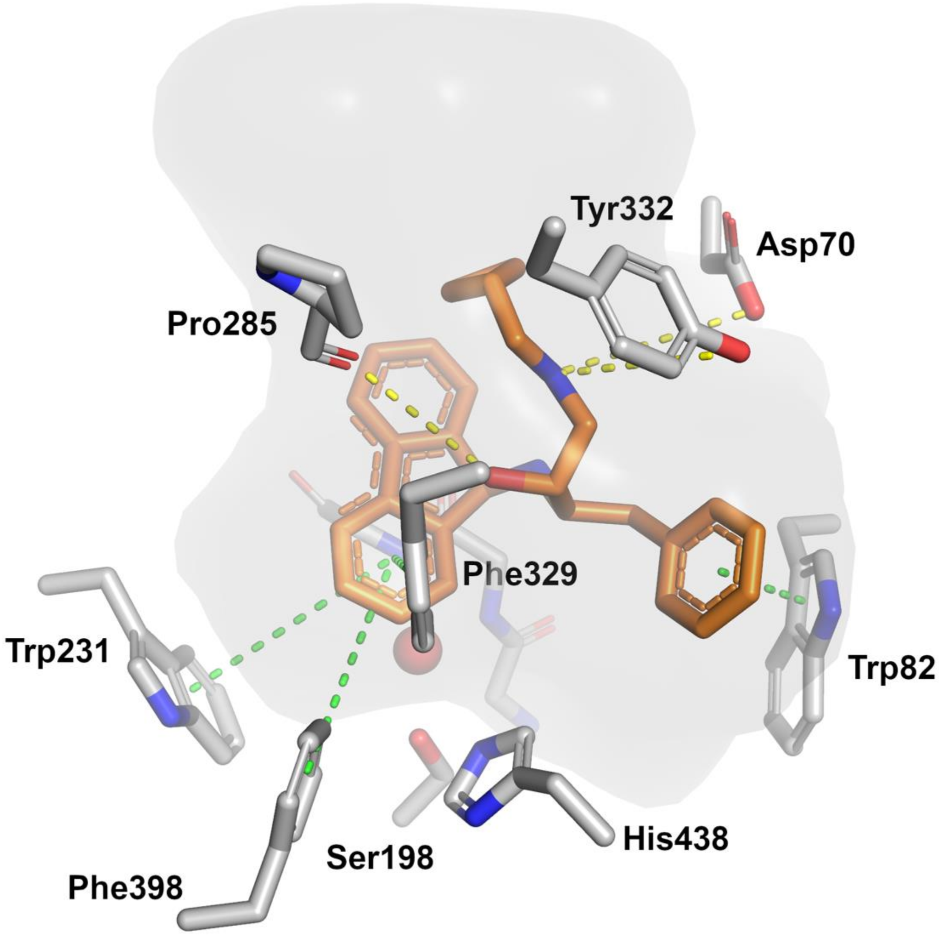

Figure S44. X-ray structure of the alternate position of **6** bound to human BuChE. A grey semi-transparent surface represents the enzyme active site gorge. Residues of human BuChE in interaction with **6** and the compound itself are represented as sticks with carbon atoms in orange or grey for **6** or protein residues, respectively. Nitrogen and oxygen atoms are represented in blue and red, respectively. A water molecule, depicted as a red sphere, is placed in the oxyanion hole. Specific interactions such as π-π and hydrogen bonds are represented as green and yellow dashed lines, respectively.

Table S2. Data collection and refinement statistics of human BuChE in complex with **6**, table calculated using Phenix. R-work=Σ |Fo - |Fc|| / Σ |Fo|, where Fo and Fc are observed and calculated structure factors, respectively. R-free set uses about 10% randomly chosen reflections. Statistics for the highest-resolution shell are shown in parentheses.

	***h*BuChE-6**
Data Collection	
**X-ray source**	SOLEIL Synchrotron Proxima-1
**Wavelength**	0.9786
**Resolution range**	38.91 - 2.45 (2.538 - 2.45)
**Space group**	I 4 2 2
**Unit cell**	154.6 154.6 128.52 90 90 90
**Total reflections**	770748 (79558)
**Unique reflections**	28852 (2854)
**Multiplicity**	26.7 (27.9)
**Completeness (%)**	99.91 (100.00)
**Mean I/sigma(I)**	14.88 (1.85)
**Wilson B-factor**	61.90
**R-merge**	0.1993 (2.596)
**R-meas**	0.2032 (2.643)
**R-pim**	0.03961 (0.4987)
**CC1/2**	0.998 (0.656)
**CC***	1 (0.89)
Refinement statistics	
**Reflections used in refinement**	28847 (2854)
**Reflections used for R-free**	1154 (115)
**R-work**	0.1888 (0.2922)
**R-free**	0.2443 (0.3392)
**CC(work)**	0.955 (0.795)
**CC(free)**	0.912 (0.656)
**Number of non-hydrogen atoms**	4587
**macromolecules**	4220
**ligands**	280
**solvent**	87
**Protein residues**	526
**RMS(bonds)**	0.009
**RMS(angles)**	0.99
**Ramachandran favored (%)**	94.27
**Ramachandran allowed (%)**	5.73
**Ramachandran outliers (%)**	0.00
**Rotamer outliers (%)**	1.54
**Clashscore**	5.44
**Average B-factor**	71.70
**macromolecules**	70.01
**ligands**	99.20
**solvent**	65.19
**Number of TLS groups**	1

In vitro pharmacological assays.

The *in vitro* inhibitory activity toward cholinesterases (*ee*AChE, *eq*BuChE, *h*BuChE)

The target compounds were tested for their inhibitory potency against cholinesterases using Ellman's protocol[32] modified for 96-well microplates. All the reagents were purchased from Sigma–Aldrich (Steinheim, Germany); only human butyrylcholinesterase (*h*BuChE) isolated from human plasma was from Vivonics (Bedford, MA, USA). The stock solutions of the target compounds were prepared in DMSO and diluted with water, giving the desired final concentrations. The enzymes were prepared as 5 U/mL aqueous stock solutions and diluted before use to a final concentration of 0.384 U/mL. Then 20 μL of prepared enzyme solutions (AChE or BuChE) were added to the reaction mixture in the wells, containing 25 μL of the target compound (in case of blank samples – water or water/DMSO mixture), 200 μL of 0.1 M phosphate buffer (pH = 8.0) and 20 μL of 5,5'-dithiobis-(2-nitrobenzoic acid) DTNB (0.0025 M). All those reagents were preincubated for 5 min at 25 °C for the reactions with the animal enzymes (*ee*AChE or *eq*BuChe) and 36 °C for the human enzyme (*h*BuChE) tests. The enzymatic reaction was initiated by the addition of 20 μL of substrate acetylthiocholine iodide ATC (0.00375 M) or butyrylthiocholine iodide BTC (0.00375 M) solutions (depending on the enzyme used). After 5 min of incubation, changes in absorbance were measured at 412 nm using the EnSpire multimode microplate reader (PerkinElmer, Waltham, MA, USA). Target compounds were tested at the screening concentrations of 10 μM or 1 μM for inhibitory potencies towards animal cholinesterases or human enzyme, respectively. Percent of enzyme inhibition was calculated based on the formula 100-(S/B)x100, where S and B were the respective enzyme activities with and without the test compound, respectively. Compounds with the enzyme inhibitory activities at 10 μM better than 50% (*ee*AChE or *eq*BuChe) and at 1 μM better than 75% (*h*BuChE) were further evaluated to obtain IC_{50} values. Calculations were based on the absorbance measured at seven different inhibitor concentrations, then converted to the % of enzyme inhibition using the above-presented formula. The obtained percentages of enzymes' inhibition were plotted against the applied inhibitor concentrations using nonlinear regression (GraphPad Prism 9; GraphPad Software, San Diego, CA, USA). Tacrine and donepezil were tested as the references. All the experiments were performed in triplicate.

The *in vitro* inhibitory activity toward AChE – results.

Table S3. Inhibition of AChE for compounds **1-6**.

Cmp.	***ee*AChE**
	% inh.[a]
**1**	<10%
**2**	<10%
**3**	<10%
**4**	<10%
**5**	19.9% ± 3.6
**6**	<10%
tacrine	0.023 ± 0.0004
donepezil	0.011 ± 0.0002

[a] %inh. at an inhibitor concentration of 10 µM

BuChE binding affinity

Differential scanning fluorimetry (DSF) – methodology

Butyrylcholinesterase was mixed with ProteoOrange (Lumiprobe) diluted in phosphate buffer pH 7,5 (final concentration 10x) in total volume 40 µL in the absence or presence of 1 µM an 5 µM 6 and tacrine diluted in DMSO. The DMSO concentration was 1% in the final mixture. Non-enzyme controls were included. DSF assay were performed in white strips using a qPCR cycler qTOWER^3/G (Analytik Jena). The melting curves were monitored in temperature range 25–99 °C with an increment of 1 °C/min using fluorescence Ex/Em = 492/610 nm. Raw fluorescence data were processed by online tool DSFworld[33] to determine melting temperature (T_m) of proteins itself or in the presence of compounds. The raw data were analyzed in the range of temperature 35 - 75°C by sigmoid fitting (fit2) reflecting the shape of measured melting curves. Changes in T_m between enzyme itself and in the presence of **6** and tacrine were statistically evaluated (the student's unpaired t-test). The T_m values are given as mean ± SD from three technical replicates.

DSF results

The proof of interaction between BuChE and compound **6** was done by the DSF method that characterizes the stability of a protein by melting temperature T_m. The presence of bound ligand stabilizes a protein and increases T_m.[34] The presence of 1 or 5 µM of **6** significantly increased T_m of BuChE similarly as standard inhibitor of BuChE tacrine which clearly points to an interaction between **6** and BuChE (**Figure S45**). Using two different concentrations

shows concentration-dependent stabilization of BuChE by **6** but it was not possible to predict K_d due to solubility issues.

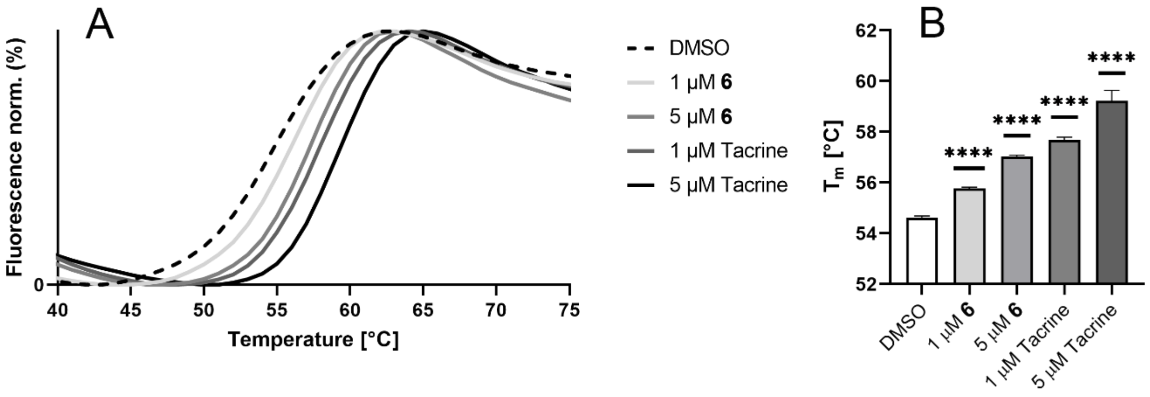

Figure S45. Temperature stability of BuChE in the presence or absence of **6** or tacrine measured by DSF method. Representative melting curves (A) as well as evaluated melting temperatures (T_m) (B). The T_m data represent mean ± SD (n=3). The differences between enzyme in the absence (DMSO) or presence of **6** or tacrine were determined by Student's unpaired t-test (*$p \leq 0.05$, **$p \leq 0.01$, ***$p \leq 0.001$, ****$p \leq 0.0001$).

The *in vitro* inhibitory activity toward *m*GATs (*m*GAT1 – *m*GAT4)

The inhibitory activities of obtained compounds toward four subtypes of *m*GAT were determined at a screening concentration of 100 µM and in the case of sufficient potency characterized in full scale [^{3}H] GABA uptake assays according to a procedure described before.[35] The inhibition of *h*GAT3 for the most potent compounds was studied in full-scale competition experiments as described.[36] MS binding assays for *m*GAT1 were performed as reported in Ref. [37] The screening concentration for tested compounds was 100 µM.

The *in vitro* affinity for mGAT1

Methodology

The affinity for mGAT1 was determined by MS Binding Assay with NO 711 as a non-labeled marker. Binding assays for mGAT1 based on NO 711 as native marker were performed as described earlier.[37] NO 711 was analyzed by LC–MS/MS using an API 3200 triple quadrupole mass spectrometer according to the method described previously. The compounds were considered active if NO 711 binding was reduced at least by 50 % at a concentration of

100 μM. For the active compounds, pK_i values were determined. All the samples were tested in triplicate in three independent experiments.

Results

Table S4. Binding affinities (pK_i) toward mGAT1 from MS Binding Assay of compounds **1-6**.

**Cmp.**	**pKi[a] ± SEM**
1	4.31
2	4.47
3	4.51
4	91%
5	72%
6	4.43
DDPM-1457	46.77

[a]pKi values from the [3H]GABA uptake or MS Binding Assays were determined in triplicate samples for competition and in three independent experiments only for compounds with pIC50 ≥ 5.00. The percent of remaining [3H]GABA uptake or NO711 binding is given in the presence of 100 μM inhibitor

FUNCTIONAL ACTIVITY

Functional assays for 5-HT1A receptor

Tested and reference compounds were dissolved in DMSO to the concentration of 10 mM. Dilutions were prepared in a 96-well microplate in assay buffers. For 5-HT1A receptors, adenylyl cyclase activity was determined using cryopreserved CHO-K1 cells expressing the human serotonin 5-HT1A receptor. The final concentration of DMSO in the test solutions was 0.1%.

The functional assay was performed with the CHO-K1 cells with expression of the 5-HT1A human serotonin receptor in which plasmid containing the coding sequence was transfected. The cells were cultured under selective conditions (400 μg/mL Geneticin G418) (PerkinElmer). Thawed cells were resuspended in stimulation buffer (HBSS, 5 mM HEPES, 0.5 IBMX, and 0.1% BSA at pH 7.4) at 2 × 105 cells/mL. The same volume (10 μL) of cell suspension was added to tested compounds with 10 μM forskolin. Samples were loaded onto a white opaque half-area 96-well microplate. Cell stimulation was performed for 40 min at room temperature. After incubation, cAMP measurements were performed with homogeneous time-resolved fluorescence resonance energy transfer (TR-FRET) immunoassay using the LANCE Ultra cAMP kit (PerkinElmer, USA). Ten microliters of EucAMP Tracer Working Solution and 10 μL of ULight-anti-cAMP Tracer Working Solution were added, mixed, and incubated for 1 h. The TR-FRET signal was read on an EnVision microplate reader (PerkinElmer, USA). E_{max} values were defined as the ligand response expressed as a percentage of the maximal response elicited by serotonin, determined by nonlinear regression using GraphPad Prism 9.0 software. pEC50 values correspond to the ligand concentration at which 50% of its maximal response was measured.

Table S5. Results of cAMP 5-HT1A functional assay study for compound **6**.

cAMP 5-HT1A functional assay												
Agonist mode*					Antagonist mode**							
	E max %	EC 50	pEC 50 ± SEM	R^2		E max %	IC 50	IC 50	pIC 50	Kb	pKb ± SEM	R2
	[10^{-5} M]	M					M	nM		M		Kb
SEROTONIN	100%	4.20E-09	8.38±0.98	0.964	NAN-190	100%	2.01E-09	2	8.70	4.63E-10	9.33±0.024	0.812
**6**	15%	n.c.	n.c.	n.c.	JT-3	16%	n.c.	n.c.	n.c.	n.c.	n.c.	n.c.

*Results were normalized as percentage of maximal agonist response (Serotonin 10^{-5} M)
**Results were normalized as percentage of maximal antagonist response (NAN-190 10^{-5}M)

E max is the maximum possible effect
n.c.- not calculable

Functional assays for 5-HT7 receptor

Test and reference compounds were dissolved in dimethyl sulfoxide (DMSO) at a concentration of 10 mM. Serial dilutions were prepared in a 96-well microplate in assay buffer, and 8 to 10 concentrations were tested. For the 5-HT7, adenylyl cyclase activity was monitored using cryopreserved CHO-K1 cells expressing the human serotonin 5-HT7 receptor. The functional assay was performed on cells expressing the human 5-hydroxytryptamine (serotonin) receptor 7. CHO-K1 cells were transfected with a beta-lactamase (bla) reporter gene under the control of the cyclic AMP response element (CRE) (Life Technologies). Thawed cells were resuspended in stimulation buffer (HBSS, 5 mM HEPES, 0.5 IBMX, and 0.1% BSA at pH 7.4) at $2x10^5$ cells/ml for 5-HT7 receptor. The same volume (10 µl) of cell suspension was added to tested compounds for the 5-HT7 receptor. Samples were loaded onto a white opaque half-area 96-well microplate. The antagonist response experiment was performed with 10 nM serotonin as the reference agonist for the 5-HT7 receptor. The agonist and antagonist were added simultaneously. Cell stimulation was performed for 1 h at room temperature. After incubation, cAMP measurements were performed with homogeneous TR-FRET immunoassay using the LANCE Ultra cAMP kit (PerkinElmer, USA). The volume of 10 µl of EucAMP Tracer Working Solution and 10 µl of ULight-anti-cAMP Tracer Working Solution were added, mixed, and incubated for 1 h. The TR-FRET signal was read on an EnVision microplate reader (PerkinElmer, USA). IC_{50} and EC_{50} were determined by nonlinear regression analysis using GraphPad Prism 9.0 software.

Table S6. Results of 5-HT7 receptor functional assay study for compound **6**.

**cAMP 5-HT7 functional assay**										
**Agonist mode***					**Antagonist mode****					
	**E max %**	**EC_{50}**	**pEC_{50}**	**R^2**		**E max %**	**IC_{50}**	**IC_{50}**	**pIC_{50}**	**R^2**
	**[10^{-5} M]**	**M**					**M**	**nM**		**IC_{50}**
SEROTONIN	100	2.92E-08	7.54	0.947	SB269970	100	1.50E-09	1.5	8.82	0.864
**6**	8	n.c.	n.c.	n.c.	JT-3	0	n.c.	n.c.	n.c.	n.c.

*Results were normalized as a percentage of maximal agonist response (Serotonin 10^{-5} M)

**Results were normalized as a percentage of maximal antagonist response (SB269970 10^{-5}M)

E max is the maximum possible effect

n.c.- not calculable

Functional assays for 5-HT6 receptor

Test compounds were dissolved in dimethyl sulfoxide (DMSO) at a concentration of 10 mM. Serial dilutions were prepared in a 96-well microplate in assay buffer, and 8 concentrations were tested. For the 5-HT6, adenylyl cyclase activity was monitored using cryopreserved 1321N1 cells expressing the human serotonin 5-HT6 receptor (Perkin Elmer, USA). Thawed cells were resuspended in stimulation buffer (HBSS, 5 mM HEPES, 0.5 IBMX, and 0.1% BSA at pH 7.4) at $2x10^5$ cells/ml. The same volume (10 µl) of cell suspension was added to the tested compounds. Samples were loaded onto a white opaque half-area 96-well microplate. The antagonist response experiment was performed with 22 nM serotonin as the reference agonist for the 5-HT6 receptor. The agonist and antagonist were added simultaneously. Cell stimulation was performed for 30 minutes at room temperature. After incubation, cAMP measurements were performed with homogeneous TR-FRET immunoassay using the LANCE Ultra cAMP kit (PerkinElmer, USA). The volume of 10 µl of EucAMP Tracer Working Solution and 10 µl of ULight-anti-cAMP Tracer Working Solution were added, mixed, and incubated for 1 h. The TR-FRET signal was read on an EnVision microplate reader (PerkinElmer, USA). IC 50 and EC 50 were determined by nonlinear regression analysis using GraphPad Prism 9.0 software.

Table S7. Results of 5-HT6 receptor functional assay study for compound **6**.

**cAMP 5-HT6 functional assay**												
**Agonist mode***					**Antagonist mode****							
	**E max %**	**EC 50**	**pEC 50 ± SEM**	**R2**		**E max %**	**IC 50**	**IC 50**	**pIC 50**	**Kb**	**pKb ± SEM**	**R2**
	**[10^{-5}M]**	**M**					**M**	**nM**		**M**		**Kb**
SEROTONIN	100	2.56E-09	8.29±0.12	0.947	SB258585	100	7.64E-09	7.64	8.12	2.04E-09	8.69±0.005	0.953
**6**	41	2.42E-06	5.62	0.978	JT-3	3	n.c.	n.c.	n.c.	n.c.	n.c.	n.c.

*Results were normalized as a percentage of maximal agonist response (Serotonin 10^{-5} M)
**Results were normalized as a percentage of maximal antagonist response (SB258585 10^{-5}M)
E max is the maximum possible effect
n.c.- not calculable

GABAA radioligand binding assay.

Method

Preparation of solutions of test and reference compounds

First, 10 mM stock solutions of tested compounds were prepared in DMSO. Serial dilutions of compounds were prepared in a 96-well microplate in assay buffers using the automated pipetting system epMotion 5070 (Eppendorf). Each compound was tested in a screening assay at final concentrations of 100, 10, and 1 μM. Results were expressed as percent inhibition of [^{3}H]-Flunitrazepam binding.

Radioligand binding assay for GABAA/BZP using [3H]-flunitrazepam

Radioligand binding assay was performed using tissue rat cortex. Rats' brains were homogenized in 20 volumes of ice-cold 50 mM Tris-HCl buffer (pH 7.4) using an ULTRA TURAX homogenizer. The homogenate is then centrifuged at 20.000 x g for 20 min (0-4°C). The resulting supernatant was discarded, and the pellet was rehomogenised in 20 volumes of ice-cold 50 mM Tris-HCl buffer (pH 7.4) and centrifuged as above. The pellet was resuspended and centrifuged further two times. The final pellet was stored at -80°C for at least 18 hours. On the day of the assay, the pellet was thawed at room temperature, resuspended in 20 volumes of ice-cold 50 mM Tris-HCl buffer (pH 7.4), and centrifuged at 20.000 x g for 25 min (0-4°C).

All assays were carried out in duplicates. 50 μl working solution of the tested compounds, 50 μl [^{3}H]-Flunitrazepam (spec. act. 80 Ci/mmol, final concentration 0.8 nM) and 150 μl tissue suspension prepared in assay buffer (50 mM Tris-HCl, pH 7.4) were transferred to polypropylene 96-well microplate using 96-wells pipetting station Rainin Liquidator (Mettler Toledo). Diazepam (10 μM) was used to define nonspecific binding. The microplate was covered with sealing tape, mixed, and incubated for 20 minutes at 4 °C. The reaction was terminated by rapid filtration through GF/B filter mate presoaked with 0.5% polyethyleneimine for 30 minutes. Five rapid washes with 300 μl 50 mM Tris buffer (4 °C, pH 7.4) were performed using a 96-well FilterMate harvester (PerkinElmer, USA). The filter mates were dried at 37 °C in a forced air fan incubator, and then, solid scintillator MeltiLex

was melted on filter mates at 90 °C for 4 minutes. Radioactivity was counted in the MicroBeta2 scintillation counter (PerkinElmer).

Results

Table S8. Radioligand binding assay for BDZ site using [^{3}H]-Flunitrazepam. Results are presented as a percentage of control-specific binding at 100, 10, and 1 μM concentrations.

No	COMPOUND ID	LOG OF MOLAR CONC.	% OF CONTROL SPECIFIC BINDING	LOG OF MOLAR CONC.	% OF CONTROL SPECIFIC BINDING	LOG OF MOLAR CONC.	% OF CONTROL SPECIFIC BINDING
1	**6**	-4	**26%**	**-5**	**21%**	**-6**	**8%**
2	**DIAZEPAM**	-4	**100%**	**-5**	**100%**	**-6**	**97%**
3	**ZOLPIDEM**	-4	**100%**	**-5**	**97%**	**-6**	**84%**

Norepinephrine Transporter Competition Binding Assay

Method

Preparation of solutions of test and reference compounds

First, 10 mM stock solutions of tested compounds were prepared in DMSO. Serial dilutions of compounds were prepared in a 96-well microplate in an assay buffer using the automated pipetting system epMotion 5070. Each compound was tested in 8 concentrations ranging from 10^{-3} to 10^{-10} M (final concentration).

Norepinephrine Transporter Competition Binding Assay

Radioligand binding was performed using membranes from the MDCK cell line stably transfected with the human norepinephrine transporter. All assays were carried out in duplicates. Volumes of 50 µl working solution of the tested compounds, 50 µl [^{3}H]-Nisoxetine (spec. act. 80.2 Ci/mmol, final concentration 4 nM), and 150 µl diluted membranes (5 µg protein per well) prepared in assay buffer (50 mM Tris-HCl, pH 7.4; 120 mM NaCl; 5 mM KCl) were transferred to polypropylene 96-well microplate using 96-wells pipetting station Rainin Liquidator. Desipramine (10 µM) was used to define nonspecific binding. The microplate was covered with sealing tape, mixed, and incubated for 60 minutes at 4 °C. The reaction was terminated by rapid filtration through GF/B filter mate presoaked with 0.5% polyethyleneimine for 30 minutes. Five rapid washes with 300 µl 50 mM Tris buffer (4 °C, pH 7.4) were performed using a 96-well FilterMate harvester. The filter mates were dried at 37 °C in the forced air fan incubator, and then, solid scintillator MeltiLex B/HS was melted on filter mates at 90 °C for 4 minutes. Radioactivity was counted in the MicroBeta2 scintillation counter (PerkinElmer). Data were fitted to a one-site curve-fitting equation with Prism 8.0 (GraphPad Software), and Ki values were estimated from the Cheng−Prusoff equation.

Result of Norepinephrine Transporter Competition Binding Assay

Table S9. Results of binding to norepinephrine transporter (NET) of tested compounds

COMPOUND	Ki [nM]	pKi	Ki [nM]	pKi	Ki [nM]	pKi
**6**	714.0	6.146	837.0	6.077	774.0	6.111

**Desipramine**	3.3	8.486	3.0	8.529	4.2	8.377
**Clomipramine**	102.0	6.991	197.0	6.705	117.0	6.933

In vitro ADME-tox study

Metabolic Stability

The *in silico* prediction of the most probable sites of metabolism was done by MetaSite 6.0.1 software (Molecular Discovery Ltd, Hertfordshire, UK). The metabolic pathways determination *in vitro* was performed by 120 min compounds incubation with human liver microsomes (HLMs) purchased from Sigma-Aldrich, St. Louis, MO, USA. The reactions were conducted in 10 mM Tris–HCl buffer (pH 7.4) at 37 ºC in the presence of NADPH Regeneration System (Promega, Madison, WI, USA). UPLC/MS analyses were done using the Waters ACQUITY TQD system with the TQ Detector (Waters, Milford, USA).

UPLC chromatograms of the reaction mixtures and the proposed structures of metabolites.

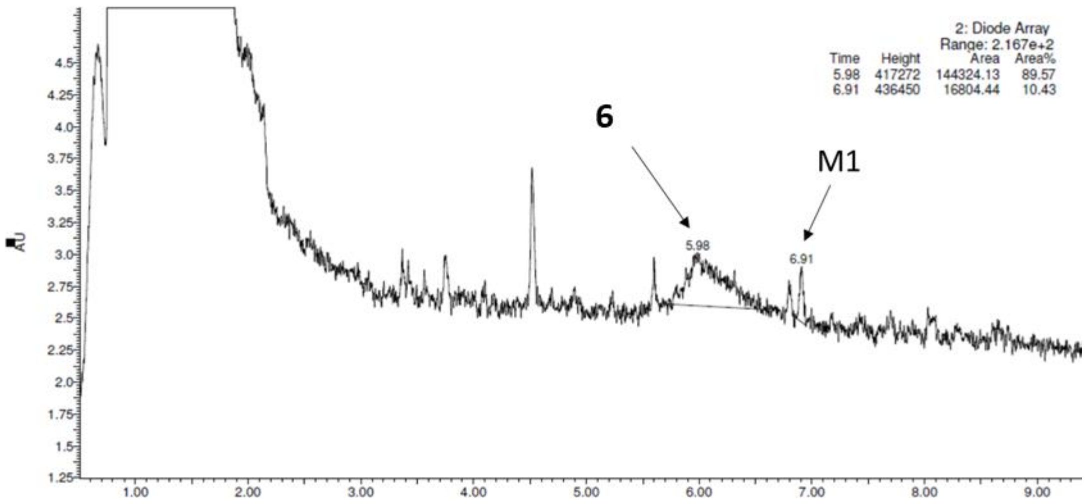

Figure S46. UPLC spectra after 120 min incubation of compound **6** with HLMs. 89.57% of the parent compound remained in the reaction mixture. One metabolite (M1, m/z = 460.30) was determined.

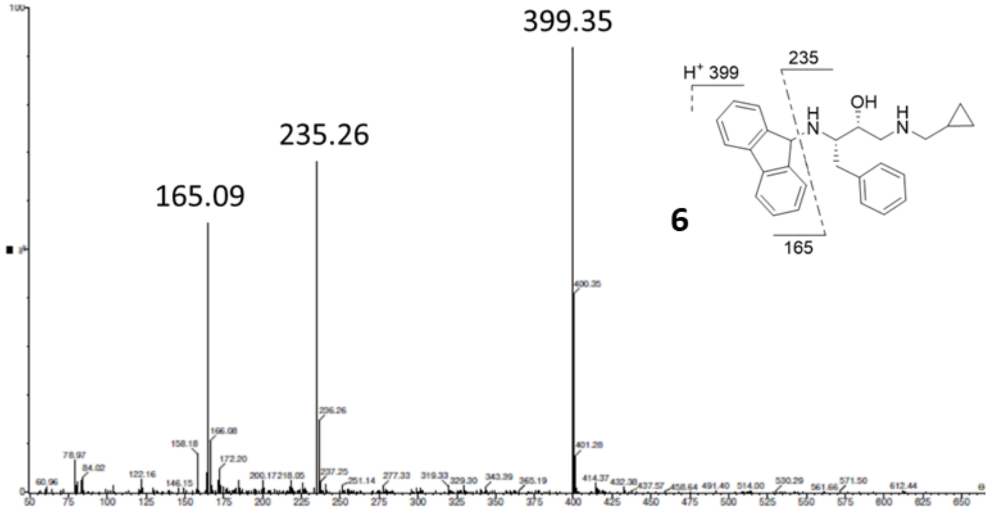

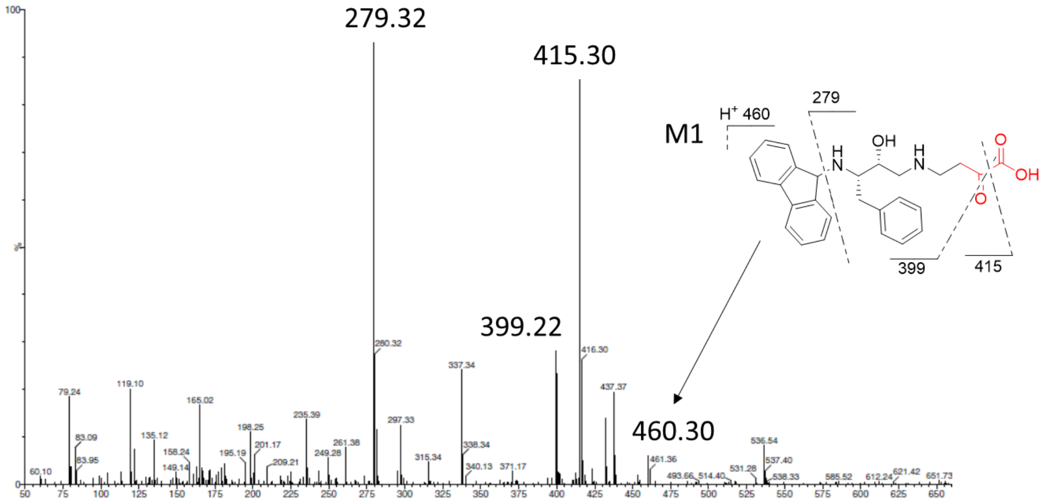

Figure S47. MS/MS ion fragment analyses and the most probable structures of **6** metabolite.

Cytotoxicity study

Cells preparation

The mouse microglial cells (BV-2) were cultured using standard procedures. The cells were a generous gift from professor Bozena Kaminska-Kaczmarek from Laboratory of Molecular Neurobiology, Neurobiology Center, Nencki Institute of Experimental Biology, Polish Academy of Sciences, Warsaw, Poland. The cells were cultured in Dulbecco's Modified Eagle's Medium - high glucose, (DMEM, Glutamx ThermoFisher) supplemented with 10% fetal bovine serum heat inactivated (ThermoFisher), with added 100 IU/ml penicillin (Sigma Aldrich) and 100 μg/ml streptomycin (Sigma Aldrich). The cells were cultured in flasks with

an area of 175 cm^2 (Nunc), and incubated at 37°C, 5% CO_2. For the test of compounds with the BV-2 cells line, microglia cells were seeded on 96-well culture plate (Falcon) at a density of $2x10^4$ cells per well in fresh medium. The cells were grown for 24 hours in the incubator (37°C, 5% CO_2) before performing experiments.

Mouse Hippocampal Neuronal (HT-22) were cultured in Dulbecco's modified Eagle's Medium - high glucose (DMEM, Glutamx Thermo Fisher) supplemented with 10% inactivated fetal bovine serum heat (Thermo Fisher), 100 IU/ml penicillin (Sigma Aldrich) and 100 µg/ml streptomycin (Merck). Cells were cultured in flasks (area 75 cm^2, Nunc), and incubated at 37°C, 5% CO_2. For the measurement of cell viability cells were placed in a 96-well culture plate ($2x10^4$ cells per well, Falcon). Before the tests, cells were grown for 24 hours in the incubator (37°C, 5% CO_2).

Preparation of stock solutions of tested compounds

Stock solutions were prepared in the concentration of 10 mM for test and reference compounds. Minimum 1 mg of each tested compound was weighed and dissolved in appropriate volume of dimethyl sulfoxide. Serial dilutions were prepared in DMSO and then the diluted compounds were transferred to PBS. Before assays eventual precipitation or opalescence was checked. All experiments were performed in duplicates, in three independent experiments. Cell viability was measured after 24 hours of incubation with tested cell line.

Cell viability assay

Cell viability was evaluated using the Presto Blue reagent (Thermo Fisher), according to the manufacturer procedures. Following 24 hours of incubation with the tested molecule, PrestoBlue reagent was added to a microplate well in an amount equal to one tenth of the remaining medium volume. The resulting mixture was incubated for 15 minutes at 37°C, and the fluorescence intensity (EX 530; EM 580 nm) was measured in the plate reader POLARstar Omega, (BMG Labtech). The results (viability values) are provided as a percentage of live cells with respect to DMSO (control sample).

Statistical analysis:

Viability values were calculated as a percentage of live cells with respect to the control sample (DMSO). The negative control was medium without cells. IC 50 was determined by

nonlinear regression analysis using GraphPad Prism 9.0 software. All values are expressed as mean with SD.

Results:

Table S10. The viability of cells.

	neurotoxicity		immuntoxicity	
μM	X [%]	SEM	X [%]	SEM
100 μM	1.7	0.2	1.3	0.2
50 μM	9.5	4.7	2.0	0.4
10 μM	99.0	3.0	94.8	0.7
1 μM	100.3	5.6	103.0	0.5
0.1 μM	99.0	1.5	102.5	1.0

The results (viability values) are provided as a percentage of live cells with respect to DMSO (control sample)

Table S11. The viability of cells.

	NEUROTOXICITY				
Cmp.	**IC 50**	**SEM**	**pIC**	**SEM**	**R2**
**6**	23. 1 μM	2.0 μM	4.64	0.04	0.818
	IMMUNOTOXICTY				
Cmp.	**IC 50**	**SEM**	**pIC**	**SEM**	**R2**
**6**	18.9 μM	0.1 μM	4.72	0.01	0.845

Inhibition of human CYP450 isoforms

Methodology

The inhibitory potential of compound **6** toward human CYP1A2, CYP2C9, CYP2D6, and CYP3A4 enzymes was assessed using Vivid® CYP450 Screening Kits (Thermo Fisher Scientific, Waltham, MA, USA). A six-point concentration gradient of compound **6** was prepared in reaction buffer and pipetted onto a black 96-well plate (Brand, Wertheim Germany) and then a mixture of the respective recombinant CYP isoenzyme and the NADPH regeneration system was added, following the manufacturer's instructions. The DMSO concentration was maintained at 0.5% in all reaction variants including controls. The plate was incubated at 37°C for 10 minutes, after which the enzymatic reaction was initiated by

adding NADP+ and the respective CYP isoenzyme substrate. After a further 10-minute incubation at 37°C, the formation of the reaction product was measured as fluorescence using a SPARK microplate reader (TECAN, Männedorf, Switzerland). The half-maximal inhibitory concentration (IC50) values were calculated by fitting the data to sigmoidal Hill curves using GraphPad Prism version 10.1.2. (GraphPad Software, La Jolla, CA, USA).

Results

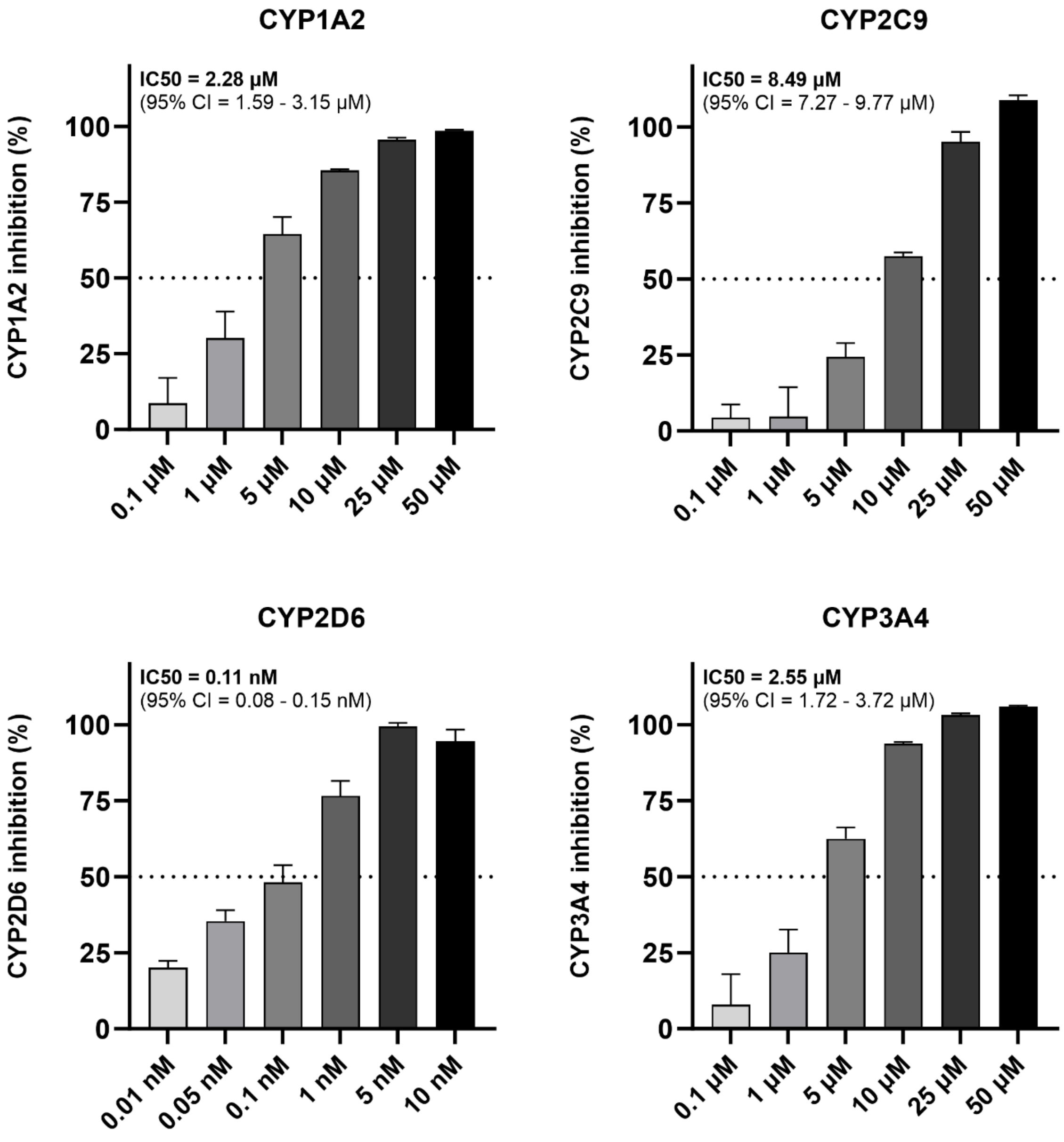

Figure S48. Effect of compound 6 on isoforms of cytochrome P450 activity (CYP1A2, CYP2C9, CYP2D6, CYP3A4) .

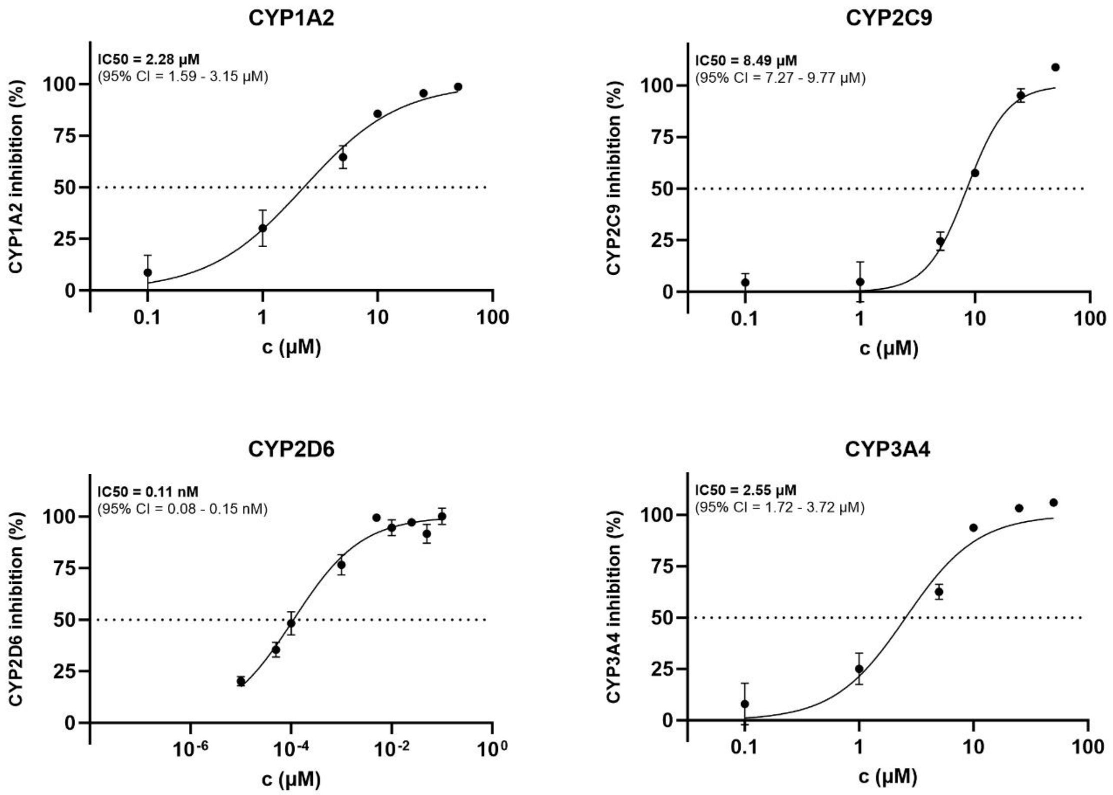

Figure S49. Effect of compound 6 on isoforms of cytochrome P450 activity (CYP1A2, CYP2C9, CYP2D6, CYP3A4) – curves.

Intracellular levels of RONS and MDA

Cell lines

Human neuroblastoma cells SH-SY5Y and mouse microglial cells SIM-A9 were used to evaluate cell viability, intracellular RONS, and malondialdehyde (MDA; CAS No. 542–78-9). SH-SY5y cells were cultivated in Dulbecco's modified Eagle's medium (DMEM; Biosera, Nuaille, France) supplemented with 10% fetal bovine serum (Biosera) and 1% penicillin-streptomycin antibiotic solution (Sigma-Aldrich, St. Louis, MO, USA). The culture medium for SIM-A9 cells was further supplemented with donor horse serum (Biosera). The cells were incubated at 37 °C in a CO_2 incubator (BINDER CO_2 incubator CB 160, Tuttlingen, Germany) and routinely passaged by trypsinization at 75–85% confluence.

Colorimetric Cell Viability Assay

The toxicological indices IC_{50} used in the present study were measured utilizing 3-(4,5-dimethylthiazol-2-yl)-2,5-diphenyl-tetrazolium bromide (MTT) reduction assay after

24 h incubation with tested compounds. For the assay, SH-SY5Y and SIM-A9 cells were seeded into 96-well clear plates (TPP, Techno Plastic Products AG, Trasadingen, Switzerland) in a 100 μL volume and density of 15 × 10^3 and 9 × 10^3 cells per well, respectively. Cells were allowed to attach overnight before the treatment. The stock solutions of tested compounds were prepared in dimethyl sulfoxide (DMSO, Sigma-Aldrich, St. Louis, MO) and next serially diluted in high glucose Dulbecco's Modified Eagle's medium (DMEM; 1-26F56-I, VWR Chemicals, Radnor, PA, USA). The concentration ranges of tested compounds were as follows: 3.125–400 μmol/L for *t*BHP and 0.78–100 μmol/L for compound **6**. The final concentration of DMSO did not exceed 1% (v/v). After 24 h incubation, the cultivation medium containing serially diluted substances was aspirated and replaced with a fresh medium containing MTT at a concentration of 0.5 mg/mL and subsequently incubated at 37 °C for 1 h. The medium with MTT was then aspirated, and formazan was dissolved in 100 μL of DMSO. Cell viability was assessed spectrophotometrically by the amount of formazan produced. Absorbance was measured at 570 nm with 650 nm reference wavelength on Spark (Tecan Group Ltd, Switzerland). The IC_{50} value was then calculated from the control-subtracted triplicates using non-linear regression (four parameters) of GraphPad Prism 9 software. Final IC_{50} and SEM values were obtained as a mean of at least three independent measurements. The concentrations of compound **6** and *t*BHP (positive control) corresponding to their IC_{50}, ½ IC_{50}, and ¼ IC_{50} values will be used to detect RONS by fluorescent probes and induce oxidative damage in both cell types.

	*t*BHP; IC_{50} (μmol/L) ± SEM	**6**; IC_{50} (μmol/L) ± SEM
SH-SY5Y	19.93 ± 1.35	3.38 ± 0.04
SIM-A9	60.37 ± 3.54	6.46 ± 0.09

Detection of Reactive Oxygen and Nitrogen Free Radicals

For the assay, the cells were seeded in a 100 μL volume and density of 15 × 10^3 (SH-SY5Y) and 9 × 10^3 (SIM-A9) cells per well into a black polystyrene microplate with clear bottom (BD Biosciences, Hampton, NH, USA). Two different fluorescent dyes, i.e., 2,7-dichlorodihydrofluorescein diacetate (DCFH-DA, Cayman Chemicals Company, Ann Arbor, MI) and dihydroethidium (DHE, Sigma-Aldrich, St. Louis, MO) were utilized for the determination of intracellular levels of RONS after 1, 4, and 24 h of incubation with tested

compound **6** or *t*BHP (positive control). DCFH-DA is a nonspecific fluorescent probe for the determination of several different RONS including hydrogen peroxide, hydroxyl radicals, or peroxynitrite, but excluding superoxide radical ($O_2^{\bullet -}$). DHE fluorescent dye was used for specific detection of intracellular $O_2^{\bullet -}$. Cells incubated with DMEM represented untreated control. After the incubation, the experimental medium was removed and replaced with a solution of DCFH-DA or DHE at concentrations of 20 and 5 μmol/L, respectively. The cells were subsequently incubated at 37 °C in a CO_2 incubator for 45 min. Afterward, the fluorescence intensity of each well was measured using a Spark multimode microplate reader at an excitation wavelength of 485 nm for DCFH-DA or 528 nm for the DHE probe and an emission wavelength of 535 or 590 nm, respectively. Each experiment was carried out in triplicate and repeated three independent times.[38]

LC-MS determination of total MDA

The total MDA content was determined using the procedure described by Váňová et al. (2023) as follows: The cell pellets were thawed at laboratory temperature, resuspended in 300 μL of ultrapure water (Millipore Purification System, Merck, Millipore, Darmstadt, Germany), and sonicated for 2 minutes using a Q500 homogenizer (QSONICA Sonicators, Newton) with a 2-second cycle at 20% amplitude. The 250 μL aliquot of resulting homogenate was spiked with 10 μL of 10 μmol/L d^2-MDA synthesized from 1,1,3,3-tetraethoxypropane-1,3-d2 (Cambridge Isotope Laboratories, Tewksbury). After the addition of 50 μL of 6 mol/L aqueous sodium hydroxide, the sample was placed in a heating block (DB-3 Sample Concentrator, TECHNE, Cole-Parmer, U.K.) at 60 °C for 30 minutes. The cooled sample was acidified with 150 μL of 35% trichloroacetic acid (v/v) and centrifuged at 3500g at 4 °C for 10 min (IEC CL31R Multispeed Centrifuge, Thermo Fisher Scientific, San Jose). Then, 400 μL of the supernatant was mixed with 25 mmol/L 2,4-dinitrophenylhydrazine solution. The sample was derivatized with continuous shaking at 300 RPM in a Thermomixer Comfort (Eppendorf, Hamburg, Germany) at 37 °C for 60 min and cleaned on Phenomenex STRATA C18-E 100 mg/1 mL (55 μm, 70 Å) SPE cartridges (Phenomenex, Torrance, USA). The eluent was evaporated to dryness under the stream of nitrogen in the sample concentrator and reconstituted in 100 μL of 70% MeOH (v/v). Using the same procedure, calibration standards were prepared by spiking the blank cell homogenate with MDA solution to achieve final concentrations ranging from 0.2 to 2.0 μmol/L, including the zero calibrator. The LC-MS/MS analysis was carried out on the Shimadzu Prominence LC-20 system (Shimadzu, Kyoto, Japan) coupled with a Thermo Finnigan LCQ Advantage

Max Ion Trap mass spectrometer equipped with the atmospheric pressure chemical ionization (APCI) probe (Thermo Fisher Scientific) operating in a positive ionization mode. A 20 μL of sample was injected onto a Phenomenex KINETEX C18(2) chromatographic column (150 mm × 3 mm, 2.6 μm, 100 Å) protected by a Security Cartridge (4 mm × 2 mm; Phenomenex, Torrance) at 40 °C, with a constant flow rate of 0.270 mL/min of the mobile phase consisting of 0.1% aqueous formic acid and MeOH (JT Baker, Avantor, Gliwice, Poland). Data were acquired in the selected reaction monitoring mode (SRM) with ion transitions *m/z* $[M + H]^+$ 235 → 159, 189 for MDA and *m/z* $[M + H]^+$ 237 → 161, 191 for d^2-MDA.

The determined concentrations of MDA were normalized to the intracellular protein levels measured by the Bradford protein assay. A 10 μL aliquot of cell homogenate was diluted fivefold with water, and 10 μL of the diluted samples was mixed with 200 μL of commercial Bradford reagent. The absorbance was measured at 595 nm, using Bovine Serum Albumin for quantification within the range of 0.1 to 1.0 mg/mL. If not specified differently, all chemicals and reagents were purchased from Sigma-Aldrich/Merck (Darmstadt, Germany).

Results

The intracellular level of RONS was increased in SH-SY5Y cells (**Figure S50A**) treated with *t*BHP in concentration corresponded to IC_{50} 1.25- and 1.11-fold compared to untreated control after 1 and 4 h incubation, respectively. After 24 h incubation with this concentration decrease of RONS was observed. After 1 h incubation with a concentration corresponding to ½ IC_{50} was determined 1.12-fold higher level of RONS in cells.

In SIM-A9 cells (**Figure S50B**) only in 1 h incubation with the IC_{50} concentration the RONS in cells were 1.21-times higher. On the contrary, in 24 h interval, the decreasing level of RONS was detected in cells treated with *t*BHP (1.30 times lower) and with compound **6** (1.63 times lower) both in the IC_{50} concentration. No significant increase in intracellular RONS was observed after treatment with compound **6** in both cell lines (**Figure S50A**, S**50B**).

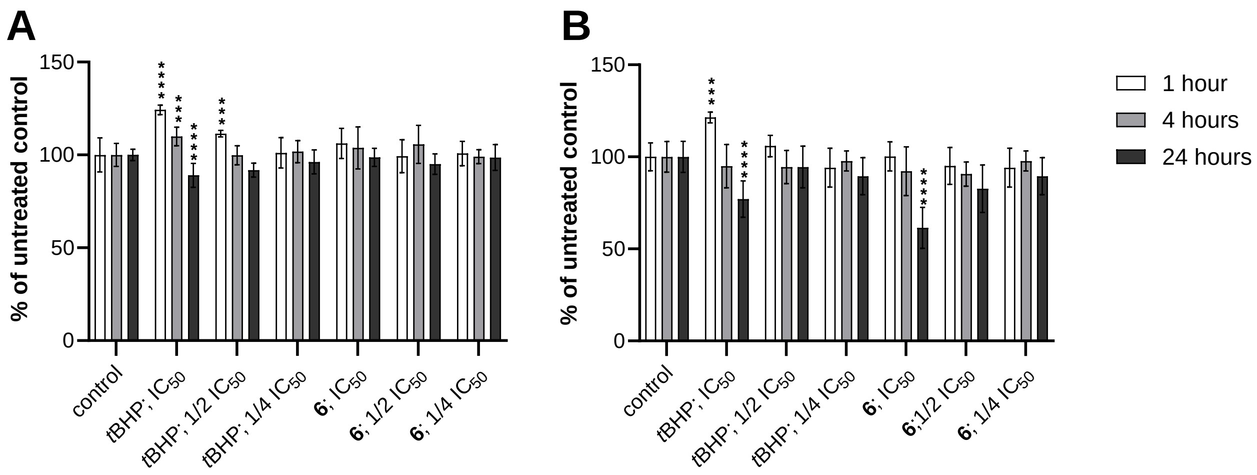

Figure S50. Changes in intracellular levels of RONS in SH-SY5Y (A) and SIM-A9 (B) cells determined using DCFH-DA fluorescent probe after 1 h (white column), 4 h (grey column), and 24 h (black column) treatment with *t*BHP and compound **6** ($n = 3$). Results are expressed in % of RONS of untreated controls (cells incubated only with the DMEM). One-way analysis of variance (ANOVA) followed by Dunnett's multiple comparison test was used for statistical analysis. Significant differences between treated cells and untreated control are indicated: * ($p \leq 0.05$), ** ($p \leq 0.01$), *** ($p \leq 0.001$), or **** ($p \leq 0.0001$).

The level of intracellular $O_2^{\bullet-}$ was detected with a DHE probe. In the 1 h interval, the intracellular $O_2^{\bullet-}$ in the SH-SY5Y cells (**Figure S51A**) was higher in all tested concentrations of *t*BHP (1.25-, 1.22-, and 1.10-times higher in concentration IC_{50} ½ IC_{50}, and ¼ IC_{50}, respectively). The same effect was observed also in 4 h intervals (1.23-, 1.16-, and 1.10-times higher in concentration IC_{50}, ½ IC_{50}, and ¼ IC_{50}, respectively). Finally, after 24 h incubation the level of $O_2^{\bullet-}$ was 1.11- and 1.10-fold higher in cells treated with IC_{50} and ½ IC_{50} concentration, respectively, compared to untreated cells. No significant increase in intracellular $O_2^{\bullet-}$ was observed after treatment with compound **6** in SH-SY5Y cells (**Figure S51A**). On the other hand, the levels of $O_2^{\bullet-}$ significantly decreased by 15% after 24 h incubation with the highest concentration of compound **6** corresponded to IC_{50} value.

In the SIM-A9 cells (**Figure S51B**), the intracellular $O_2^{\bullet-}$ was increased after treatment with IC_{50} concentration in all incubation intervals (1.11- for 1 h, 1.10- for 4 h, and 1.25 times higher for 24 h). Also, the ½ IC_{50} concentration caused the increase of $O_2^{\bullet-}$ in the cells in all time intervals (1.10- for 1 h, 1.10- for 4 h, and 1.32 times higher for 24 h). In the cells incubated with a concentration corresponding to ¼ IC_{50}, the level of $O_2^{\bullet-}$ increased only in 24 h intervals (1.27-fold higher).

Also, in the SIM-A9 cells (**Figure S51B**), no significant increase in intracellular $O_2^{\bullet-}$ was observed after treatment with compound **6**. Contrary, the levels of $O_2^{\bullet-}$ significantly decreased by 26% after 24 h incubation with the highest concentration of compound **6** corresponding to IC_{50} value.

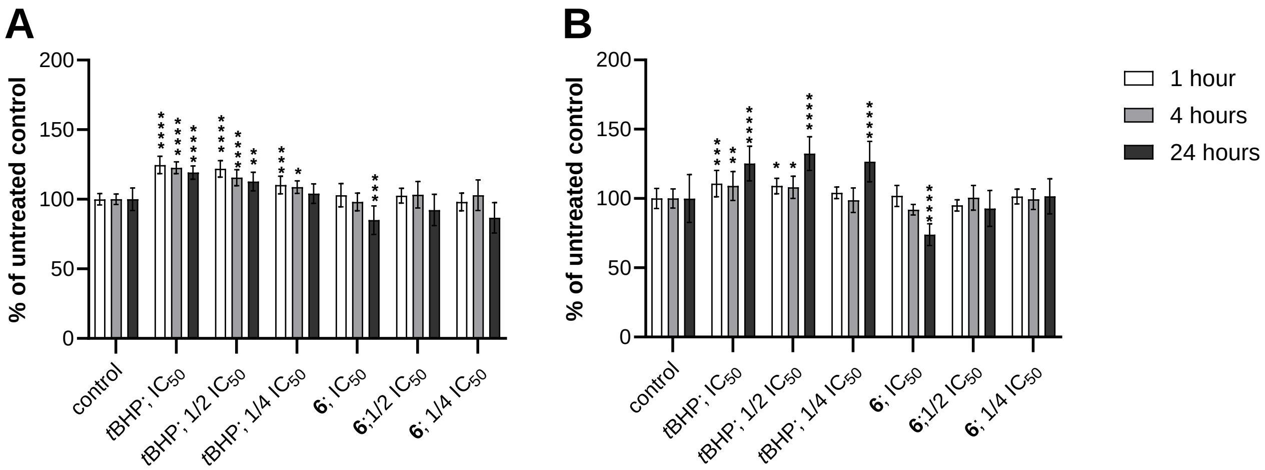

Figure S51. Changes in intracellular levels of $O_2^{\bullet-}$ in SH-SY5Y (A) and SIM-A9 (B) cells determined using a DHE fluorescent probe after 1 h (white column), 4 h (grey column), and 24 h (black column) treatment with *t*BHP and compound **6** (n = 3). Results are expressed in % of RONS of untreated controls (cells incubated only with the DMEM). One-way analysis of variance (ANOVA) followed by Dunnett's multiple comparison test was used for statistical analysis. Significant differences between treated cells and untreated control are indicated: * ($p \leq 0.05$), ** ($p \leq 0.01$), *** ($p \leq 0.001$), or **** ($p \leq 0.0001$).

After 1 h incubation, MDA levels in SH-SY5Y cells (**Figure S52A**) were 1.45-, 1.18-, and 1.23-times higher in the concentration IC_{50}, ½ IC_{50}, and ¼ IC_{50}, respectively. At 4h, MDA significantly increased in IC_{50} and ½ IC_{50} treated cells, being 1.45- and 1.18-fold higher compared to untreated cells, respectively. The level of MDA was 1.61-, 1.20-, and 1.11 times higher after 24 h incubation in a concentration corresponding to IC_{50}, ½ IC_{50}, and ¼ IC_{50}, respectively.

In SIM-A9 cells (**Figure S52B**), the MDA significantly increased in 1 h intervals only in cells treated with the highest tested concentration (1.18 times). At 4h, MDA increased in all tested concentrations (1.27- for IC_{50}, 1.18- for ½ IC_{50}, and 1.16 times higher for ¼ IC_{50}). Significantly higher levels of MDA were observed after 24 h treatment with the concentrations corresponding to IC_{50} (1.45-fold higher) and ½ IC_{50} (1.15-fold higher).

No significant increase in intracellular level of MDA was observed after treatment with compound **6** in both cell lines in all time intervals (**Figure S52A** and **Figure S52B**).

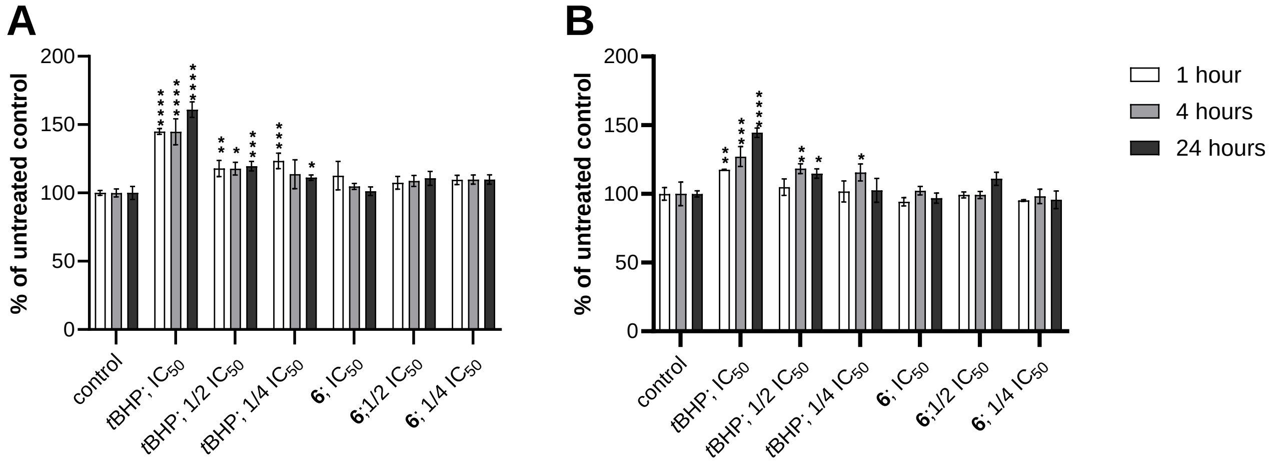

Figure S52. Changes in intracellular levels of MDA in SH-SY5Y (A) and SIM-A9 (B) cells determined by LC-MS/MS after 1 h (white column), 4 h (light grey column), and 24 h (dark grey column) of treatment with oxime AChE reactivators (*n* =). Results are expressed in % of RONS of untreated controls (cells incubated with the oxime-free DMEM). One-way analysis of variance (ANOVA) followed by Dunnet's multiple comparison test was used for statistical analysis. Significant differences between oxime-treated and untreated control groups are indicated: * ($p \leq 0.05$), ** ($p \leq 0.01$), *** ($p \leq 0.001$), or **** ($p \leq 0.0001$).

Toxicology and pharmacokinetic *in vivo* study

Animals

ICR-CD1 mice weighing 20–25 g were purchased from Velaz (Prague, Czech Republic). They were kept in an air-conditioned room with the light from 07:00 to 19:00 hours and were allowed access to standard food and tap water *ad libitum*. The mice were divided into groups of 8 animals.

Ethical approval statement

The experimental animals were handled under the supervision of the Ethics Committee of the Faculty of Military Health Sciences, Czech Republic (No. 20/19; MO 215611/2019-684800; 706288/2023-1457) and the treatment of animals was in full accordance with ethical standards laid down in respective Czech and EU regulations.

Design of *in vivo* experiments

Acute toxicity study – Maximal tolerated dose, biochemical analysis, and histopathologic examination in mice

Mice (ICR-CD1, Velaz, Prague, Czech Republic) were randomly assigned to the experimental groups of two males and two females per one applied dose of **6**. **6** was administered intraperitoneally (i.p., 0.1 mL/10 g of animal weight; 1% Tween 80). Several doses were administered to identify the toxic profile of tested compounds and establish the Maximal Tolerated Dose (MTD, i.p.). Treated animals were intensively observed for signs of toxicity, such as respiratory, cardiovascular, and nervous system disability. Weight loss or reduced food consumption was assessed according to the FELASA classification[39] in the first two hours and then periodically for the subsequent 46 hours. The toxicity signs were classified according to a previously published study.[40]

All animals surviving the 48 hours were deeply euthanized by CO_2 and subjected to basic macroscopic necropsy. The blood was collected for biochemical analysis, and selected organs (kidney and liver) were collected for histopathologic examination.

Venous blood was collected into heparinized tubes (Scanlab Systems, Prague, Czech Republic). Plasma was subsequently separated (3,000 × g for 10 min, 4 °C) using centrifuge U-320R (Boeco, Hamburg, Germany). The concentration of urea and creatinine and the activity of alanine aminotransferase (ALT), aspartate aminotransferase (AST), alkaline

phosphatase (ALP), amylase, and lactate dehydrogenase (LDH) were measured in the accredited biochemical laboratory of the Department of Clinical Biochemistry and Diagnostics, University Hospital, Hradec Kralove, Czech Republic. Statistical data analysis was performed through IBM SPSS Statistics version 26 (IBM, Armonk, NY, USA) using the Kolmogorov-Smirnov and Shapiro-Wilk tests to test data normality. Due to normally distributed data, the Student´s t-test was used to assess the differences compared with the control group ($p \leq 0.05$).

Samples of the liver and kidney were used for standard histopathological examination. Organs were fixed with 10% neutral buffered formalin (Bamed s.r.o., Ceske Budejovice, Czech Republic), histologically processed, and stained with hematoxylin and eosin (both Merck) according to Pejchal et al.[41] The histopathological changes were evaluated using a BX-51 microscope (Olympus, Tokyo, Japan).

Dose safety verification after oral administration

Mice were randomly assigned to the experimental groups of two males and two females per one selected dose (10 mg/kg) of **6** to verify its safety. **6** was administered by a probe (i.g., 0.1 mL/10 g of animal weight; 1% Tween 80). Treated animals were intensively observed for signs of toxicity; then, the animals were subjected to macroscopic examination. The blood was collected for biochemical analysis of glucose, urea, and creatinine concentration and the activity of alanine transaminase (ALT), aspartate transaminase (AST), gamma-glutamyl transferase (GGT), alkaline phosphatase (ALP), amylase, and creatine kinase (CK). Selected organs (terminal esophagus, stomach, duodenum, pancreas, kidney, and liver) were collected for histopathologic examination.

Pharmacokinetic study after i.p. and i.g administration

After i.p. and i.g. administration (0.1 mL/10 g of animal weight, 1% Tween 80) of **6** (10 mg/kg), blood samples were collected from mice under deep terminal anesthesia directly by cardiac puncture into heparinized 1.5 mL tubes at selected time intervals. The time intervals for i.p. were 0, 3, 5, 10, 20, 30, 45, 60, 90, 120, 240, and 360 minutes, and for i.g. 0, 30, 60, 90, 120, 180, 240, 480, 640, and 1440 minutes (six animals [n = 6] per time interval in both PK studies). The samples were centrifuged at 3,000 × g for 10 min (10 °C), and the plasma samples were stored at -80 °C until the HPLC analysis.

The blood in brain vessels would interfere with the assay of actual brain tissue concentrations. Therefore, the animals were perfused transcardially with saline solution (0.9% NaCl) for 5 min (10 mL/min). After perfusion, the skull was opened, and the brain was carefully removed and immediately stored at -80 °C until the HPLC analysis.

UHPLC-MS method for pharmacokinetics study

Ultra high-performance liquid chromatography system consisting of Ultimate 3000 RS pump Dionex (Thermo Scientific, San Jose, CA, USA) coupled with high-resolution mass spectrometer with orbitrap analyzer (Q Exactive Focus, Thermo Scientific, San Jose, CA, USA) was used in quantitative analyses of **6** in plasma and brain homogenate. According to the EMA[43] and FDA[42] guidelines on Bioanalytical method validation, the method was fully validated for plasma and partially for brain homogenate. All the evaluated parameters met the required criteria. Detailed validation data are given in **Table S12.**

Table S12. Summary of validation results (precision and accuracy) in plasma for five different concentrations - limit of quantitation(LLOQ), low (QC A), mid (QC B), high (QC C) a dilution (QC D) level.

		Compound 6				
		LLOQ	QC A	QC B	QC C	QC D
	ng/mL	0,50	1,25	33,50	452,73	45,27
PLASMA	**Within-batch 1**					
	Mean (ng/mL)	0,46	1,14	33,63	475,77	42,59
	Precision (%CV)	3,3%	4,1%	1,4%	6,5%	3,5%
	Accuracy (%)	-7,1%	-8,4%	0,4%	5,1%	-5,9%
	**Within-batch 2**					
	Mean (ng/mL)	0,44	1,16	30,70	477,27	40,41
	Precision (%CV)	9,5%	4,9%	3,5%	3,2%	2,9%
	Accuracy (%)	-11,9%	-6,9%	-8,4%	5,4%	-10,7%
	**Within-batch 3**					
	Mean (ng/mL)	0,49	1,12	33,25	452,50	44,01
	Precision (%CV)	7,8%	2,7%	2,8%	1,9%	3,9%
	Accuracy (%)	-2,8%	-10,2%	-0,7%	-0,1%	-2,8%
	**Batch-to-batch**					
	Mean (ng/mL)	0,46	1,14	32,53	468,51	42,33
	Precision (%CV)	7,9%	4,0%	4,8%	4,8%	4,8%
	Accuracy (%)	-7,3%	-8,5%	-2,9%	3,5%	-6,5%
PLASMA	**Matrix effect %**		105,1 ± 6,4%	104,9 ± 5,6%	110,1 ± 8,8%	
	**Recovery %**		80,1 ± 5,4%	72,6 ± 9,9%	76,7 ± 7,8%	
	**Autosampler stability % (24h)**			97,6 ± 1,5%	94,6 ± 2,5%	
	**Freeze-thaw stability % (3 cycles)**			95,8% ± 4,0	102,0 ± 4,7%	
	**Long-term stability % (3 month)**			109,9 ± 0,5%	105,7 ± 2,2%	
BRAIN	**Matrix effect %**		106,3 ± 3,4%	104,0 ± 3,8%	107,8 ± 3,0%	
	**Recovery %**		85,8 ± 8,1%	84,9 ± 5,8%	88,1 ± 8,1%	

Matrix effect normalized with IS and recovery for plasma and brain homogenate

Stability evaluation in rat plasma as deviation from the reference value (100%)

Table S13. Reproducibility of calibration curves of Compound 6 for plasma (n=6) and brain homogenate (n=4)

	PLASMA	BRAIN
Calibration model	Linear fit	Linear fit
Weighting	1/x^2	1/x^2
Calibration range (ng/mL)	0.5-603.6	0.5-603.6
Coefficient of determination R^2	>0.996	>0.995
Regression coefficient *a*	0.0291	0.0310
Standard deviation SD(*a*)	0.0017	0.0021
Relative standard deviation RSD(*a*)	5.8 %	6.7 %

Before analysis, 50 µL of plasma or brain homogenate was precipitated with an internal standard of 200 µL acetonitrile (ACN). The mixture was vortexed for 1 min at 1,500 rpm and centrifuged at 12,074 g for 5 min at 20 °C. The supernatant was then transferred to an injection vial and analyzed.

Chromatographic separation was carried out on a Luna Omega 1.6 µm Polar C18, 50 × 2.1 mm ID (Phenomenex, Torrance, CA, USA), protected with a guard column SecurityGuard Ultra C18 (Phenomenex, Torrance, CA, USA). The separation with a total run time of 4.5 min was performed in gradient elution mode with a flow rate of 0.6 mL/min and the following composition of mobile phase: A) 0.1% FA in water (v/v) and B) 0.1% FA in ACN/MeOH in ratio 50/50 (v/v). The gradient program was as follows: 0% of solvent B in 0–0.33 min, 0–100% of solvent B in 0.33–2.33 min, 100% of solvent B in 2.33–3.42 min, and column equilibration with 0% of solvent B in 3.42–4.5 min. The column was kept at 45 °C during analysis. Samples were held at 10 °C in a light-tight autosampler unit.

Heated-Electrospray Ionization II interface (HESI-II) in positive ion mode was used with the following settings: spray voltage; +3.5 kV, S-lens RF level; +70 V, capillary temperature 350 °C, auxiliary gas heater temperature; 350 °C, sheet, and auxiliary gas flow; 45 and 15 arbitrary units, respectively. Full MS mode was used at resolution (m/Δm) ~70,000 for analysis of **6** ($[M+H]^+$ = 399.24309) and internal standard Sertralin-d3 ($[M+H]^+$ = 309.09991). Xcalibur software (v 4.0) was used for data evaluation.

Pharmacokinetics study data evaluation

The pharmacokinetic profile is calculated as mean ± SEM (n = 6) using GraphPad Prism version 6.05 (GraphPad Software, Inc., San Diego, CA, USA).

Standard noncompartmental analysis was performed using the Kinetica software, version 4.0 (InnaPhase Corporation, Thermo Fisher Scientific Inc., Waltham, MA, USA). The observed

data determined the maximum concentration (C_{max}) and the time to the maximum concentration (Tmax). The area under the mean plasma concentration-time curve from zero to infinity (AUC_{total}) was determined as the sum of the $AUC_{0–240\ min}$ and of the extrapolated part, i.e., the ratio of the concentration predicted at the time interval of 24 hours and the terminal rate constant λz. The λz was estimated using linear regression of the log-transformed concentrations from 10 minutes to 4 hours plotted against time. The half-life was calculated as $t_{1/2} = \ln(2)/\lambda z$.

Results of toxicological and pharmacokinetic *in vivo* study

Acute toxicity study

According to clinical observations, MTD of i.p. administered **6** was estimated at 10 mg/kg in both male and female mice. At this dose, mild to moderate signs of poisoning may occur. The observed symptoms are summarized in **Table S14**. Signs and symptoms of toxicity of animals i.p. administered with **6** at doses of 20, 40, and 60 mg/kg are viewed in **Tables S19 and S20**.

Table S14. Symptoms observed in male and female ICR-CD1 mice when administered i.p. with **6** at 10 mg/kg (MTD).

**Symptoms of poisoning and weight changes**	**Animal**			
	Male 1	*Male 2*	*Female 1*	*Female 2*
hyper/hypoventilation	+	+	+	+
drop of eyelids			+	+
prostration	+	+	+	+
tremor	+	+	+	++
convulsions				+
weight at 24 h (%)	-1.3	-1.2	-3.9	-5.8
weight at 48 h (%)	-2.4	-0.7	-0.4	-5.2

Symptoms of poisoning were semi-quantitatively scored as mild (+), moderate (++), and severe (+++).

Animals were further examined by necropsy 48 h after administration. Plasma (reflecting biochemical changes in the blood) and liver and kidney (elimination organs) were collected to evaluate the toxic potential of the tested compound. At 10 mg/kg, necropsy revealed no macroscopic pathology of internal organs of the chest and abdominal cavity, except for mild hyperemia in the peritoneal cavity and mildly increased content of bile in the duodenum and proximal jejunum of one female mouse (**Table S21**). Blood biochemistry revealed no

significant changes at this dose (**Table S15** and **S22**). Histopathological examination of the liver and kidney showed no tissue damage, only occasional neutrophils in subcapsular liver tissue and perirenal fat neighboring the peritoneal cavity in one male and one female mouse (**Table S21**). The finding implies that the dose of 10 mg/kg can cause weak irritation at the administration site. Despite the histopathological picture, this dose can be considered MTD for single-dose administration.

Table S15. Selected biochemical plasmatic parameters assessed 48 h after i.p. administration of **6** at the dose corresponding to MTD in male and female ICR-CD1 mice (mean ± SEM).

**Parameters**	**Control**	**6 (10 mg/kg)**
Urea (mg/dL)	36.2 ± 8.4	33.8 ± 8.2
Creatinine (μg/dL)	*n.d.*	*n.d.*
ALT (U/L)	31.7 ± 9.6	29.0 ± 6.5
AST (U/L)	56.9 ± 7.4	56.8 ± 6.6
ALP (U/L)	67.5 ± 21.9	68.4 ± 27.2
Amylase (U/L)	3160 ± 500	2960 ± 500
LDH (U/L)	249 ± 47	275 ± 94

Two male and two female mice were in both groups.
No significant differences were found using the Student´s t-test.
n.d. – not determined; values were under the low detection limit

Dose safety verification after oral administration

Dose safety verification was performed in male and female mice after i.g. administration at the dose established as i.p. MTD (10 mg/kg). At this dose, no signs of poisoning occurred. Necropsy revealed no pathology of internal organs of the chest and abdominal cavity. Blood biochemistry showed no significant changes at this dose (**Table S16**). Histopathological examinations of the liver and kidney (excretion organs), terminal esophagus, stomach, duodenum (administration passage, site of administration, and absorption, respectively), and pancreas showed no tissue damage. Altogether, the dose can be considered safe for oral single-dose administration.

Table S16. Selected biochemical plasmatic parameters assessed 48 h after i.g. administration of **6** at 10 mg/kg in male and female ICR-CD1 mice (mean ± SEM).

**Parameters**	**Control**	**6 (10 mg/kg)**
Glucose (mg/dL)	189 ± 14	205 ± 16
Urea (mg/dL)	36.2 ± 2.6	38.6 ± 2.5
Creatinine (μg/dL)	*n.d.*	*n.d.*

ALT (U/L)	53.9 ± 14.2	30.0 ± 1.6
AST (U/L)	80.2 ± 25.0	51.3 ± 7.0
GGT	*n.d.*	*n.d.*
ALP (U/L)	69.3 ± 2.7	61.2 ± 6.2
Amylase (U/L)	2680 ± 620	2630 ± 110
CK (U/L)	263 ± 58	123 ± 25

Two male and two female mice were in both groups.
No significant differences were found using the Student´s t-test.
n.d. – not determined; values were under the low detection limit

Pharmacokinetic study – absorption and brain distribution

The time-dependent plasma/brain concentration curves of **6** after i.p. and i.g. administration were assessed. **Figure 2A (**manuscript**)** shows the concentrations of both curves (plasma and brain) of **6** after i.p. administration. Two curves represent the changes in plasma and brain concentrations. The typical time-dependent plasma profile comprising a short invasive phase with increasing concentration levels (C_{max} 430.09 ± 57.94 ng/mL) to T_{max} (23.33 ± 4.97 min) and the gradual subsequent exponential bio-elimination phase was assessed. The fast absorption with one early T_{max} indicates no **6** precipitation in the peritoneum, so the acute toxicity results can be considered valid. The area under the mean plasma concentration–time curve of **6** from zero up to infinity (AUC_{total}) was 58,804.62 ± 3,318.21 min.ng/mL, and the mean elimination half-life was 161.44 ± 14.25 min.
The compound **6** can target the central nervous system (CNS). Based on our result, the **6** may pass the blood-brain barrier (BBB) via some active transport system. The brain concentration exceeds the plasmatic concentration relatively quickly in the 20th minute after i.p. administration. The maximal brain concentration was approximately two times higher than the maximal plasma concentration: 833.00 ± 62.32 ng/g, and the T_{max} was delayed at 55.00 ± 6.80 min. The relatively high brain concentrations persisted throughout the experiment up to 6 hours after administration; the brain AUC_{total} was 650,923.00 ± 152,288.00 min.ng/g. The Kp (AUC_{brain}/AUC_{plasma}) reached 11.07. These results support the hypothesis that efficient carrier(s) facilitate(s) the transport of **6** across the BBB, in addition to sole passive diffusion.
Moreover, the pharmacokinetic constants after **6** i.p. administration were calculated and are resumed in **Table S17**.

Table S17. Pharmacokinetic parameters obtained after a single i.p. injection of **6** (10 mg/kg) and presented as mean ± SEM (n = 6).

**PLASMA**	
C_{max} (ng/mL)	430.09 ± 57.94
T_{max} (min)	23.33 ± 4.97
AUCtotal (min.ng/mL)	58,804.62 ± 3,318.21
λz (1/min)	0.0045 ± 0.0004
Half-life (min)	161.44 ± 14.25
MRT (min)	217.10 ± 17.65
CL (L/min/kg)	0.17 ± 0.01
Vz/F (L/kg)	40.93 ± 4.95
Vss/F (L/kg)	38.17 ± 4.47
**BRAIN**	
Cmax (ng/g)	833.00 ± 62.32
Tmax (min)	55.00 ± 6.80
AUCtotal (min.ng/g)	650,923.00 ± 152,288.00

C_{max} = max. **6** plasma concentration; T_{max} = time of C_{max}; AUC_{total} = area under the concentration-time curve from zero up to infinity; λz = terminal rate constant; half-life refers to elimination phase; MRT = mean residence time of molecule in the body; CL = clearance; Vz = volume of distribution during terminal phase; Vss = apparent volume of distribution at steady state.

Figure 2B (manuscript**)** shows the concentrations of both curves (plasma and brain) of **6** after i.g. administration. Two curves represent the changes in plasma and brain concentrations. Similar to i.p. administration, the time-dependent plasma profile comprised a short invasive phase with increasing concentration levels (C_{max} 120.47 ± 30.72 ng/mL) to T_{max} (80.00 ± 12.65 min) followed by the gradual subsequent exponential bio-elimination phase. The one early T_{max} indicates relatively fast absorption from the front of the small intestine. The area under the mean plasma concentration-time curve of **6** from zero up to infinity (AUC_{total}) was 34,630.80 ± 4,866.51 min.ng/mL and corresponded to approximately 60% of AUC_{total} after i.p. administration of the same dose. The first-pass effect seems acceptable, and the overall bioavailability is not significantly reduced. It is also good that **6** remains in circulation for a relatively long. It was detected in effective concentration, even at a time corresponding to 8 hours after administration.

Based on our result, the **6** also targets CNS after oral administration. Even in this case, there seems to be permeation by active transport. However, the overall profile is different. The brain concentration exceeds the plasmatic concentration in the 90th minute after oral administration, and the maximal plasma and brain concentrations do not significantly differ. The T_{max} was more delayed at 200.00 ± 60.81 min. On the other hand, the relatively high

brain concentrations persisted throughout the experiment up to 8 hours after administration; the brain AUC_{total} was 68,547.70 ± 9,216.46 min.ng/g. The Kp (AUC_{brain}/AUC_{plasma}) reached only 1.98.

Although the pharmacokinetic profile after i.g. administration is different, **6** can be evaluated as a promising candidate to be administered orally. The compound seems relatively safe, and from the pharmacokinetic point of view, its effectiveness is maintained even after the first pass effect.

Moreover, the pharmacokinetic constants after **6** i.g. administration were calculated and are resumed in **Table S18**.

Table S18. Pharmacokinetic parameters obtained after a single i.g. administration of **6** (10 mg/kg) and presented as mean ± SEM (n = 6).

**PLASMA**	
Cmax (ng/mL)	120.47 ± 30.72
Tmax (min)	80.00 ± 12.65
AUCtotal (min.ng/mL)	34,630.80 ± 4,866.51
λz (1/min)	0.0036 ± 0.0005
Half-life (min)	212.29 ± 26.96
MRT (min)	365.05 ± 18.70
CL (L/min/kg)	0.32 ± 0.05
Vz/F (L/kg)	102.30 ± 22.88
Vss/F (L/kg)	120.48 ± 22.06
**BRAIN**	
Cmax (ng/g)	158.63 ± 20.04
Tmax (min)	200.00 ± 60.81
AUCtotal (min.ng/g)	68,547.70 ± 9,216.46

C_{max} = max. **6** plasma concentration; T_{max} = time of C_{max}; AUC_{total} = area under the concentration-time curve from zero up to infinity; λz = terminal rate constant; half-life refers to elimination phase; MRT = mean residence time of molecule in the body; CL = clearance; Vz = volume of distribution during terminal phase; Vss = apparent volume of distribution at steady state.

Table S19: Signs and symptoms observed in male ICR-CD1 mice when administered with **6** i.p. at 10, 20, 40, and 60 mg/kg doses.

	**Dose (mg/kg)**									
**Symptoms of poisoning and weight changes**	**0 (control)**		**10**		**20**		**40**		**60**	
	Male 1	*Male 2*	*Male 1*	*Male 2*	*Male 1*	*Male 2*	*Male 1*	*Male 2*	*Male 1*	*Male 2*
hyper/hypoventilation			+	+	+	+	++	++	++	++
drop of eyelids					+	+	+	+	+	+
ataxia					+	+			+	
prostration			+	+	++	++	++	++/+++	+++	+++
tremor			+	+	+	+	+	+	+	+
convulsions						+++				
stooped posture										
weight at 24 h (%)	-0.3	+3.1	+1.3	-1.2	-0.2	*	-6.1	-7.0	-3.6	-10.5
weight at 48 h (%)	-2.6	+4.5	+2.4	-0.7	+1.0		-6.7	**	-16.0	-15.6

Symptoms of poisoning were semi-quantitatively scored as mild (+), moderate (++), and severe (+++).

*The male mouse died overnight before the 24-hour check-up.

**The male mouse died overnight before the 48-hour check-up.

Table S20: Signs and symptoms observed in female ICR-CD1 mice when administered with **6** at the dose of 10, 20, 40, and 60 mg/kg.

	**Dose (mg/kg)**									
**Symptoms of poisoning and weight changes**	**0 (control)**		**10**		**20**		**40**		**60**	
	Female 1	*Female 2*	*Female 1*	*Female 2*	*Female 1*	*Female 2*	*Female 1*	*Female 2*	*Female 1*	*Female 2*
hyper/hypoventilation			+	+	+	+	++	++	++	++
drop of eyelids			+	+	+	+	+	+	+	+
ataxia										
prostration			+	+	++	++/+++	++/+++	++/+++	++/+++	++/+++
tremor			+	++	+	++	+	+	+	+
convulsions				+						
stooped posture					+				+	
weight at 24 h (%)	-1.0	+1.3	+3.9	+5.8	-4.2	-1.8	-4.5	-3.5	-5.0	-6.6
weight at 48 h (%)	-2.3	-1.3	+0.4	+5.2	-6.5	0.0	-9.1	-7.1	*	-7.5

Symptoms of poisoning were semi-quantitatively scored as mild (+), moderate (++), and severe (+++).

*The female mouse died overnight before the 48-hour check-up.

Table S21. Necropsy and histopathological findings in liver and renal tissue of male and female ICR-CD1 mice when administered with **6** at the dose of 10, 20, 40, and 60 mg/kg.

**Dose (mg/kg)**	**Animal No.**	**Necropsy**	**Histopathological findings**
0	*Male 1*	no findings	no findings
	Male 2	no findings	no findings
	Female 1	no findings	no findings
	Female 2	no findings	no findings
10	*Male 1*	no findings	occasional neutrophil infiltration in subcapsular liver tissue (+), rare neutrophil infiltration of fat tissue neighboring with peritoneal cavity with neutrophils (+), no cellular damage of liver or renal tissue, no desquamation of peritoneal cells, **dg: mild peritoneal irritation**
	Male 2	no findings	no findings
	Female 1	no findings	no findings
	Female 2	mild hyperemia in the peritoneal cavity, mildly increased content of bile in the duodenum and proximal jejunum	similar to Male 1 (10 mg/kg)
20	*Male 1*	moderate hyperemia in the abdominal cavity, increased bile content in the small intestine	neutrophil infiltration in subcapsular liver tissue (++), occasional necrotic/apoptotic liver cells (+), no renal tissue damage, neutrophil infiltration of fat tissue neighboring with peritoneal cavity (++), desquamation of peritoneal cells covering the fat, fibrin deposits **dg: peritonitis**
	Male 2	*n. p.*	*n. p.*
	Female 1	moderate hyperemia in the abdominal cavity	similar to Male 1 (20 mg/kg)
	Female 2	similar to female 1 (20 mg/kg)	neutrophil infiltration in subcapsular liver tissue (+), occasional necrotic/apoptotic liver cells (+), no renal tissue damage, neutrophil infiltration of fat tissue neighboring with peritoneal cavity (+), desquamation of peritoneal cells covering the fat **dg: peritonitis**

40	*Male 1*	dilatation of small and large intestine loops, moderate hyperemia in the abdominal cavity, hyperemic liver tissue	necrotic liver surface (+++), no renal tissue damage, neutrophil infiltration of fat tissue neighboring with peritoneal cavity (++)<br>**dg: severe peritonitis**
	Male 2	*n. p.*	*n. p.*
	Female 1	similar to male 1 (40 mg/kg)	necrotic liver surface (+++), no renal tissue damage, neutrophil infiltration of fat tissue neighboring with peritoneal cavity (+++) with desquamation of peritoneal cells and fibrin deposits<br>**dg: severe peritonitis**
	Female 2	dilatation of the stomach, small and large intestine loops, and gall bladder, increased bile content in stool, moderate hyperemia and fluid in the abdominal cavity, hyperemic liver tissue	similar to Female 2 (40 mg/kg)
60	*Male 1*	dilatation of small and large intestine loops and gall bladder, increased bile content in stool, moderate hyperemia in the abdominal cavity, hyperemic liver tissue	necrotic liver surface with subcapsular necroses reaching deep into the tissue (+++), no renal tissue damage, neutrophil infiltration of fat tissue neighboring with peritoneal cavity (+++) with desquamation of peritoneal cells, and fibrin deposits<br>**dg: severe peritonitis**
	Male 2	similar to Male 1 (60 mg/kg)	similar to Male 1 (60 mg/kg)
	Female 1	*n. p.*	*n. p.*
	Female 2	similar to Male 1 (60 mg/kg)	similar to Male 1 (60 mg/kg)

n. p. – not performed due to the animal's death before the examination.

Histopathological findings were semi-quantitatively scored as mild (+), moderate (++), and severe (+++).

Table S22. Biochemistry of blood plasma of male and female ICR-CD1 mice when administered with **6** at the dose of 10, 20, 40, and 60 mg/kg. Statistical analysis was performed using the Student´s t-test compared with the control group.

	Dose (mg/kg)				
**Parameters**	**0 (control)**	**10**	**20 ***	**40 ***	**60 ***
Urea (mg/dL)	36.2 ± 8.4	33.8 ± 8.2	31.8 ± 6.7	93.6 ± 60.4 †	142.9 ± 145.2 †
Creatinine (µg/dL)	*n.d.*	*n.d.*	*n.d.*	*n.d.*	*n.d.*
ALT (U/L)	31.7 ± 9.6	29.0 ± 6.5	27.0 ± 15.6	57.1 ± 16.5	230.4 ± 140.7 †
AST (U/L)	56.9 ± 7.4	56.8 ± 6.6	55.6 ± 6.7	82.4 ± 54.0 †	318.0 ± 213.7 †
ALP (U/L)	67.5 ± 21.9	68.4 ± 27.2	60.4 ± 28.6	63.5 ± 26.9	71.4 ± 23.4
Amylase (U/L)	3158 ± 497	2955 ± 501	3129 ± 444	3485 ± 894	3723 ± 448
LDH (U/L)	249 ± 47	275 ± 94	223 ± 38	511 ± 423 †	1101 ± 562†

Number of animals per group N = 4, *N = 3.

n.d. – not determined; values were under a low detection limit (56 µg/dL).

† significant differences compared to the control group ($p \leq 0.05$).

# Pharmacodynamic study.

Materials and methods

Animals and housing conditions

In all pharmacodynamic *in vivo* experiments, except for the Barnes maze (BM) task, adult male Albino Swiss (CD-1) mice weighing between 18 and 22 g and purchased from the Animal Breeding Farm of the Faculty of Pharmacy, Jagiellonian University Medical College were used. In BM task, C57BL/6J mice of the same weight (Animalab, Poland) were used. Animals were housed in cages (10 mice per cage) at a constant temperature of 22 ± 2 °C, humidity of 55 ± 10%, and a light/dark (12:12) cycle (lights on at 7 AM). Before the experiments, the animals had unlimited access to food and water. Specified conditions for the maintenance of mice were ensured throughout the experiments, including tree bedding (Transwior, Poland) and cage enrichment (tunnels, nesting material, wooden igloos, etc.). For the behavioral test, the mice were selected randomly; each group consisted of 7-12 mice. The experiments were performed between 9 AM and 3 PM. After *in vivo* tests, the animals were immediately euthanized.

Ethical approval statement

The procedures for the maintenance and treatment of laboratory animals were approved by the 1st Local Ethics Committee of the Jagiellonian University in Krakow (Approvals No. 524/2021, 618/2022), and the treatment of animals was in full accordance with ethical standards laid down in respective Polish and EU regulations (Directive 2010/63/EU).

Drugs and dose selection for the *in vivo* tests

For *in vivo* assays **6** was suspended in 1% Tween 80 (Sigma Aldrich, Poland) and administered intraperitoneally (i.p.) 60 min before the acquisition trial of the passive avoidance (PA) task, BM task and 60 min before the forced swim test (FST), four-plate test (FPT) and elevated plus maze (EPM) test. Doses 10 mg/kg and 30 mg/kg were the starting

doses for behavioral assays. Lower doses were also assessed if at least one of them was active.

Scopolamine hydrobromide used at the dose of 1 mg/kg for the induction of learning and memory deficits was supplied by Sigma Aldrich (Poland). The dose of scopolamine was chosen based on our previous studies[44,45] and available literature data.[46] For the *in vivo* tests, scopolamine was prepared in distilled water (Polfa Kutno, Poland) and was administered subcutaneously (s.c.) 30 min before the acquisition (training) trial of the PA and BM tasks.

Behavioral tests

Effect on fear-motivated contextual memory - passive avoidance task

The PA test was conducted according to a previously described protocol used in our laboratory to assess the influence of tested compounds on fear-motivated learning and memory in mice.[44] The PA apparatus (Panlab Harvard Apparatus, Spain) consists of a large white-colored illuminated compartment (26 cm × 26 cm × 34 cm) and a small black-colored compartment (13 cm × 7.5 cm × 7.5 cm), which are separated from each other by a guillotine gate (5 cm × 5 cm). The PA task is divided into two trials (i.e., the acquisition trial and the retention trial) conducted 24 h apart.

On the first day, during the acquisition trial (a conditioning phase), the test compound **6**, scopolamine, and vehicle were injected. Then, each mouse was placed into the white compartment, and a 30-s habituation period started (the guillotine gate was closed). After that, the guillotine gate was opened, and the mice were tested during the 180-s testing period. As soon as the mouse entered the black compartment of the PA device, the guillotine gate was closed, and an electrical shock (intensity: 0.2 mA, duration: 2 s) was automatically applied through the grid floor. The latency between opening the guillotine gate and the mouse entering the black compartment was measured for each mouse. On the next day, during the retention (drug-off) trial, the mice were placed again into the white compartment for a 30-s habituation period, and the latency to enter the black compartment was measured for each mouse. Increased latency to enter the black compartment in the retention trial compared to that in the acquisition trial was a measure of antiamnesic (procognitive) properties of **6**.

Effect on spatial learning and memory – Barnes maze task

Spatial learning and memory in mice were also assessed using the BM task.[47] This task was performed with the BM apparatus (Panlab-Harvard Apparatus, Spain). It consists of a circular, dry, open platform surface equipped with 18 holes around the perimeter of the platform and a small dark recessed chamber located under one of them. Visual extra-maze cues (pictures of colored geometric figures with contrasting backgrounds placed at a distance of 1 m from the platform) were provided to facilitate learning. A weak aversive stimulus (light 600 lx) was applied to increase the animals' motivation to escape from the circular platform.

The spatial acquisition trial was performed during the first 4 days of the experiment. On the first day of the test, an additional adaptation period preceded a proper trial. In this phase, mice were individually placed in a cylindrical black start chamber in the middle of the circular maze. Ten seconds later, the mouse was released from the start chamber, the light was turned on, and the animal was gently guided to the escape box (a small dark recessed chamber under the BM platform), where it remained for the next 2 min. This phase was not preceded by drug injection.

In the spatial acquisition phase, each mouse was subjected to four trials daily, keeping 15-min intervals between each trial. Mice were injected with **6** or 1% Tween 80 60 min before, and with scopolamine hydrobromide (1 mg/kg) 30 min before the first trial on each day of the acquisition phase. In every trial, the mouse was placed in a cylindrical black start chamber in the middle of BM. After 10 s the mouse was released from the start chamber, the light was turned on, and the mouse was allowed to explore the maze for 180 s. During this phase, the number of primary errors (i.e., the number of errors before the mouse reached the escape box) and primary latencies to find the escape box (i.e., the time required to find the escape box) were measured. If the mouse did not find the escape box during 180 s, it was gently guided towards it. Immediately after the mouse entered the box, the light was turned off, and the mouse was allowed to stay in the box for 1 min. On days 5 and 12 the assessment of reference short-term and long-term memory was conducted, respectively (a drug-off probe trial). The arrangement of BM remained unchanged, and each mouse was placed in a cylindrical black start chamber. After its removal, the mouse was allowed to explore the maze for the next 90 s. Primary errors and latency in finding the former escape box location were recorded.

Effect on depressive-like behavior - forced swim test

The experiment was carried out according to Porsolt et al.[48] with small modifications. Briefly, mice were individually placed in a glass cylinder (25 cm high, 10 cm in diameter) containing 11 cm of water maintained at 23–25 °C and were left therein for 6 min. A mouse was regarded as immobile when it remained floating on the water, making only small movements to keep its head above it. The total duration of immobility was measured during the final 4 min of a 6-min test session.

Effect on anxiety - four-plate test

The four-plate apparatus (FPT; Bioseb, France) consists of a cage (25 × 18 × 16 cm) that is floored with four rectangular metal plates (11 × 8 cm). The plates are separated from one another by a 4 mm gap, and they are connected to an electroshock generator. The test was performed according to Bourin et al.[49] After the habituation period (15 s), each mouse was subjected to an electric shock (0.8 mA, 0.5 s) when crossing from one plate to another (two limbs on one plate and two on another). The number of punished crossings was counted during 60 s.

Effect on anxiety - elevated plus maze

The elevated plus maze for mice consists of two opposing open (30 cm x 5 cm) and two enclosed (30 cm x 5 cm x 25 cm), likewise opposing open arms. The dimensions of the central field, which connects the open and closed arms, are 5 × 5 cm. In this test, each mouse was individually placed at the central field of the apparatus with the head turned towards one of the closed arms. The animal's behavior was observed for 5 min. The following parameters were measured in this test: time spent in open arms of the EPM device and the number of entries to open arms. To exclude the impact of excrements or smell left by a previous mouse on the behavior of the next one, the device was cautiously cleaned after each session.[50]

Effect on locomotor activity

The locomotor activity test was performed using activity cages (40 cm x 40 cm x 30 cm, supplied with I.R. beam emitters) (Activity Cage 7441, Ugo Basile, Italy) connected to a counter to record light-beam interrupts. Before the experiment, the mice were intraperitoneally pretreated with **6** or vehicle and then individually placed in the activity cages in a sound-attenuated room. The animals' movements (i.e., the number of light-beam

crossings) were counted during the next 60 min of the test. Before the experiment, the mice were habituated to activity cages for 15 min.[50]

Data analysis

For the analysis of data obtained in the *in vivo* tests GraphPad Prism software (v. 9.0, CA, USA) was used. Numerical results from the tests were expressed as mean ± standard error of the mean (SEM). Repeated measures ANOVA or one-way ANOVA followed by Dunnett's *post hoc* test were used for the statistical analysis of the results. $P < 0.05$ was considered significant.

Results of pharmacodynamic *in vivo* studies.

Effect on fear-motivated memory – passive avoidance task

In this fear-motivated task the effect of **6** on scopolamine-induced cognitive dysfunction was assessed. A significant overall effect of treatment was observed ($F[10.172] = 17.15$, $p < 0.0001$). Time effect and drug x time interaction were also significant ($F[1.172] = 19.44$, $p < 0.0001$, and $F[10.172] = 16.48$, $p < 0.0001$, respectively). In the acquisition trial, we did not observe significant inter-group differences in the step-through latency between the vehicle-treated mice and the scopolamine-treated group ($p > 0.05$). In contrast to this, statistically significant differences were observed in the retention trial between scopolamine-treated control and vehicle-treated mice not treated with scopolamine ($p < 0.0001$), as well as between scopolamine-treated control mice and mice that received combined scopolamine and **6** (**Figure S53**) at dose 10 mg/kg ($p < 0.0001$).

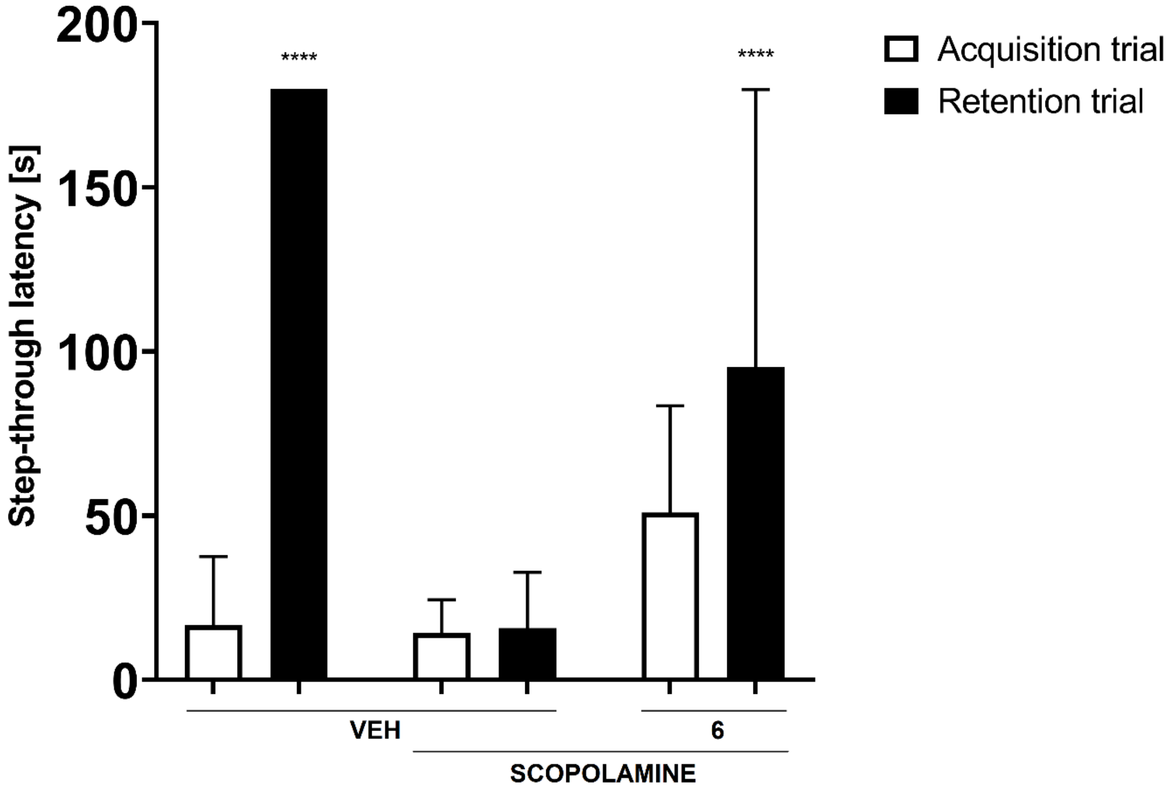

Figure S53. Effect of **6** on fear-motivated contextual learning and memory measured using the PA task in a mouse model of scopolamine-induced amnesia. Results are shown as mean step-through latency (± SEM) assessed in the acquisition trial and 24 h later (in the retention trial) for n = 7-12 mice. Statistical analysis: repeated measures ANOVA followed by Dunnett's *post hoc* comparison. Significance *vs.* scopolamine-treated control mice in the retention trial: **** $p < 0.0001$.

Effect on spatial learning and memory – Barnes maze task

Acquisition phase

In the acquisition trial of the BM task, repeated measures ANOVA showed a statistically significant effect of treatment on the latency to find the escape box ($F[2.96]=14.83$, $p < 0.0001$). The time effect was statistically significant ($F[3.96]=3.118$, $p < 0.05$). The drug x time interaction was also statistically significant ($F[6.96]=2.274$, $p < 0.05$).

Dunnett's *post hoc* analysis revealed statistically significant differences in the latency to find the escape box between the control group that did not receive scopolamine and the

scopolamine-treated control group on days 3 ($p < 0.05$) and 4 ($p < 0.01$) of the BM task. Statistically significant differences were also observed on day 4 of the BM task ($p < 0.05$) between the group that received scopolamine and **6** at a dose of 10 mg/kg and the scopolamine-treated control group. These results are presented in **Figure 3** (manuscript).

In the acquisition trial of the BM task, repeated measures ANOVA revealed a statistically significant effect of treatment on the number of errors made before reaching the escape box ($F[2.93] = 7.653$, $p < 0.001$). Time effect was also significant ($F[3.93] = 4.021$, $p < 0.01$) but the drug × time interaction was not ($F[6.93] = 1.334$, $p = 0.25$). Dunnett's *post hoc* analysis showed no statistically significant differences in the number of errors made between the two control groups and between the control group that received scopolamine and a group that received scopolamine and compound **6** at a dose of 10 mg/kg (**Figure S54**).

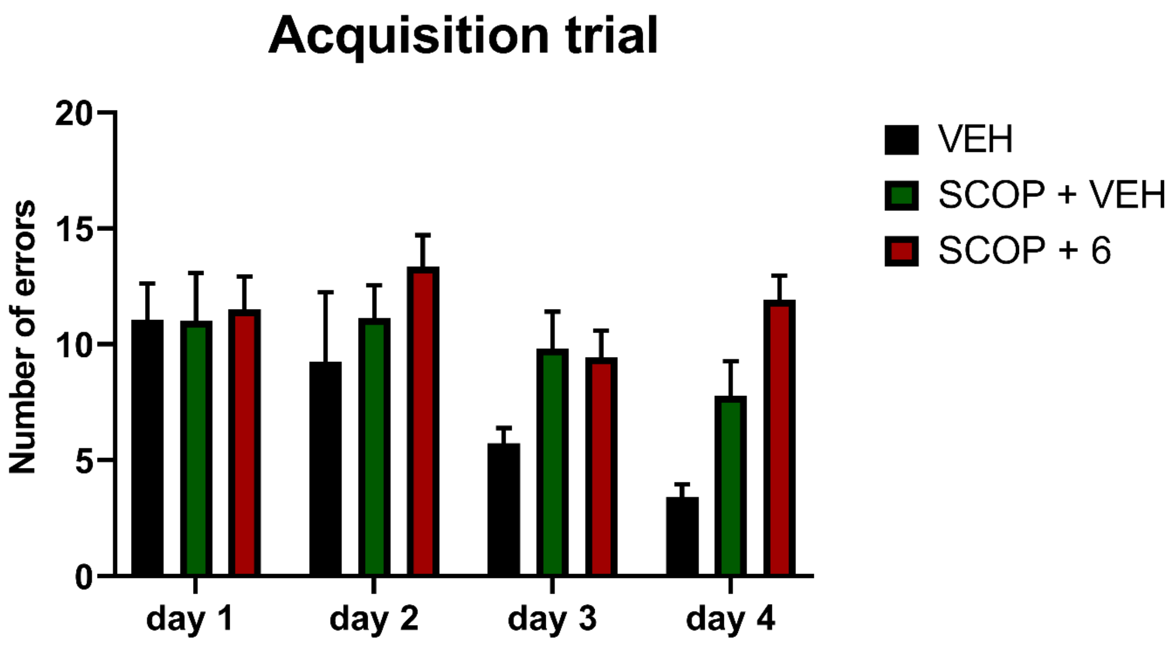

Figure S54. Effect of **6** on the number of errors made before reaching the escape box assessed during the acquisition phase (days 1-4) of the BM task. The results are presented as the mean number of errors made before reaching the escape box ± SEM. Statistical analysis: repeated measures ANOVA and Dunnett's *post hoc* test: $p > 0.05$.

*Effect of **6** on memory retrieval measured on days 5 and 12*

On day 5 (a drug-off trial, assessment of short-term memory), one-way ANOVA showed a statistically significant difference among the groups in the latency to find the former location

of the escape box ($F[2.21] = 3.315$, $p < 0.05$), but not in the number of errors made before finding the escape box ($F[2.23] = 1.901$, $p > 0.05$).

Post hoc analysis of the results obtained on day 5 showed statistically significant differences in the latency to find the former escape box location between the two control groups ($p < 0.05$) but not between the scopolamine-treated control and scopolamine and **6**-treated mice (**Figure 3B, manuscript**). On day 5, there were no significant differences among groups regarding the number of errors made (**Figure 3C, manuscript**).

On day 12 (no drug administration, assessment of long-term memory), one-way ANOVA showed a statistically significant effect of treatment with scopolamine and **6** on both latencies to find the former location of the escape box ($F[2.24] = 6.928$, $p < 0.01$) and the number of errors made before reaching the former escape box location ($F[2.22] = 23.60$, $p < 0.0001$).

Post hoc analysis of the results obtained on day 12 showed statistically significantly reduced latency to find the former escape box location in the control group compared to the group that received scopolamine on days 1-4 ($p < 0.01$). A similar effect was noted in mice treated with **6** and scopolamine compared to the control group that received scopolamine ($p < 0.05$, **Figure 3D, manuscript**).

Post hoc analysis of the results obtained on day 12 showed a significantly reduced number of errors made in the control group that was not treated with scopolamine compared to the scopolamine-treated control ($p < 0.0001$). In addition, in mice treated with scopolamine and **6**, the dose of 10 mg/kg significantly reduced number of errors made compared to the scopolamine-treated control ($p < 0.0001$, **Figure 3E, manuscript**).

Antidepressant-like activity - forced swim test

One-way ANOVA revealed a significant effect of treatment on immobility duration measured in the FST ($F[4,41]=2.624$, $p < 0.05$). Dunnett's *post hoc* test revealed that **6** at a dose of 1 mg/kg statistically significantly ($p < 0.05$) reduced immobility as compared to control (**Figure 4D, manuscript**). This indicates a potential antidepressant-like activity of this dose of **6** in mice.

Anxiolytic- like activity - four-plate test

One-way ANOVA revealed a significant effect of treatment on the number of punished crossings measured in the FPT ($F[3,32]=6.225$, $p < 0.01$). Dunnett's *post hoc* revealed that **6** at doses 0.1, 1 and 10 mg/kg statistically significantly ($p < 0.05$) increased the number of punished crossings as compared to control (**Figure 4C, manuscript**). This indicates for a potential anxiolytic-like activity of these doses of **6** in mice.

Anxiolytic-like activity - Elevated plus maze test

Compound **6** at doses 0.1, 1, and 10 mg/kg was also assessed for its anxiolytic-like activity in the mouse EPM test. One-way ANOVA revealed an overall effect of treatment on time spent in open arms ($F[3.27] = 7.770$, $p < 0.001$) and on the number of open-arm entries ($F[3.27] = 3.691$, $p < 0.05$).

As shown in **Figure 4A (manuscript)**, compared to control, **6** at doses 0.1 and 1 mg/kg significantly ($p < 0.05$) prolonged time spent in open arms of the EPM device and as demonstrated in **Figure 4B (manuscript)**, compared to control, **6** at doses 1 and 10 mg/kg significantly ($p < 0.05$) increased the number of open arm entries. This confirmed its anxiolytic-like properties in mice (also observed in the FPT).

Effect on locomotor activity

For compound **6**, the locomotor activity test was performed for two doses, 10 and 30 mg/kg (**Figure S55**). None of these doses significantly affected the locomotor activity of mice ($F[2.13] = 2.220$, $p > 0.05$)

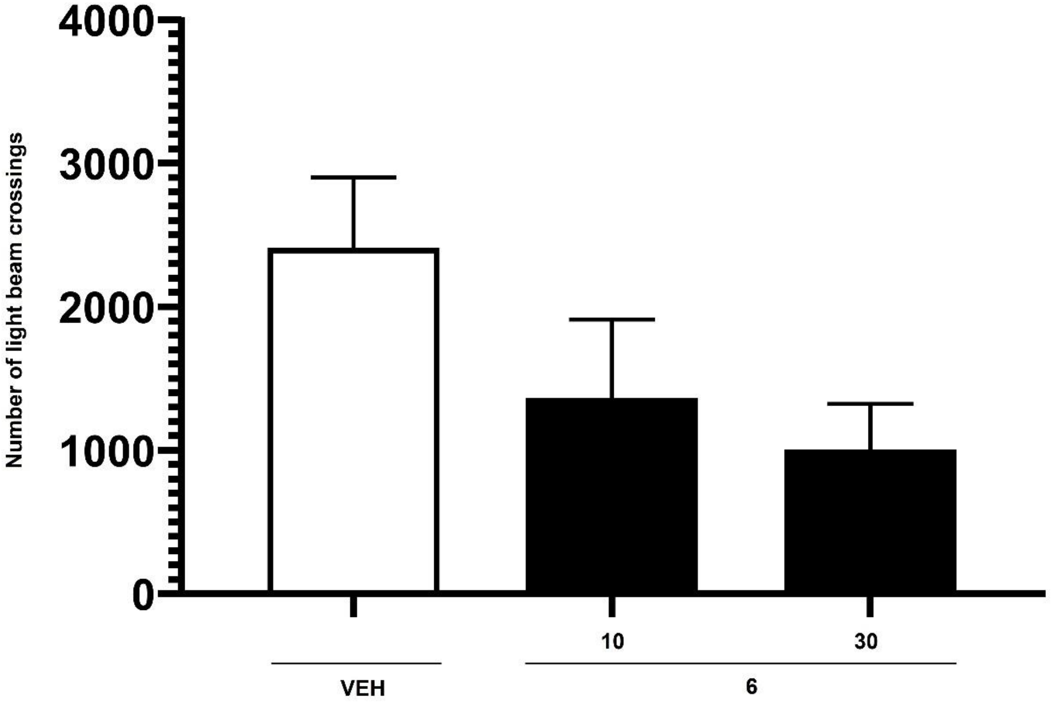

Figure S55. Effect of **6** on spontaneous locomotor activity in mice. Results are presented as the mean number of light-beam crossings ± SEM measured during 60 min of the locomotor activity test. Statistical analysis: one-way analysis of variance and Dunnett's *post hoc* test: $p > 0.05$.

Literature

[25] A. Pasieka, D. Panek, J. Jończyk, J. Godyń, N. Szałaj, G. Latacz, J. Tabor, E. Mezeiova, F. Chantegreil, J. Dias, D. Knez, J. Lu, R. Pi, J. Korabecny, X. Brazzolotto, S. Gobec, G. Höfner, K. Wanner, A. Więckowska, B. Malawska, Eur J Med Chem 2021, 218, DOI 10.1016/J.EJMECH.2021.113397.

[26] F. Nachon, Y. Nicolet, N. Viguié, P. Masson, J. C. Fontecilla-Camps, O. Lockridge, Eur J Biochem 2002, 269, 630–637.

[27] X. Brazzolotto, M. Wandhammer, C. Ronco, M. Trovaslet, L. Jean, O. Lockridge, P. Y. Renard, F. Nachon, FEBS J 2012, 279, 2905–2916.

[28] P. Legrand, "XDSME: XDS Made Easier (2017) GitHub repository," 2017.

[29] D. Liebschner, P. V. Afonine, M. L. Baker, G. Bunkoczi, V. B. Chen, T. I. Croll, B. Hintze, L. W. Hung, S. Jain, A. J. McCoy, N. W. Moriarty, R. D. Oeffner, B. K. Poon, M. G. Prisant, R. J. Read, J. S. Richardson, D. C. Richardson, M. D. Sammito, O. V. Sobolev, D. H. Stockwell, T. C. Terwilliger, A. G. Urzhumtsev, L. L. Videau, C. J. Williams, P. D. Adams, Acta Crystallogr D Struct Biol 2019, 75, 861–877.

[30] A. Casañal, B. Lohkamp, P. Emsley, Protein Sci 2020, 29, 1069–1078.

[31] N. W. Moriarty, R. W. Grosse-Kunstleve, P. D. Adams, Acta Crystallogr D Biol Crystallogr 2009, 65, 1074–1080.

[32] G. L. Ellman, K. D. Courtney, V. Andres, R. M. Featherstone, Biochem Pharmacol 1961, 7, 88–95.

[33] T. Wu, Z. J. Gale-Day, J. E. Gestwicki, Protein Sci 2024, 33, DOI 10.1002/PRO.5022.

[34] F. H. Niesen, H. Berglund, M. Vedadi, Nat Protoc 2007, 2, 2212–2221.

[35] A. Kragler, G. Höfner, K. T. Wanner, Eur J Pharmacol 2005, 519, 43–47.

[36] S. Schmitt, G. Höfner, K. T. Wanner, ChemMedChem 2015, 10, 1498–1510.

[37] C. Zepperitz, G. Höfner, K. T. Wanner, ChemMedChem 2006, 1, 208–217.

[38] L. Muckova, N. Vanova, J. Misik, D. Herman, J. Pejchal, D. Jun, Toxicol In Vitro 2019, 56, 110–117.

[39] S. Robinson, K. Chapman, S. Hudson, S. Sparrow, D. Spencer-Briggs, A. Danks, R. Hill, D. Everett, B. Mulier, S. Old, C. Bruce, Guidance on Dose Level Selection for Regulatory General Toxicology Studies for Pharmaceuticals., National Centre For The Replacement, Refinement And Reduction Of Animals In Research Laboratory Animal Science Association (NC3Rs)/Laboratory Animal Science Association (LASA), London, 2009.

[40] J. Misik, E. Nepovimova, J. Pejchal, J. Kassa, J. Korabecny, O. Soukup, Curr Alzheimer Res 2018, 15, 552–560.

[41] J. Pejchal, J. Novotný, V. Mařák, J. Österreicher, A. Tichý, J. Vávrová, Z. Šinkorová, L. Zárybnická, E. Novotná, J. Chládek, A. Babicová, K. Kubelková, K. Kuča, Int J Radiat Biol 2012, 88, 348–358.

[42] FDA, "Guidance for Industry, Bioanalytical Method Validation," 2018.

[43] EMA, "Guideline on Bioanalytical Method Validation," 2011.

[44] K. Sałat, A. Podkowa, S. Mogilski, P. Zaręba, K. Kulig, R. Sałat, N. Malikowska, B. Filipek, Pharmacol Rep 2015, 67, 1155–1162.

[45] U. Košak, B. Brus, D. Knez, R. Šink, S. Žakelj, J. Trontelj, A. Pišlar, J. Šlenc, M. Gobec, M. Živin, L. Tratnjek, M. Perše, K. Sałat, A. Podkowa, B. Filipek, F. Nachon, X. Brazzolotto, A. Więckowska, B. Malawska, J. Stojan, I. M. Raščan, J. Kos, N. Coquelle, J. P. Colletier, S. Gobec, Scientific Reports 2016 6:1 2016, 6, 1–16.

[46] I. Klinkenberg, A. Blokland, Psychopharmacology (Berl) 2011, 215, 549.

[47] A. Meden, D. Knez, N. Malikowska-Racia, X. Brazzolotto, F. Nachon, J. Svete, K. Sałat, U. Grošelj, S. Gobec, Eur J Med Chem 2020, 208, DOI 10.1016/J.EJMECH.2020.112766.

[48] R. D. Porsolt, G. Anton, N. Blavet, M. Jalfre, Eur J Pharmacol 1978, 47, 379–391.

[49] M. Bourin, F. Masse, E. Dailly, M. Hascoët, Pharmacol Biochem Behav 2005, 81, 645–656.

[50] K. Sałat, A. Podkowa, P. Kowalczyk, K. Kulig, A. Dziubina, B. Filipek, T. Librowski, Pharmacol Rep 2015, 67, 465–472.
